# The Mechanism for RNA Recognition by ANTAR Regulators of Gene Expression

**DOI:** 10.1371/journal.pgen.1002666

**Published:** 2012-06-07

**Authors:** Arati Ramesh, Sruti DebRoy, Jonathan R. Goodson, Kristina A. Fox, Herbey Faz, Danielle A. Garsin, Wade C. Winkler

**Affiliations:** 1 Department of Biochemistry, The University of Texas Southwestern Medical Center, Dallas, Texas, United States of America; 2 Department of Microbiology and Molecular Genetics, The University of Texas Health Science Center at Houston, Houston, Texas, United States of America; 3 Department of Cell Biology and Molecular Genetics, The University of Maryland, College Park, Maryland, United States of America; Agency for Science, Technology, and Research, Singapore

## Abstract

ANTAR proteins are widespread bacterial regulatory proteins that have RNA–binding output domains and utilize antitermination to control gene expression at the post-initiation level. An ANTAR protein, EutV, regulates the ethanolamine-utilization genes (*eut*) in *Enterococcus faecalis*. Using this system, we present genetic and biochemical evidence of a general mechanism of antitermination used by ANTARs, including details of the antiterminator structure. The novel antiterminator structure consists of two small hairpins with highly conserved terminal loop residues, both features being essential for successful antitermination. The ANTAR protein dimerizes and associates with its substrate RNA in response to signal-induced phosphorylation. Furthermore, bioinformatic searches using this conserved antiterminator motif identified many new ANTAR target RNAs in phylogenetically diverse bacterial species, some comprising complex regulons. Despite the unrelatedness of the species in which they are found, the majority of the ANTAR–associated genes are thematically related to nitrogen management. These data suggest that the central tenets for gene regulation by ANTAR antitermination occur widely in nature to specifically control nitrogen metabolism.

## Author Summary

In bacteria, two-component regulatory systems comprise the primary mechanisms for how microorganisms respond to changes in their environment. These signal transduction systems rely upon phosphotransfer between two conserved proteins, a histidine kinase and a response regulator, to propagate the signal and affect cellular biology. Phosphorylation of the response regulator has been shown in many systems to control DNA–binding activity, protein–protein interactions, or enzymatic activity. However, in this study, we discover a general RNA substrate for a large family of putative RNA–binding response regulator proteins called ANTAR proteins. By identifying the general architecture of this RNA recognition element, our bioinformatic searches were then able to discover many more examples of these RNA motifs in bacteria. Indeed, our data together revealed that the regulatory relationship between ANTAR proteins and the RNA motif identified in this study is widespread among phylogenetically diverse bacteria for control of numerous nitrogen metabolism genes.

## Introduction

When producing a functional protein from a gene sequence, regulation can occur by a multitude of mechanisms at all levels of the process. The events surrounding control of transcription initiation are arguably the best studied; however, many genes are also controlled by post-initiation regulatory mechanisms. For example, transcription elongation is oftentimes subjected to post-initiation control by the presence of intrinsic terminator hairpins in the nascent transcript. Typically, intrinsic terminator sites occur at ends of operons in order to promote site-specific dissociation of RNA polymerase. However, they are also frequently arranged upstream of open reading frames (ORFs), typically within 5′ leader regions, where they participate in signal-responsive regulatory mechanisms. In certain instances, proteins with RNA-binding domains interact with these 5′ leader regions to influence terminator formation and thereby control downstream gene expression [1]. One mechanism by which this can occur is through the formation of an antiterminator, which is a structural element that is mutually exclusive with respect to formation of the terminator hairpin. For a few well-studied systems, association of the appropriate RNA-binding protein influences which of these RNA structural elements are formed. For example, certain members of the BglG/SacY protein family contain the PTS regulation domain, which is an RNA-binding domain that associates with a characteristic antiterminator element overlapping a mutually exclusive, adjacent terminator site. Phosphorylation of the PTS domain by the appropriate carbohydrate transport system controls the RNA-binding activity, thereby coupling signal-responsiveness to direct stabilization of the antiterminator structure. In contrast, the *trp* RNA-binding attenuation protein (TRAP) associates with a tandem series of triplet sequences in order to prevent formation of a default antiterminator element, thereby permitting formation of an alternate intrinsic terminator structure [1]–[3].

**Figure 1:**
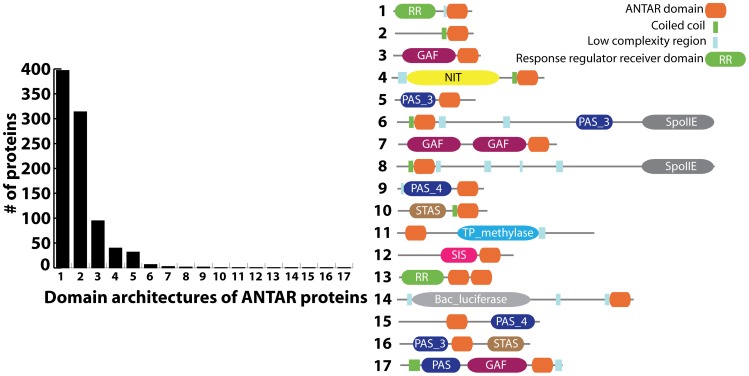
Distribution of ANTAR-containing proteins according to their domain organization (Pfam: PF03861). The bar graph shows the number of ANTAR proteins for each of 17 domain architectures that have been identified. The latter are schematically represented to the right of the bar graph.

Another important family of proteins with putative RNA-binding activity contains the AmiR and NasR Transcriptional Antiterminator Regulator domain (ANTAR) [4]. The ANTAR domain is composed of three helices with five strictly conserved residues (three alanines, one alanine/serine and one aromatic residue) that are exposed in the three-helical structure [5]. Sequence homology based searches have predicted more than 1100 occurrences of the ANTAR domain, widely distributed across at least 644 bacterial species (Figure 1; http://pfam.sanger.ac.uk/; Pfam: PF03861). ANTAR-containing proteins typically occur as multi-domain proteins. A significant class of ANTAR proteins appear to possess an N-terminal domain that resembles a pseudo-receiver domain capable of protein-protein interactions [5]. This class of proteins may therefore regulate gene-expression via interactions with a modulator protein, which itself may possess signal-sensing function. For example, the ANTAR protein AmiR from *Pseudomonas aeruginosa* dimerizes upon binding two molecules of its negative regulator AmiC. Under inducing conditions AmiC binds a small amide compound, allowing association of AmiR with the 5′ leader of the appropriate target mRNA. This has been hypothesized to prevent formation of an intrinsic terminator. However, the molecular mechanism of antitermination, including the AmiR RNA recognition determinants, has yet to be revealed [6]–[7].

The ANTAR domain also occurs in combination with a diverse set of signal-sensing domains (Figure 1). For example, NasR, a protein with a nitrate and nitrite sensing NIT domain fused to an ANTAR domain, regulates the *nasFEDCBA* operon in *Klebsiella* species, which is required for nitrogen assimilation [8]. In the presence of nitrate, NasR is activated and binds to the 5′ leader region of the nascent *nasF* transcript. Association of NasR inhibits formation of a transcription terminator within the 5′ leader region, thereby allowing synthesis of the downstream *nas* operon. Like the AmiR system, the molecular mechanism of antitermination, including the NasR RNA determinants, have not been identified. In fact, it has been speculated that the mechanism might not even involve formation of a specific antiterminator structure, in contrast to the BglG/SacY family of antiterminators [9].

ANTAR also occurs in combination with the ubiquitous PAS (found in *P*eriod clock protein, *A*ryl hydrocarbon receptor and *S*ingle minded protein) and GAF (in c*G*MP phosphodiesterases, *A*denylate cyclases and *F*hlA proteins) domains (Figure 1). While no specific examples of ANTARs in combination with PAS or GAF domains have been characterized, these respective sensory domains are generally known to be responsive to a diverse array of cellular responses including changes in redox, light intensity, and aerobiosis. They have also been shown to respond to co-factors such as flavin, heme, or second messenger molecules, among many other molecular ligands [10]–[11]. Therefore, the domain organization of ANTAR-containing proteins raises the intriguing possibility that the ANTAR domain may function as a global regulatory module, partnering directly or indirectly with a diverse set of signal-sensing domains to respond to a broad range of cellular signals.

The largest individual class (nearly 50%) of ANTAR-containing proteins is comprised of response regulators that are part of bacterial two-component regulatory systems (TCS). TCS typically consist of a sensor histidine kinase that undergoes autophosphorylation upon sensing its signal and in turn transfers the phosphoryl group to the receiver domain of a cognate response regulator [12]–[13]. The phosphoryl transfer reaction subsequently activates the effector domain of the protein. These effector domains control signaling pathways through a variety of mechanisms, such as promoting DNA-binding activity, altering protein-protein interactions or affecting enzymatic activity [14]. In contrast, ANTAR-containing response regulator proteins would be postulated to regulate gene expression via RNA-binding mechanisms. This class of response regulators is the least understood, despite the fact that their widespread occurrence in bacterial genomes suggests they are broadly important in gene regulation.

**Figure 2:**
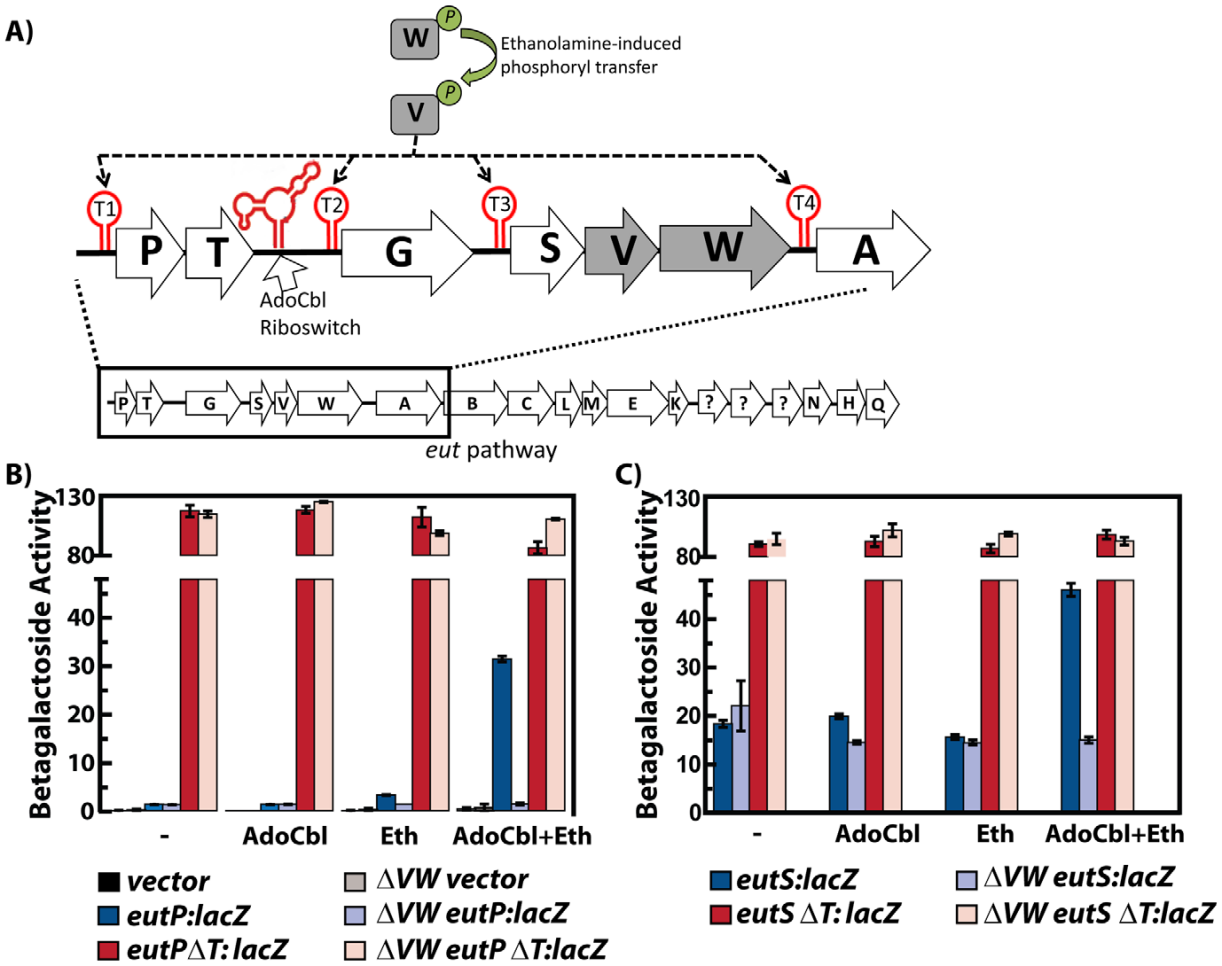
Multiple input signals, along with protein and RNA elements, regulate the *eut* locus in *Enterococcus faecalis*. A) The organization of the *E. faecalis eut* locus is shown schematically. The EutV/EutW two component regulatory system responds to ethanolamine to stimulate EutW autophosphorylation followed by phosphoryl transfer to EutV [15], [17]. Phosphorylated EutV is hypothesized to prevent formation of four different intrinsic terminator sites (red) within the *eut* pathway [17], [18]. Additionally, an AdoCbl-sensing riboswitch is located upstream of *eutG* [14], [16]. B) Expression of *lacZ* translational fusions to the *eutP* 5′ leader region is shown as bar graphs and is described in the text. Each fusion is represented by a color with the darker shade indicating the wild type background and the lighter shade designating the *eutVW* background. Presence of both AdoCbl and ethanolamine was required for induction of *eutP* containing the wild-type leader sequence (blue). Deletion of the EutV/EutW two-component regulatory system abolished *eutP* induction (light blue). Deletion of the terminator in the leader, *eutP*ΔT abolished EutV/EutW dependency (red/light red). The vector control in both backgrounds is shown in black/gray. (C) Expression of *lacZ* translational fusions to the *eutS* 5′ leader region. Color scheme is described in the figure.

EutV, a representative of ANTAR-containing response regulators, was discovered to regulate the ethanolamine utilization operon (*eut*) in *Enterococcus faecalis* and this mode of regulation appears to be conserved in many Firmicutes that contain *eut* operons [15]–[17]. For *E. faecalis*, the corresponding sensor kinase, EutW, undergoes autophosphorylation in response to ethanolamine whereupon the phosphoryl group is transferred to EutV [15], [17]. Phosphorylated EutV is postulated to disrupt terminator sites located just upstream of each of the genes *eutP*, *eutG*, *eutS* and *eutA* (Figure 2A); its association is therefore predicted to activate downstream gene expression [15]–[17]. These locations within the *eut* operon were found to share a common 13-nucleotide sequence (AGCAANGRRGCUY) overlapping the 5′-proximal portion of their corresponding intrinsic terminator elements. We previously proposed that these sites could serve as part of the recognition sequence for ANTAR-based regulators in order to promote antitermination and allow production of the downstream transcript [17]. Recent work investigating the regulation of *eutG* in *E. faecalis* supports the model that antitermination occurs at this consensus sequence [18]. However, no functional studies have yet identified the sequence or structural features that are specifically important for antitermination in the *eut* system or any other system that utilizes ANTAR-based regulatory proteins.

Using the *eut* operon from *E. faecalis* as a model system, we present evidence that a novel RNA motif comprises a specific antiterminator structure containing the full determinants for recognition by the EutV ANTAR domain. Importantly, the same RNA motif could be identified for the other ANTAR-based regulatory systems that have been studied (AmiR and NasR), suggesting that it is likely to constitute the general recognition element of ANTAR-based regulatory proteins. This structure consists of a pair of small stem-loops, one of which contains the previously identified 13-nucleotide sequence described above. Recognition of RNA by EutV relies on a combination of structure and primary sequence determinants. Specifically, certain residues within the hexanucleotide terminal loops share primary sequence conservation, particularly at the first and fourth positions, and are important for binding. We also discovered that the minimum RNA-binding module of EutV is composed of a dimer of the ANTAR domain, and that dimerization is stimulated in a signal-responsive manner. Moreover, conditions that mimic phosphorylation improved RNA-binding activity of EutV, suggesting that signal-induced dimerization is likely to stimulate RNA-binding activity. Therefore, in aggregate, these data suggest that RNA-binding response regulator proteins are likely to generally rely upon protein dimerization and recognition of tandem nucleic acid substrates, which are mechanistic features that conceptually resemble regulation by many DNA-binding factors. Finally, to assess whether the dual hairpin RNA structure might be present in other bacteria, we employed a bioinformatics-based search for this element across many bacterial genomes. These searches led to the discovery of many new regulons that are likely to be coordinated by ANTAR recognition elements. These searches also revealed that the ANTAR recognition elements described herein are generally involved in coordinating expression of nitrogen metabolism genes. In aggregate, these data reveal that ANTAR-based genetic circuits are widespread in bacteria and broadly share certain conserved molecular features.

## Results

### *E. faecalis eut* locus is regulated by ethanolamine, AdoCbl, EutVW, and transcriptional terminators

In our previous work using a *lacZ* reporter translationally fused to the 5′ leader region of the first gene of the *eut* operon (*eutP*), we observed that rich medium containing serum modestly induced expression and this induction did not occur in a *eutVW* in-frame deletion mutant [17]. Another group found that *E. faecalis* could grow anaerobically in minimal medium with ethanolamine as the sole source of carbon, as long as AdoCbl was also provided; however, a *eutVW* mutant was unable to grow under these conditions [15]. For this study we modified the minimal medium by adding ribose, a carbon source unlikely to cause catabolic repression but allowing for the growth of a *eutVW* mutant. As shown in Figure 2B, the medium worked well, and we observed a large induction of *eutP-lacZ* that was dependent on ethanolamine, AdoCbl, EutV and EutW. Importantly, all strains, including the *eutVW* deletion, grew equally well (data not shown). We constructed and tested individual mutants of *eutV* and *eutW* (in-frame deletions) and observed the same lack of induction in medium containing ethanolamine and AdoCbl (data not shown). We additionally constructed a *eutS-lacZ* reporter and also observed induced expression dependent on ethanolamine, AdoCbl and EutVW (Figure 2C).

**Figure 3:**
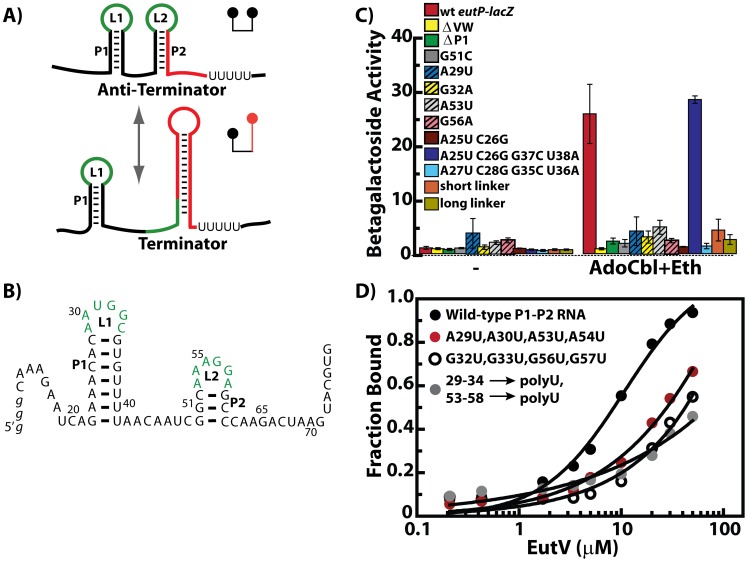
The EutV ANTAR regulator specifically recognizes a dual hairpin RNA motif. A) Alternate structures formed by the conserved RNA motif are shown. L1 and L2 denote the two terminal loops identified herein and predicted to be involved in ANTAR recognition (green). Altered base pairing allows formation of an alternate RNA structure that includes a terminator stem-loop (red). B) The primary sequence and secondary structure is shown for the dual hairpin RNA motif from the *eutP* 5′ leader region. Numbering reflects the transcription start site as +1. C) *In vivo* analysis of RNA mutants using *lacZ* reporter fusions to the *eutP* 5′ leader region is summarized as bar graphs. Deletion of *eutVW* (yellow) abolished induction of *lacZ* as compared to the wt (red). Deletion of the P1 stem-loop (green) or a mutation in the P2 stem-loop (grey) also negatively affected *eutP* inducibility. Mutation of the first (adenine) or fourth (guanine) nucleotide within the hexanucleotide loops L1 (blue stripes, yellow stripes) and L2 (grey stripes, pink stripes) significantly decreased *eutP* induction. Mutations affecting the P1 stem structure (brown) decreased induction but induction could be regained with compensatory mutations that restored the stem structure (blue). Mutations in the sequence of the closing base-pairs of stem-loop P1 abolished induction, even while maintaining the secondary structure (light blue). An increase or decrease in the length of the linker separating P1 and P2 (orange, light green) negatively affected induction. D) Binding isotherms derived from electrophoretic mobility shift assays (EMSA) are shown. Fractional saturation is plotted against protein concentration. EutV (unphosphorylated) bound the *eutP* 5′ leader region with an apparent *K*_D_ of 10 µM (black). Binding was significantly deceased in an RNA mutant where the hexanucleotide terminal loops were mutated to uridines (grey). Binding was significantly weaker with RNAs mutated in the first (red) and fourth (open circle) positions of the terminal loops. See also Figure S1 for more information on the seed alignment that was used to derive the predicted RNA secondary structure and Figure S4 for information on EutV purification.

In total, the regions immediately upstream of *eutP*, *eutG*, *eutS*, and *eutA* are predicted to contain intrinsic terminators consisting of a stem-loop followed by a run of uridines (Figure 2A, Figure 3B). Deletion of these terminator elements from the *eutP-lacZ* and *eutS-lacZ* constructs resulted in high levels of unregulated expression (Figure 2B and 2C). These data, along with a recent investigation of the transcriptional terminator in the region upstream of *eutG* [18], validate a model in which the *eut* locus is regulated in part by a series of intrinsic transcriptional terminators interspersed throughout the operon. These terminator elements are postulated to keep gene expression off under non-inducing conditions. To increase downstream gene expression under inducing conditions, the model predicts that activated EutV prevents formation of these terminators.

### The ANTAR domain specifically recognizes a dual hairpin RNA motif

EutV consists of two domains, a phospho-accepting receiver domain and an RNA-binding ANTAR domain (PF03861); however at the onset of our studies, the RNA determinants for protein recognition had not been identified. To identify features that dictate recognition by EutV, we analyzed the sequences directly upstream of *eutP*, *eutG*, *eutS* and *eutA* using RNA-fold prediction programs, M-fold (http://mfold.bioinfo.rpi.edu/) and RNAfold (http://rna.tbi.univie.ac.at/cgi-bin/RNAfold.cgi). A mini-hairpin with a short paired stem and a hexanucleotide loop was predicted upstream of the terminator in each of the four candidates (Figure 3A, 3B; Figure S1). Interestingly, we found that the RNA substrates of the two previously characterized ANTARs, NasR and AmiR, also have a short paired stem and a hexanucleotide loop upstream of the intrinsic terminator site (Figure S1). Sequences in the hexanucleotide terminal loop were previously shown to be important for NasR binding. Specifically, an A at the first position and a G at the fourth position in the loop were found to be important for transcription attenuation *in vivo* [9]. The mini-hairpins of the *eut* sequences also have an A and a G at these positions (Figure 3B and Figure S1). However, upon further inspection of these respective RNA sequences, we realized that the conserved ANTAR recognition sequences at the 5′ base of the terminators can form a second mini-hairpin, also having a hexamer loop with an A and a G at the first and fourth positions. This is true of the *eut* sequences (Figure 3B) and also for the AmiR substrate, (the leader region of the *amiE* gene) and NasR RNA substrate (the leader region of *nasF*) (Figure S1). These observations provide the basis for a general model of antitermination by members of the ANTAR family. We predict that in the unbound state, the leader RNAs of the ANTAR substrates fold to form a terminator structure. Upon activation, ANTAR-containing proteins bind the RNA, specifically interacting with the two terminal loops to stabilize an antiterminator structure and exclude terminator formation (Figure 3A).

To test the predicted importance of these structural features, site-directed mutations were introduced in the *eutP*-*lacZ* construct (Figure 3C). Deletion of the first stem loop (P1) ablated induction of *eutP* by EutV. Similarly, a single nucleotide change (G51C) within the base-paired region of the second hairpin (P2), which is predicted to prevent its formation, also reduced the efficacy of *in vivo* activation by EutV. Furthermore, we tested constructs containing mutations in the two residues that appeared to be most conserved - positions 1 and 4 within the terminal loops; alteration of any one of these four residues (A29U or G32A in L1 and A53U or G56A in L2) also resulted in reduced induction of the *eutP-lacZ* constructs (Figure 3B–3C). To test the contribution of the stem of the putative P1 hairpin, two bases on the left side of the P1 stem were altered to disrupt base pairing (A25U and C26G). This mutant construct was also no longer inducible. However, when the corresponding residues on the right side of the stem were additionally mutated to restore pairing (A25U, C26G, G37C and U38A), induction was restored. These data suggest that the contribution of these stem residues to recognition by EutV is likely to be structural rather than sequence specific. In contrast, mutation of the two closing base pairs of the stem in a manner that changed the sequence but retained the ability to form base pairs (A27U, C28G, G35C and U36A) disrupted induction. Interestingly, manual inspection of the *eut* genes from *E. faecalis*, *Listeria* and *Clostridium* species revealed primary sequence conservation of these closing base pairs (Figure S1). Based on these results we speculate that EutV is likely to associate with the terminal loops as well as the top base pair of the associated helical structures. We also assessed the general requirement for the linker region that separates the two stems. Among the *E. faecalis eut* genes, the linker varies between 5 and 12 nt. The linker in the *eutP-lacZ* construct was either reduced to 3 bases (short linker) or elongated to 14 bases (long linker). Both of these changes caused drastic reductions in induction, suggesting that an optimal proximity of the two stem-loops is required for successful interaction with EutV.

The capability of EutV to bind to the wild-type and mutant versions of the *eutP* 5′ leader region was then tested directly *in vitro* via an electrophoretic mobility-shift assay (EMSA) using 5′ radiolabeled RNA and purified protein (Figure 3D). These data demonstrate that full-length EutV associates with the wild-type dual stem RNA substrate with an apparent *K*_D_ of 10 µM. Replacement of the hexanucleotide terminal loops with an oligouridine tract greatly reduced EutV binding as did individual mutations in the conserved 1 and 4 positions within each loop (A29 and G32 of P1 or A53 and G56 of P2). Therefore, together, these data demonstrate that the tandem hairpins are important recognition elements for regulation by EutV.

### Defining the minimum ANTAR domain required for RNA recognition

**Figure 4:**
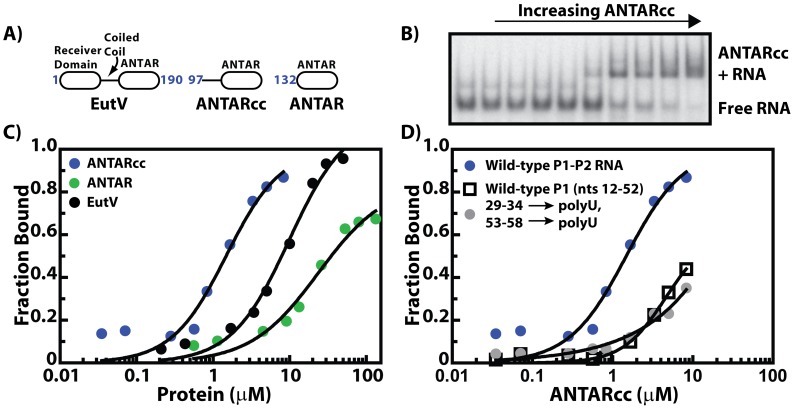
The minimum ANTAR domain for RNA recognition. A) Three variants of EutV protein were investigated: (1) full length, which included the N-terminal receiver domain, a coiled coil region and the C-terminal ANTAR domain, (2) ANTARcc, which included the coiled coil region and the ANTAR domain, and (3) just the C-terminal ANTAR domain. B) A representative EMSA is shown for association of ANTARcc with 5′-radiolabeled RNA (*eutP* 5′ leader region) on a non-denaturing polyacrylamide gel. C) Binding isotherms derived from EMSAs showing fractional saturation versus protein concentration. These data suggested that the two-stem RNA motif is bound by ANTARcc (blue), full length EutV (black), and ANTAR (green) with decreasing affinity, respectively. D) ANTARcc binds different RNA constructs with variable affinities. While RNA that included the dual hairpin motif (blue) was bound with micromolar affinity, mutation of the terminal loop sequences (grey) as well as deletion of the second stem loop (squares) abolished binding. The sequences for the constructs are shown below.

*E. faecalis* EutV is predicted to possess two domains - an N-terminal phospho-accepting receiver domain and a C-terminal ANTAR domain (Figure 4A). A region separating the two domains forms a coiled-coil as suggested by the COILS [19] server and by structural studies on AmiR as well as Rv1626, orthologs of EutV from *Pseudomonas aeruginosa* and *Mycobacterium tuberculosis* [5], [20]. The AmiR structure reveals an intimate dimer with an extended coiled-coil region, although the importance of the coiled coil region for the function of AmiR has not been studied. ANTAR itself is a poorly understood protein domain and little is known about its RNA-binding properties. Having identified the RNA target of the EutV ANTAR domain, we then investigated the protein domain requirements for RNA recognition. We expressed and purified two variants of the EutV ANTAR domain, which both lacked the response regulator receiver domain. One variant is referred herein as ANTARcc (which includes the putative coiled coil region) while the other variant is called ANTAR (which lacks the coiled coil region) (Figure 4A). Via EMSA experimentation using 5′ radioactively labeled RNA substrate and purified protein we determined the binding affinities of different recombinant proteins (Figure 4B–4C). ANTARcc binds the dual hairpin RNA substrate with an apparent affinity of ∼700 nM, a value that is 100-fold tighter as compared to ANTAR alone. This data suggests that the coiled-coil region plays an important structural role in EutV-RNA interactions. Also, as described earlier, full-length EutV in its unphosphorylated state binds RNA with an affinity of 10 µM (Figure 3D, Figure 4C), 10-fold weaker than ANTARcc. This suggests that in the unphosphorylated state, the receiver domain of EutV may damper RNA-binding activity of the ANTARcc domain. Phosphorylation of the receiver domain is likely to be accompanied by structural reorganization, perhaps allowing the ANTARcc domain to adopt a conformation better suited for RNA-binding.

Having determined that the ANTARcc protein binds the RNA target with the highest affinity, we tested its ability to discriminate between different variants of the *eutP* 5′ leader region. The presence of both of the stem-loops exhibited a significantly better affinity as compared to a single stem-loop element (Figure 4D), suggesting that both stem-loops are required for full association of the ANTARcc protein. Similarly, as observed for full-length EutV (Figure 3D), mutagenesis of the terminal loop residues deleteriously affected association with ANTARcc. In total, these data further support our premise that ANTAR domains, potentially including the coiled coil region, promote antitermination by recognizing and binding to the terminal loop residues of a dual hairpin motif.

### Phosphorylation-induced protein dimerization plays an important role in ANTAR domain interactions with the substrate RNA

**Figure 5:**
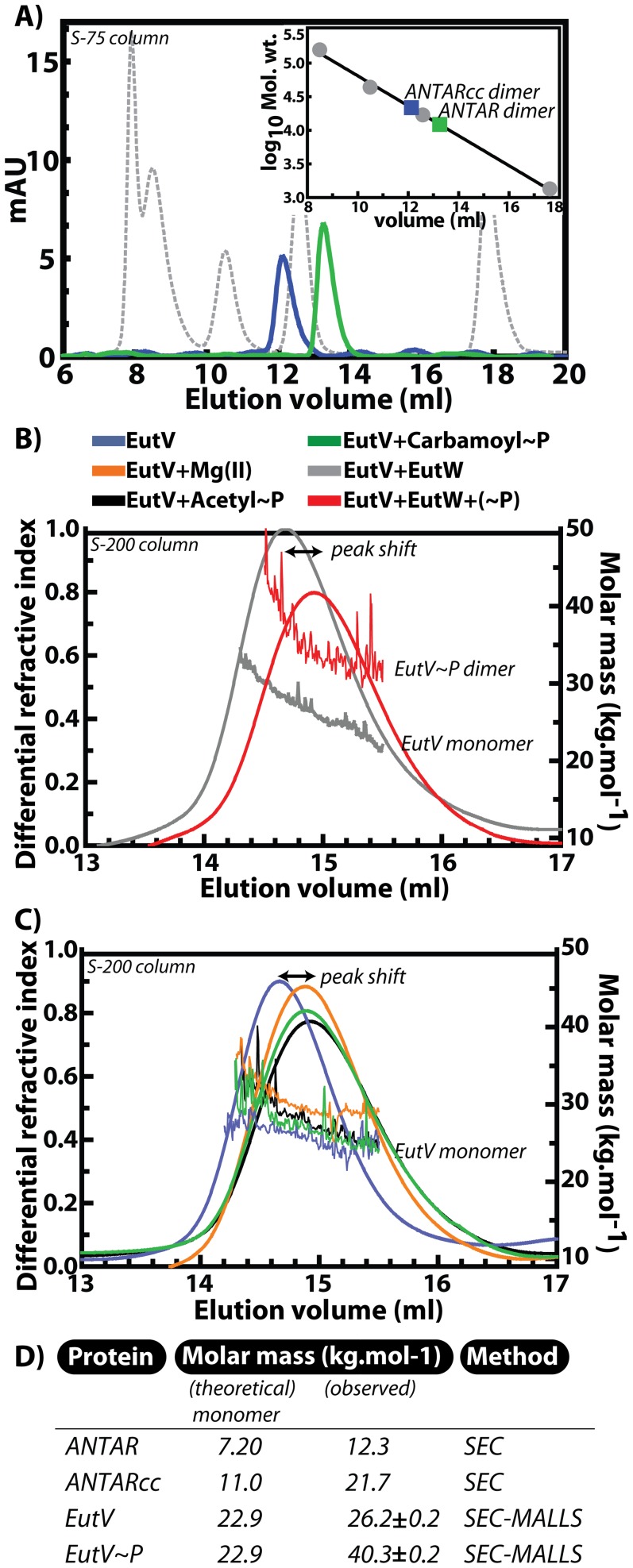
Dimerization of ANTAR-containing proteins. A) Comparison of size exclusion chromatography profiles (Superdex-75 column) for ANTARcc (blue), ANTAR (green) and protein calibration standards (grey) suggests a dimeric state for ANTARcc and ANTAR. The standard plot is shown as inset. B) Multi–angle laser light scattering (MALLS) data collected in tandem with size exclusion chromatography on a Superdex-200 column. MALLS analysis indicated that incubation of EutV with EutW in the absence of phosphorylating conditions (grey) did not result in a change in molar mass, whereas there was an altered elution time (marked as peak shift) as well as a change in molar mass from monomer to dimer upon incubation of EutV with EutW under phosphorylating conditions (red). C) A moderate change in elution volume (peak shift) occurred for EutV monomer (blue line) upon incubation with magnesium (orange), although this altered migration was not accompanied by an increase in the molar mass. Similarly, incubation of EutV with small-molecule phosphodonors acetyl phosphate (black) and carbamoyl phosphate (green) did not alter the monomeric state of EutV, beyond the shift in the peaks already attributed to magnesium addition. D) The theoretical molar masses for monomers of EutV protein variants are listed alongside experimentally observed values. SEC refers to size-exclusion chromatography and SEC-MALLS denotes size exclusion chromatography when coupled in tandem to detection by multi-angle laser light scattering.

Bacterial response regulators often display the ability to form dimers or higher oligomers [12]. We speculated that in order to bind an RNA target that presents two similar surfaces for interaction, the protein component is also likely to form a dimer or higher ordered oligomeric state to recognize the RNA substrate. To test this, we first investigated the oligomeric state of the EutV ANTAR and ANTARcc domains using size-exclusion chromatography (SEC) (Figure 5A). While SEC is limited in the precise calculation of molar masses, the low extinction coefficients of these domains at 280 nm prevented the use of preferred techniques such as equilibrium analytical ultracentrifugation. From SEC, we inferred that both the ANTAR and ANTARcc domains formed dimers when compared to the elution profiles of the standard protein markers. Therefore, dimer formation appears to be an inherent characteristic of this domain, however, as discussed above and shown in Figure 4C, the presence of the coiled coil significantly improved the affinity for RNA-binding. This suggests that although both versions of the ANTAR domain are able to form dimers, there are likely to be differences between their dimeric conformations, that are crucial for RNA recognition.

After determining the oligomeric state of the isolated ANTAR domains, we investigated the oligomeric state of full length EutV. Since we were unable to quantitatively resolve EutV oligomeric states by SEC alone, we employed a sensitive method where SEC is coupled in tandem with Multi-Angle Laser Light Scattering (MALLS). As fractions elute from a gel filtration column, which separates proteins based on size and shape, they are passed through a light scattering device. The latter conducts measurements of the differential refractive index of the various macromolecules as they elute from the column. MALLS is independent of the shape of the molecule, thereby allowing precise calculation of the molar mass for all the fractionated species. Analysis using MALLS after fractionation on a Superdex-200 column is shown in Figure 5B and 5C. These data revealed that EutV in its native state forms a monomer of approximately 22.9 KDa. We then added the cognate sensor kinase, EutW, which had previously been shown to phosphorylate EutV in the presence of ethanolamine and ATP [15], [17]. The presence of EutW alone did not induce dimerization. However, when ethanolamine, ATP and magnesium were supplied in order to induce phosphorylation, EutV's molar mass approximately doubled, indicating that dimerization was induced by phosphorylation (Figure 5B).

**Figure 6:**
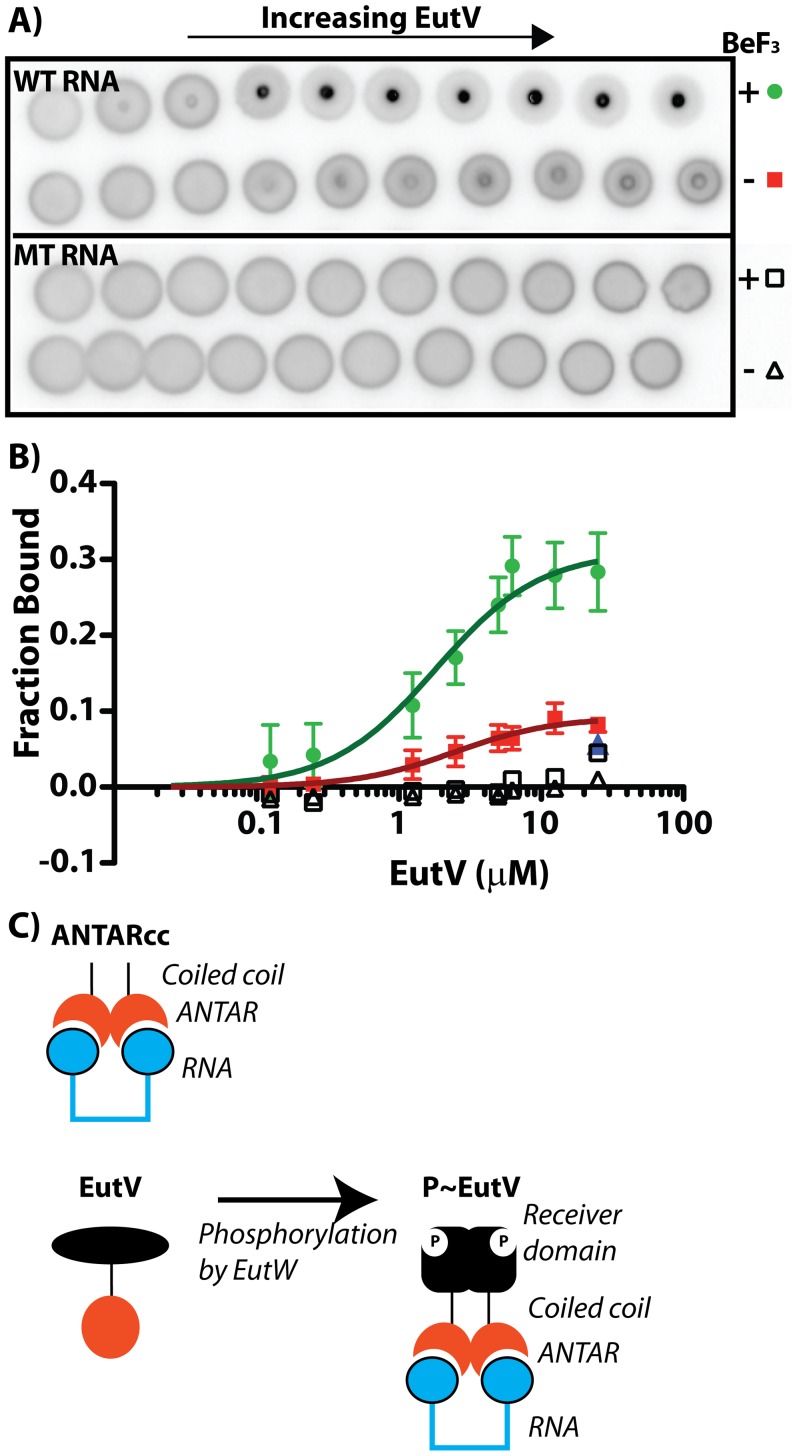
Phosphorylation of EutV correlates with improved RNA-binding affinity. Beryllium fluoride was added to EutV to mimic phosphorylated protein. To measure binding activity using an experimental method that was unaffected by beryllium fluoride we employed the differential radial capillary action of ligand assay (DRaCALA) [25]–[26]. A) For this experiment, a 5′-radiolabeled RNA was incubated with increasing amounts of protein and spotted onto a nitrocellulose membrane. Proteins bound to ligands are sequestered to the membrane at an inner spot, whereas all unbound ligand is able to radially diffuse away from the spot by capillary action. Using this assay, the RNA-binding activity of EutV was tested for the wild-type dual hairpin RNA or a mutant RNA containing oligo-uridines in the terminal loops. These reactions were conducted in the presence and absence of beryllium fluoride. B) DRaCALA data was plotted as the fraction bound versus EutV concentration. EutV in the presence of beryllium fluoride binds the wild-type dual hairpin RNA (green) with a higher affinity than in the absence of beryllium fluoride (red). Competition with unlabeled wild-type RNA (blue) at 10-fold excess reduced the fraction of bound RNA to the level seen in the absence of beryllium fluoride. EutV does not bind the mutant RNA in either the presence (open square) or absence (open triangle) of beryllium fluoride. C) A general model for the binding of RNA by EutV is presented herein, which is based on the aggregate data and is discussed in the text.

Many response regulators are capable of autophosphorylation in the presence of small molecule phospho-donors such as acetyl phosphate, carbamoyl phosphate, or phosphoramidate. We tested the two most common small-molecule phosphodonors (acetyl phosphate and carbamoyl phosphate) for their ability to induce dimer formation. Although EutV did not form dimers in response to addition of these small molecules, they appeared to provoke a moderate conformational change in EutV, visualized as a delay in the elution volume. However, further tests revealed that magnesium alone was responsible for promoting the moderate conformational change in EutV as it had also been included with the small molecule phosphodonor solutions (Figure 5C). Since the SEC-MALLS experiments suggested that small molecule phosphodonors were unable to promote dimerization we reasoned that they could not be used as tools for probing the effects of phosphorylation-induced dimerization on RNA-binding activity. For many response regulators, the half-lives of the phosphorylated receiver domains can be very short due to the intrinsically labile aspartyl phosphate bond [21]–[22]. As an alternative, we added beryllium fluoride as a nonhydrolyzable mimic of phospho-aspartate [23]–[24] and measured EutV RNA-binding activity. Preliminary experiments with addition of beryllium fluoride to EutV revealed that the beryllofluoride addition negatively affected resolution of the EutV-RNA complexes in the EMSA assay format (data not shown). Therefore, a recently developed non-electrophoresis method called differential radial capillary action of ligand assay (DRaCALA) was instead employed for these purposes [25]–[26]. DRaCALA is a rapid and quantitative assay for protein-ligand interactions that is based on the ability of nitrocellulose membranes to preferentially sequester proteins over small molecule or nucleic acid ligands. Specifically, proteins and their radiolabeled ligands are immobilized together when spotted onto nitrocellulose membranes, while unbound radiolabeled ligands freely diffuse by capillary action away from the protein spot. The fraction of the targeted protein bound with its mobile ligand can be easily calculated using this assay, which has been validated in recent publications for proteins that bind small molecules [25] and nucleic acids [26]. Using DRaCALA, we radiolabeled the two hairpin RNA motif and quantified binding to EutV in the presence or absence of beryllium fluoride (Figure 6A–6B). The binding affinity of unphoshorylated EutV for the two hairpin RNA motif as measured by DRaCALA was similar to that seen previously by EMSA, further validating the use of this method. Addition of beryllium fluoride provoked a significant increase in RNA-binding activity for wild-type RNA but not for a negative control RNA containing mutations in the terminal loops. Moreover, addition of cold competitor RNA restored the apparent fraction bound to background levels.

From these combined results we propose a general model for EutV regulation (Figure 6C). Protein variants consisting only of the ANTAR and coiled-coil region can form dimers alone (Figure 5A), whereas full-length unphosphorylated EutV protein remains a monomer (Figure 5B). Therefore, the unphosphorylated receiver domain is likely to prevent EutV dimerization, possibly by steric hindrance, and only upon signal-induced phosphorylation does the full-length EutV protein dimerize (Figure 5B) and bind with highest affinity to the target RNA (Figure 6A–6B). Indeed, ANTARcc, which forms stable homodimers, exhibits an RNA-binding affinity similar to that of phosphorylated EutV and is significantly improved relative to unphosphorylated EutV (Figure 4C). Therefore, signal-induced dimerization of ANTAR proteins is likely to be essential for recognition of symmetric nucleic acid ligands, which is conceptually similar to the molecular mechanism exhibited by many DNA-binding response regulator proteins [27].

### Bioinformatics searches reveal the widespread distribution of ANTAR RNA substrates

**Figure 7:**
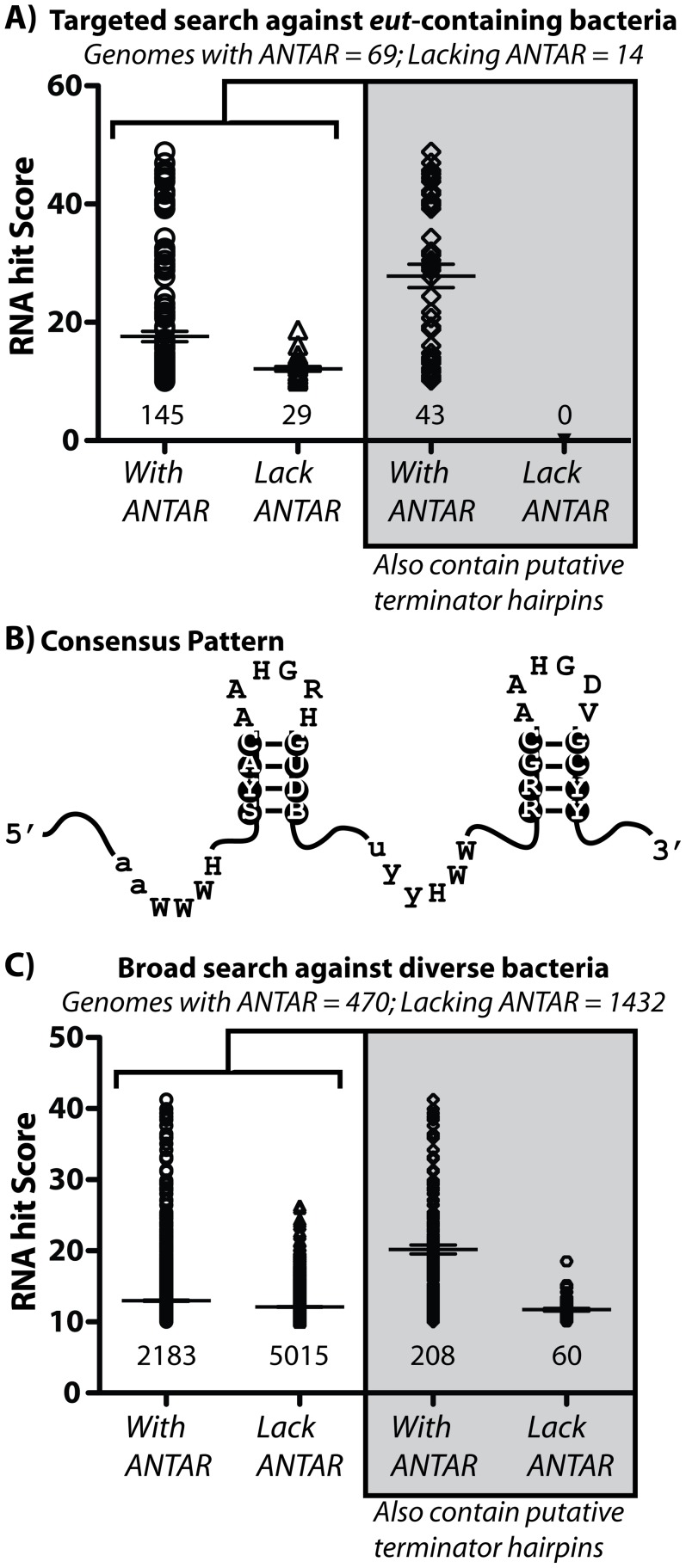
Bioinformatic analysis of the ANTAR domain and its two stem-loop RNA substrate. A) Using a covariance-based search approach (Infernal [28]), we identified additional occurrences of the ANTAR RNA substrate in bacteria that contained *eut* pathways. The comparative sequence alignment of these RNA sequences is shown in Figure S2 and described in Table S1. A scatter plot is shown for the resulting RNA hits, where each data point represents a different RNA hit. The hit scores for these sequences were plotted for two classes of microorganisms used in this search. Specifically, organisms that are predicted to encode for ANTAR domain proteins (see Table S1) have more RNA hits with higher scores than a control set of organisms that appear to lack any ANTAR domain proteins. Also, these hits were screened using TransTerm for the presence of an intrinsic terminator hairpin located immediately downstream of the P2 helix. Only a subset of the hits satisfied this important criterion. B) A consensus secondary structure was derived from this sequence alignment and is shown herein. C) The covariance-based search approach was then employed against 1902 bacterial genomes to search more broadly for putative ANTAR-based regulatory pathways. Again, a scatter plot is shown for the resulting hits, and for the subsequent screening of these hits for the presence of an overlapping downstream intrinsic terminator hairpin. See also Figures S1, S2 and Tables S1, S2 for more information on the covariance search results.

Given the close sequence and structural similarity between the dual hairpin RNA motif in the three different characterized ANTAR systems (AmiR, NasR, EutV), we hypothesized that the RNA motif as identified herein might be generally representative of ANTAR substrates in other organisms. Also, the three previously characterized ANTAR regulatory systems each affected a single locus in their respective host organisms, and we reasoned that a subset of bacteria might instead incorporate multiple ANTAR-responsive RNA elements at disparate genomic locations for coordination of ANTAR-based regulons. To this end, we searched for additional occurrences of the putative ANTAR RNA substrate using a bioinformatics-based approach. Specifically, we used a covariance model-based approach [28] wherein a basic sequence alignment of a target RNA element, including certain secondary and primary sequence determinants, is used as input criteria for discovery of additional representatives from fully sequenced bacterial genomes. This method has been successfully employed for larger, structured RNAs such as riboswitches, and is also the underlying algorithm currently used by the Rfam database team to curate bacterial noncoding RNAs [29]. Therefore, a seed alignment was created based on the putative ANTAR RNA substrates (the dual hairpin element) from the *eut* loci of *E. faecalis*, *Clostridium* and *Listeria* species, as well as the corresponding RNA sequences for *Klebsiella oxytoca nasF* and *Pseudomonas aeruginosa amiE*, which are the target substrates for NasR and AmiR, respectively (Figure S1). This RNA element was defined as a dual hairpin motif with a minimum of three base-pairs in each stem and a variable linker region connecting the two stems. Sequence conservation in the loops, with an adenine at position 1 and a guanine at position 4 of each loop was maintained. Given the relatively small size of the motif and the small number of residues conserved at the primary sequence level, the first search was targeted against a narrowly defined genomic subset. We reasoned that this would allow us to fully examine the quality of our individual RNA hits. For this target analysis we searched against 83 bacterial genomes that were previously predicted [30] to specifically encode for a putative *eut* locus. Some *eut* loci are regulated by a DNA-binding regulator called EutR (*e.g.*, *Salmonella*, *Escherichia*) whereas others, especially the Firmicutes, are regulated by a RNA-binding, ANTAR-containing homolog of EutV, as in *E. faecalis* [30]. Therefore, a subset of these genomes contains putative *eut* pathway homologues but lack any ANTAR-encoding genes, while other genomes contain both. As predicted, we recovered less RNA hits in genomes that lack ANTAR-encoding genes (Figure 7A). Another strength of the subset of genomes chosen for the initial analysis is that they include phylogenetically diverse species representative of many different evolutionary lineages.

This covariance-based search revealed the presence of many putative ANTAR RNA targets (Figure S2; Table S1). Our approach was validated in part by the identification of all 17 input sequences that were used to derive the seed alignment. Most hits (>83%) originated from bacteria that encoded for at least one ANTAR-encoding gene (Figure 7A; Table S1). Moreover, the average “hit score” was higher for RNA hits from organisms that encoded for at least one ANTAR gene (Figure 7A), suggesting that the RNA element is at least partially correlative with the presence of ANTAR-containing genes. These newly identified putative ANTAR substrates originated from diverse bacteria, including Gram-positive bacteria (*e.g.*, *Mycobacterium*, *Streptococcus*, *Fusobacterium*, *Alkaliphilus*, etc.) and Gram-negative bacteria (*e.g.*, *Pseudomonas*, *Burkholderia*, etc.), and resulted in a consensus pattern that resembled the input consensus pattern (Figure 7B). The ANTAR systems that have been previously characterized are each used to regulate transcription attenuation. To examine whether some or all of the hits acquired in this analysis are also likely to mediate transcription attenuation we screened them using TransTerm for candidate intrinsic transcription terminator hairpins that overlapped with the P2 helix. Approximately 30% of the hits satisfied this criterion for organisms that encoded for ANTAR genes, whereas none of the RNA hits satisfied this criterion from organisms lacking an ANTAR gene (Figure 7A). Moreover, the average hit score increased further for the hits that contained terminator hairpins. Therefore, these putative hits represent the best possible candidates for new ANTAR-based regulatory systems. However, it is important to note that many of the remaining hits (lacking terminator hairpins) may still function as actual ANTAR regulatory elements, but via regulatory strategies other than transcription attenuation, such as control of translation initiation. Indeed, manual inspection of some of these hits revealed instances where they were arranged near to, or overlapping with the ribosome binding site of the downstream gene (*e.g.*, Figure S3).

**Figure 8:**
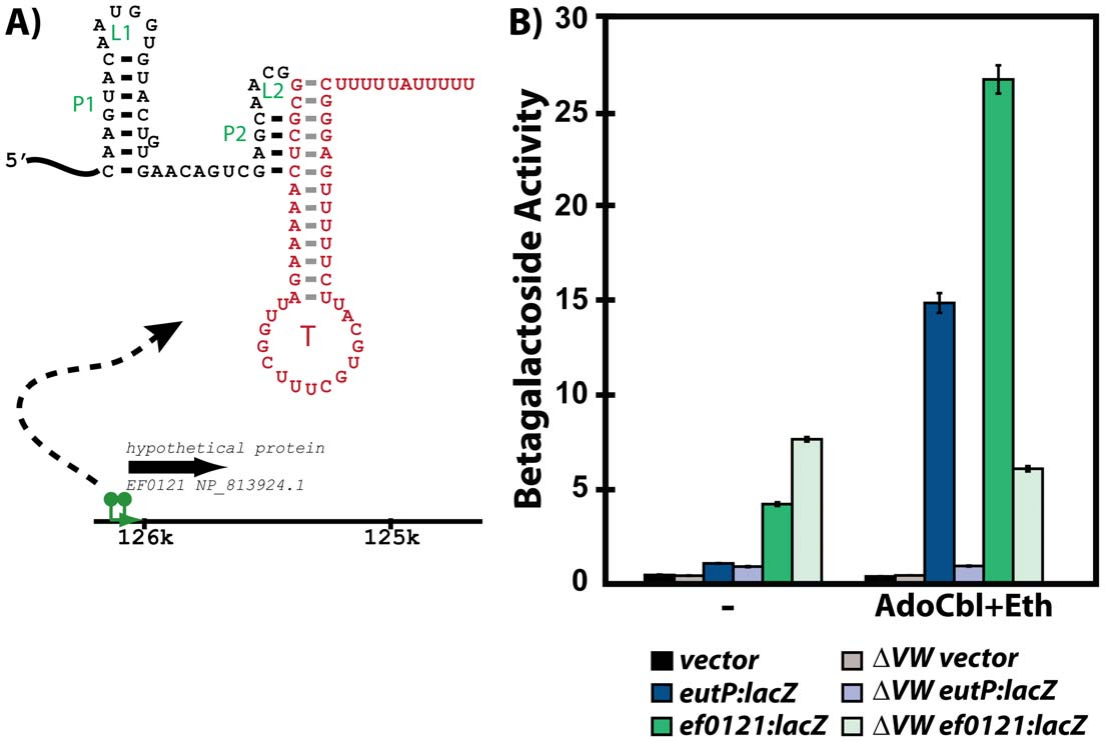
Responsiveness of the *ef0120* leader region to regulation by EutVW. A) One outcome of the covariance searches was identification of a new putative ANTAR hit within the *E. faecalis* genome. This hit contained an overlapping intrinsic terminator hairpin, as identified by TransTermHP, despite the fact that a terminator was not a stringent requirement of the initial search criteria. B) To test whether this hit was functionally responsive to EutVW *in vivo*, the leader region of this gene was translationally fused to a *lacZ* reporter and monitored with and without AdoCbl and ethanolamine.

**Figure 9:**
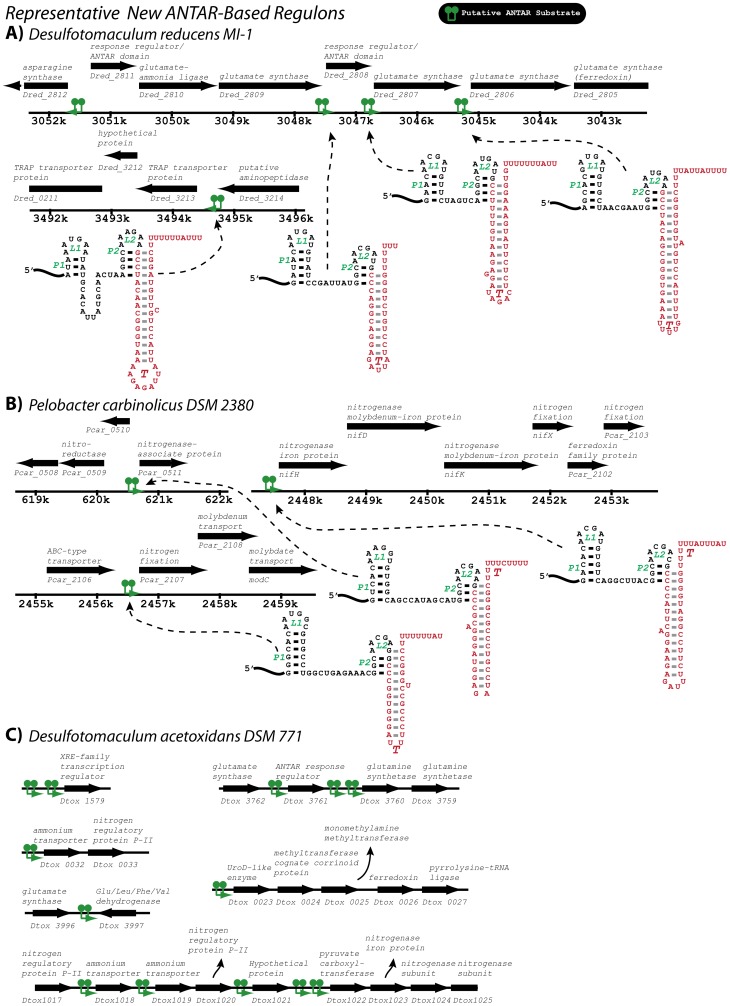
A few representative ANTAR-based regulons identified in this study. RNA hits (green) from the covariance searches (Tables S1, S2; Figure S2) are shown within their genomic contexts for two representative organisms. Genes are shown along with their annotations (black). The putative ANTAR substrate RNAs appear to be present in multiple operons in the same bacterium, and are thereby likely to participate in control of ANTAR-based regulons. In these examples, the regulons are predicted to be functionally related to control of glutamate metabolism and nitrogenase expression, respectively. See also Figures S1, S2 and Table S1 for more information on the covariance search results. To highlight the sometimes extensive utilization of ANTAR target RNA motifs for certain organisms, the newly identified putative ANTAR-based regulon is shown for *Desulfotomaculum acetoxidans*. Based on our search this organism utilizes at least 13 ANTAR-based transcription attenuation systems, affecting a total of six transcriptional units that are involved in various aspects of nitrogen metabolism.

Interestingly, while most of the new hits in this search were associated with *eut* genes, many were not. For example, a new ANTAR substrate was unexpectedly identified in the *E. faecalis* genome outside of the *eut* locus and within the 5′ leader region of *ef0120*, suggesting that EutVW might indeed control a regulon rather than a single locus. To investigate this observation further we fused the *ef0120* 5′ leader region to *lacZ* and monitored expression in the presence and absence of AdoCbl, ethanolamine, and the EutVW genes (Figure 8). Indeed, expression was activated by AdoCbl and ethanolamine in a EutVW-dependent manner. Therefore, our covariance search of putative ANTAR substrates is likely to have revealed ANTAR-based coordination of *E. faecalis* genes from both inside and outside of the *eut* locus. In fact, this search revealed many examples where putative ANTAR substrates were associated with multiple functionally related operons, as one might expect for regulons. For example, new ANTAR substrates appeared to be co-transcriptionally linked to different glutamate synthase genes in the *Desulfotomaculum reducens* genome (Figure 9A). Similarly, in *Mycobacterium vanbaalenii*, a putative ANTAR RNA substrate is positioned upstream of multiple uncharacterized gene clusters unrelated to *eut* genes (data not shown). In *Pelobacter carbinolicus* new ANTAR substrates were located within three separate transcriptional units, which are each predicted to be important for nitrogenase function, suggesting a comprehensive ANTAR-based regulon for nitrogenase regulation in this microorganism (Figure 9B).

To broaden this search outside of organisms containing *eut* pathways, we repeated the covariance search with moderately more restrictive criteria against 1902 bacterial genomes, of which 470 included homologues for ANTAR-containing genes (Figure 7C). After screening these hits for the presence of an intrinsic transcription terminator overlapping the P2 helix the average hit score was decidedly increased for organisms encoding an ANTAR gene as compared to organisms lacking ANTAR genes. This search revealed many more examples of excellent candidates for ANTAR-mediated regulons in diverse bacterial species, including Gram-negative and Gram-positive bacteria (Table S2). Remarkably, the majority of these hits were consistently located upstream of nitrogen metabolism genes. Indeed, both searches revealed a close association between nitrogen metabolism genes and ANTAR-based regulation. For example, RNA hits were discovered upstream of genes included but not limited to: nitrogen regulatory protein P-II, ammonium transporters, urea transporters, nitrate and nitrite transporters, nitrite reductase, nitrogenase subunits, and synthase enzymes for glutamate, glutamine, and arginine. This point is particularly illustrated by *Desulflotomaculum acetoxidans*, which employs at least 13 new regulatory RNA hits within six transcriptional units that encompass many of these metabolic functions (Figure 9C). It is worth noting that the previously studied *eut*, *nas* and *ami* operons encode for ethanolamine catabolism, nitrate assimilation and ammonia-releasing amidases, respectively, which are also all tied to nitrogen metabolism Therefore, the aggregate data presented herein reveal clearly that ANTAR-based regulons are widely used in bacteria for control of nitrogen metabolism genes.

## Discussion

The ANTAR protein domain appears to be a global regulatory domain that is used in multiple combinations with putative sensory domains. These combinations yield signal-responsive regulatory proteins that are likely to respond to a wide variety of signals, but rely on common interactions between the ANTAR domain and its RNA substrate for affecting regulation. This has been the motivation for this study, wherein we have identified a common ANTAR substrate RNA and determined some basic features that govern the ANTAR-RNA recognition. Specifically, our data in aggregate suggest that ANTAR proteins recognize a common RNA motif consisting of two tandem stem-loops. Certain primary sequence determinants within the terminal loops and at the apex of the helices appear to be conserved for these RNA elements. These features appear in the putative ANTAR substrates of both Gram-positive bacteria, such as the *eut* genes of *E. faecalis*, and Gram-negative bacteria, such as the *ami* and *nas* operons [4], [6], [8]. We speculate that our analysis has revealed the basic RNA scaffold for recognition by ANTAR protein domains, but that it is likely that there will be specificity determinants that occur in the residues that are less conserved at the primary sequence level. Moreover, we provide evidence both *in vitro* and *in vivo* that the second hairpin (P2) can alternately base pair with the downstream transcript to form an intrinsic terminator. In this way, the ANTAR target RNA appears to act as a transcriptional antiterminator that may be stabilized upon ANTAR binding. The dual hairpin motif is unique compared to the antiterminators employed by other bacterial regulators such as the Sac/Bgl and TRAP proteins. For the *trp* operon, multiple molecules of the RNA-binding TRAP protein associate with tandem UAG trinucleotide repeats in the transcript. Some of these trinucleotide repeats are located within the antiterminator sequence [3]. Therefore, binding of TRAP prevents antiterminator formation and instead promotes a terminator hairpin. The Sac and Bgl proteins recognize and bind a 29–30 nucleotide stretch called RAT (Ribonucleotide AntiTerminator) that partially overlaps with the terminator sequence [31]–[32]. This RNA-protein interaction causes the RAT region to fold into a single stem-loop structure that occludes the formation of the terminator. Thus, in most previously characterized systems, the antiterminator is a single alternative hairpin structure that overlaps with the terminator in a manner such that only one hairpin can prevail. Therefore, the broad distribution of ANTAR-containing genes inspired us to investigate whether a common substrate could be identified for the ANTAR domain in isolation, and whether there might be general mechanistic features for ANTAR-based regulation that are similar or different from previously characterized systems.

We find that the minimum ANTAR domain alone can dimerize, although the adjacent coiled-coil region is likely to alter the dimer to improve its RNA-binding ability. The functional importance of protein dimerization is further strengthened by the observation that other classes of ANTAR proteins are also likely to form dimers or higher ordered oligomers. For example, many other ANTAR proteins possess an N-terminal coiled-coil region (Pfam PF03861), suggesting a broad role for dimerization. Similarly, most other ANTAR architectures include protein subdomains that are known to dimerize, such as PAS and GAF domains. Our data also suggest that dimer formation is likely to affect the mechanism of activation by ANTAR response regulators. SEC-MALLS analysis of full length EutV indicated that it exists as a monomer in the unphosphorylated state. However, phosphoryl transfer from the cognate sensor kinase, EutW, induced EutV dimerization, suggesting that dimerization is integrated with the appropriate TCS signal. Moreover, phosporylation-induced dimerization also improves RNA-binding affinity overall. This is in agreement with previous observations by Baker and Perego where a 3-fold increase in transcriptional readthrough of an intrinsic terminator upstream of *eutG* was noted under phosphorylating conditions of EutV [18].

The ANTAR domain itself is widespread in bacteria. Therefore we reasoned that the RNA substrate that is recognized by this domain should also exhibit a high degree of conservation in the critical recognition elements. Hence we employed bioinformatic searches to identify ANTAR target RNAs in bacterial genomes. This revealed a correlation between the presence of the ANTAR domain and the dual hairpin substrate RNA. Moreover, the search for ANTAR RNA substrates led to the discovery of many new ANTAR-based regulons, which mostly appear to be part of functionally-related operons involved in various aspects of nitrogen metabolism. Why one mechanism of regulatory control would be so tightly associated with nitrogen management in unrelated bacteria is intriguing but will warrant further investigation.

Our findings represent a significant advancement in the understanding of antitermination mechanisms in general and that of the ANTAR family of regulatory proteins in particular. It provides, a common model of antitermination employed by ANTAR proteins that was validated by *in vitro*, *in vivo* and *in silico* techniques using the *E. faecalis* EutV/EutW TCS as a model. These data together elucidate the general properties of the ANTAR RNA substrate consisting of a dual hairpin element, a generally novel antiterminator structural arrangement. Now that we understand many of the critical features of this RNA element, it can be used to predict ANTAR-based operons across all sequenced bacteria, and for the future design of orthogonal ANTAR-based synthetic gene regulatory circuits.

## Methods

### Bacterial strains and media

All bacterial strains used in this study are listed in Table S3. Media was purchased from DIFCO and chemicals from Sigma, unless otherwise mentioned. *E. coli* strains were routinely cultured in Luria Bertani broth at 37°C. Antibiotics were used at the following concentrations (µg/ml): ampicillin, 100; spectinomycin, 100; erythromycin 300. *E. faecalis* strains were cultured in Brain Heart Infusion (BHI) medium at 37°C. Antibiotics were added at the following concentrations (µg/ml): erythromycin, 50; and rifampicin, 100.

### Plasmid construction

Details on plasmid construction are described in the supplementary materials (Text S1).

### β-galactosidase assays

Overnight cultures were grown in BHI broth with 50 µg/mL erythromycin. Cultures were then diluted 1∶20 into modified M9HY (MM9HY) medium or MM9HY supplemented with 33 mM ethanolamine, or 40 µg/mL AdoCbl or both. The MM9HY medium was prepared as described in Del Papa and Perego, but included 0.2% ribose. Strains were then grown anaerobically, without agitation for 3.5 hrs in the dark, and samples were collected by centrifugation. Cells were resuspended in 1∶10 Z buffer (Z buffer, 60 mM Na_2_HPO_4_/40 mM NaH_2_PO_4_/10 mM KCl/1 mM MgSO_4_/5 mM β-mercaptoethanol, pH 7.0) and lysed using 0.1 mm glass disruption beads (Research Products International) for 2 minutes in a mini bead beater. Samples were cleared by centrifugation and incubated with *o*-nitrophenyl β-D-galactoside (ONPG). Color development was measured at 414 nm using a plate reader. Total protein in samples was estimated using a Pierce BCA kit (Thermo Scientific) as per manufacturer's instructions. Reporter activity was reported in arbitrary units that indicate absorbance per µg/µl total protein. Samples were assayed in triplicate. Mean and standard deviations were calculated from experimental replicates. Each mutant was analysed on at least three independent occasions. Data shown reflects the average and standard deviations of all occasions of which a mutant was analysed.

### Protein expression and purification

The methodology for overexpression and purification of proteins in this study are described in detail in the supplementary materials (Text S1).

### RNA preparation

RNA for *in vitro* studies was prepared as described previously [33]. DNA templates were created by PCR on *E. faecalis* V583 genomic DNA using oligonucleotide primers AR064/AR065 for the *eutP* 5′ leader region including the terminator, AR064/AR066 for the dual hairpin motif only, and AR064/AR067 for single stem-loop sequences. DNA serving as template for the transcription of mutant RNAs was generated using oligonucleotide primers (AR118 and AR151) with template DNA oligos (AR150, AR152, AR153) purchased from Integrated DNA technologies. The forward primers added the promoter sequence for T7 RNA transcription. PCR products were processed using the Qiagen PCR clean-up kit. RNAs were synthesized by T7 RNA polymerase at 37°C for 2.5 hours and the reactions were terminated with 2× volume 8 M urea. Transcript products were resolved by denaturing 6% PAGE and visualized by UV shadowing. Passive elution [33] followed by ethanol precipitation was performed and the RNA was quantified using absorbance at 260nm.

### RNA–binding studies using electrophoretic mobility shift assays or DRaCALA

Different RNAs corresponding to the 5′ region of *eutP* were transcribed and radiolabeled with γ-^32^P ATP. RNA (∼2 fmol) was incubated with increasing concentrations of protein in a 10 µl reaction containing 50 mM Hepes pH 7.5, 150 mM sodium chloride,10 mM MgCl_2_ and 2.5 ng/µl yeast tRNA for 30 minutes at 25°C. Beryllium fluoride was used as a phosphor-Asp mimic, to generate phosphorylated EutV. For this, EutV at 30 µM concentration was mixed with 5 mM beryllium chloride and 15 mM sodium fluoride and allowed to equilibrate at room temperature for 30 minutes. The samples were then subjected to either EMSA or differential radial capillary action of ligand assay (DRaCALA) [25], [26]. For EMSA, 10 µl of each sample was run on non-denaturing 5% TBE polyacrylamide gels (5% acrylamide: bis (80∶1). Gels were pre-run for 30 minutes at 40 volts,electrophoresed at 40 volts for ∼2.5 hours, with 0.5× TBE running buffer and dried for 45 mins. For DRaCALA, 3 µl of each sample was spotted on a nitrocellulose membrane and allowed to air dry for 30 mins. Gels for EMSA and nitrocellulose membranes for DRaCALA were exposed overnight and visualized using a PhosphorImager (GE Health Sciences). Quantification was performed using the ImageQuant software (Molecular Dynamics) and data plotted using GraphPad Prism. The fraction of protein-ligand complexes was calculated as described previously [25]–[26].

### Multiangle laser light scattering

Size-exclusion chromatography was performed for EutV on an HR 10/300 Superdex-200 column (GE Health Sciences) and for ANTAR or ANTARcc on an HR 10/300 Superdex-75 column at a flow rate of 0.5 ml/min in running buffer (25 mM Hepes pH 7.5, 150 mM NaCl, 2 mM DTT). Runs were performed with EutV at 10 µM and EutW at 0.5 µM. Phosphorylation conditions included ATP (5 mM), MgCl_2_ (5 mM), ethanolamine (2 mM). In runs containing small-molecule phosphodonors, carbamoyl phosphate (5 mM) or acetyl phosphate (10 mM) were used. Elution profiles of differential refractive index versus volume were recorded and analyzed using the Optilab instruments and the Astra software (Wyatt Technologies Inc.). Data were plotted using the Astra software (Wyatt Technologies Inc.).

### Identification and analysis of ANTAR target RNAs

Previous work by Tsoy et al. listed 84 bacterial genomes that contain putative ethanolamine utilization genes [30]. Eighty-three of these reference genomic sequences were available at the NCBI RefSeq genome database (http://www.ncbi.nlm.nih.gov/RefSeq/) and were downloaded and combined in a searchable database. An input RNA alignment consisting of 17 ANTAR substrate sequences was manually created. These sequences represented *eut* intergenic regions from *E. faecalis*, *Clostridium* and *Listeria sp.* where we manually identified the ANTAR-binding dual hairpin motif. This motif consisted of 3 bp stem regions with hexa-nucleotide terminal loops. The distance between the two stems was variable. The alignment was used as input for Infernal [34]. Potential RNA hits identified by Infernal were scored according to the level of similarity to the consensus sequence alignment and sorted according to bit scores. An arbitrary bit score cut-off of 10 was applied in order to catalog putative ANTAR substrate hits; this value was high enough to include all of the ANTAR substrates from *Enterococcus*, *Clostridium*, and *Listeria* included in the seed alignment. This cut-off was retained for all further searches. Also, all hits located within coding regions were eliminated from any further analysis. To create a template alignment for the catalog of ANTAR substrate hits, the portions of the hits that included the stem 1 and stem 2 regions were separately subjected to the comparative sequence alignment software LocaRNA [35] and RNAalifold [36]. After some manual adjustment, these data are presented in Figure S2. This consensus pattern alignment was then used for an additional Infernal search. A catalog of NCBI annotated bacterial genomes was retrieved from ftp://ftp.ncbi.nih.gov/genomes/Bacteria/ and filtered to remove all but one genomic sequence from each available strain. Again, an arbitrary bit score cut-off of 10 was used to catalog putative ANTAR substrate hits. To further filter these hits, they were screened for the presence of putative intrinsic transcription terminator hairpins using TransTermHP. For this step, we utilized the default model with an adjustment to allow for a larger (up to 26 nucleotide) terminal terminator loop. The only hits that passed both filters were those that were arranged such that the second stem-loop (P2) overlapped with a putative intrinsic terminator that was also oriented in the correct direction. We reasoned that this subset of hits represented the most likely examples to participate in transcription attenuation mechanisms.

## Supporting Information

Figure S1 Seed alignment of ANTAR substrate hits. Previously [17], we had noted the presence of a putative conserved element (denoted by asterisks) upstream of terminator elements in the intergenic regions of *eut* pathways from *Enterococcus*, *Listeria*, and *Clostridium* species. Our subsequent inspection of these regions (described in this manuscript) revealed the presence of two tandem stem-loops (red = helices; blue = terminal loop residues). Therefore, instead of one putative conserved region (asterisks), it appeared that two conserved hairpins were located upstream of each of the *eut* terminators from these organisms. We also noted that the first and fourth positions of both terminal loops were an A an G, respectively, for all of these putative regulatory elements. These features are also present in the *K. oxytoca nasF* and *P. aeruginosa amiE* leader sequences. Therefore, these general criteria were used to generate a seed alignment for a broader search against bacterial genomes that contained *eut* pathways (Figure S2; Table S1).
(DOCX)

Figure S2 Alignment of ANTAR substrate hits from *eut-*containing bacteria. The seed alignment of the ANTAR RNA substrates from the *eut* pathways of *E faecalis* and *Listeria monocytogenes* was used to perform an Infernal-based search [28] against 83 *eut*-containing bacterial genomes. The results of this search are summarized in Table S1. Each of the hits was assigned an arbitrary hit number, which just demarcates their initial ranking in the search results. These hit numbers are included in Table S1 and in Figure S2, so that the respective data can be easily cross-referenced. In general, most hits contained two stem loops (P1 and P2), each of which exhibited a similarly conserved terminal loop sequence. A consensus pattern is shown at the top and bottom of the comparative sequence alignment. The relevant IUPAC codes for this consensus pattern are as follows: R (A or G), Y (C or U), W (A or U), H (A or C or U), S (G or C), D (A or G or U), V (A or C or G), N (G or A or C or U).
(DOCX)

Figure S3 Example of an ANTAR target RNA predicted to influence translation rather than transcription attenuation. A subset of the putative ANTAR target RNAs identified in *eut*-containing organisms overlapped with an intrinsic transcription terminator, despite the fact that inclusion of an intrinsic terminator was not part of the original search criteria (highlighted in yellow in Table S1). These ANTAR target RNAs are therefore most likely to be responsible for controlling transcription attenuation. However, it is likely that many of the remaining putative ANTAR hits are still functional genetic elements, but that they employ a regulatory mechanism other than transcription attenuation, such as controlling the efficiency of translation initiation. To highlight this possibility, this supplementary figure includes an example of a putative ANTAR target RNA that overlaps a ribosome-binding site and the translation start of the downstream gene, which in this case is a methyl-accepting chemotaxis protein. Follow-up experimentation would be required to verify translational control by this particular putative ANTAR-responsive element.
(DOCX)

Figure S4 Purification of EutV. Purification of EutV on a DEAE column following purification on TALON affinity resin. EutV elutes as two peaks (black), which differ in the ratio of absorbance 260 nm/280 nm (blue). The first peak is enriched in free EutV whereas the second peak contains EutV contaminated with nucleic acids.
(DOCX)

Table S1 Covariance Search Results for Discovery of ANTAR Substrates in *eut*-Containing Bacteria.
(DOCX)

Table S2 Covariance Search Results for Discovery of ANTAR Substrates that Overlap Terminator Hairpins in Diverse Bacteria.
(DOCX)

Table S3 Strains, plasmids and primers used in this study.
(DOCX)

Text S1 Supplementary methods.
(DOC)

## References

[1] Stulke J (2002). Control of transcription termination in bacteria by RNA-binding proteins that modulate RNA structures.. Arch Microbiol.

[2] van Tilbeurgh H, Declerck N (2001). Structural insights into the regulation of bacterial signalling proteins containing PRDs.. Curr Opin Struct Biol.

[3] Babitzke P, Gollnick P (2001). Posttranscription initiation control of tryptophan metabolism in Bacillus subtilis by the trp RNA-binding attenuation protein (TRAP), anti-TRAP, and RNA structure.. J Bacteriol.

[4] Shu CJ, Zhulin IB (2002). ANTAR: an RNA-binding domain in transcription antitermination regulatory proteins.. Trends Biochem Sci.

[5] O'Hara BP, Norman RA, Wan PT, Roe SM, Barrett TE (1999). Crystal structure and induction mechanism of AmiC-AmiR: a ligand-regulated transcription antitermination complex.. EMBO J.

[6] Wilson SA, Wachira SJ, Norman RA, Pearl LH, Drew RE (1996). Transcription antitermination regulation of the Pseudomonas aeruginosa amidase operon.. EMBO J.

[7] Norman RA, Poh CL, Pearl LH, O'Hara BP, Drew RE (2000). Steric hindrance regulation of the Pseudomonas aeruginosa amidase operon.. J Biol Chem.

[8] Chai W, Stewart V (1998). NasR, a novel RNA-binding protein, mediates nitrate-responsive transcription antitermination of the Klebsiella oxytoca M5al nasF operon leader in vitro.. J Mol Biol.

[9] Chai W, Stewart V (1999). RNA sequence requirements for NasR-mediated, nitrate-responsive transcription antitermination of the Klebsiella oxytoca M5al nasF operon leader.. J Mol Biol.

[10] Aravind L, Ponting CP (1997). The GAF domain: an evolutionary link between diverse phototransducing proteins.. Trends Biochem Sci.

[11] Taylor BL, Zhulin IB (1999). PAS domains: internal sensors of oxygen, redox potential, and light.. Microbiol Mol Biol Rev.

[12] Stock AM, Robinson VL, Goudreau PN (2000). Two-component signal transduction.. Annu Rev Biochem.

[13] West AH, Stock AM (2001). Histidine kinases and response regulator proteins in two-component signaling systems.. Trends Biochem Sci.

[14] Galperin MY (2006). Structural classification of bacterial response regulators: diversity of output domains and domain combinations.. J Bacteriol.

[15] Del Papa MF, Perego M (2008). Ethanolamine activates a sensor histidine kinase regulating its utilization in Enterococcus faecalis.. J Bacteriol.

[16] Garsin DA (2010). Ethanolamine utilization in bacterial pathogens: roles and regulation.. Nat Rev Microbiol.

[17] Fox KA, Ramesh A, Stearns JE, Bourgogne A, Reyes-Jara A (2009). Multiple posttranscriptional regulatory mechanisms partner to control ethanolamine utilization in Enterococcus faecalis. PMCID: 2647976.. Proc Natl Acad Sci U S A.

[18] Baker KA, Perego M (2011). Transcription Antitermination by a Phosphorylated Response Regulator and Cobalamin-Dependent Termination at a B12 Riboswitch Contribute to Ethanolamine Utilization in Enterococcus faecalis.. J Bacteriol.

[19] Lupas A, Van Dyke M, Stock J (1991). Predicting coiled coils from protein sequences.. Science.

[20] Morth JP, Feng V, Perry LJ, Svergun DI, Tucker PA (2004). The crystal and solution structure of a putative transcriptional antiterminator from Mycobacterium tuberculosis.. Structure.

[21] Jagadeesan D, Eswaramoorthy M, Rao CN (2009). Investigations of the conversion of inorganic carbonates to methane.. Chem Sus Chem.

[22] Porter SL, Armitage JP (2002). Phosphotransfer in Rhodobacter sphaeroides chemotaxis.. J Mol Biol.

[23] Wemmer DE, Kern D (2005). Beryllofluoride binding mimics phosphorylation of aspartate in response regulators.. J Bacteriol.

[24] Yan D, Cho HS, Hastings CA, Igo MM, Lee SY (1999). Beryllofluoride mimics phosphorylation of NtrC and other bacterial response regulators.. Proc Natl Acad Sci U S A.

[25] Roelofs KG, Wang J, Sintim HO, Lee VT (2011). Differential radial capillary action of ligand assay for high-throughput detection of protein-metabolite interactions.. Proc Natl Acad Sci U S A.

[26] Donaldson GP, Roelofs KG, Luo Y, Sintim HO, Lee VT (2011). A rapid assay for affinity and kinetics of molecular interactions with nucleic acids.. Nucleic Acids Res.

[27] Gao R, Stock AM (2009). Biological insights from structures of two-component proteins.. Annu Rev Microbiol.

[28] Barrick JE (2009). Predicting riboswitch regulation on a genomic scale.. Methods Mol Biol.

[29] Griffiths-Jones S, Bateman A, Marshall M, Khanna A, Eddy SR (2003). Rfam: an RNA family database.. Nucleic Acids Res.

[30] Tsoy O, Ravcheev D, Mushegian A (2009). Comparative genomics of ethanolamine utilization.. J Bacteriol.

[31] Houman F, Diaz-Torres MR, Wright A (1990). Transcriptional antitermination in the bgl operon of E. coli is modulated by a specific RNA binding protein.. Cell.

[32] Aymerich S, Steinmetz M (1992). Specificity determinants and structural features in the RNA target of the bacterial antiterminator proteins of the BglG/SacY family.. Proc Natl Acad Sci U S A.

[33] Dann CE, Wakeman CA, Sieling CL, Baker SC, Irnov I (2007). Structure and mechanism of a metal-sensing regulatory RNA.. Cell.

[34] Yao Z, Weinberg Z, Ruzzo WL (2006). CMfinder–a covariance model based RNA motif finding algorithm.. Bioinformatics.

[35] Smith C, Heyne S, Richter AS, Will S, Backofen R (2010). Freiburg RNA Tools: a web server integrating INTARNA, EXPARNA and LOCARNA.. Nucleic Acids Res.

[36] Bernhart SH, Hofacker IL, Will S, Gruber AR, Stadler PF (2008). RNAalifold: improved consensus structure prediction for RNA alignments.. BMC Bioinformatics.

