# Supplementary material for: The Mechanism for RNA Recognition by ANTAR Regulators of Gene Expression
Source: PLoS Genet. 2012 Jun 7;8(6):e1002666. doi: 10.1371/journal.pgen.1002666 (PMC3369931; doi:10.1371/journal.pgen.1002666)
Supplement: Figure S1 — Seed alignment of ANTAR substrate hits. Previously [17], we had noted the presence of a putative conserved element (denoted by asterisks) upstream of terminator elements in the intergenic regions of eut pathways from Enterococcus, Listeria, and Clostridium species. Our subsequent inspection of these regions (described in this manuscript) revealed the presence of two tandem stem-loops (red = helices; blue = terminal loop residues). Therefore, instead of one putative conserved region (asterisks), it appeared that two conserved hairpins were located upstream of each of the eut terminators from these organisms. We also noted that the first and fourth positions of both terminal loops were an A an G, respectively, for all of these putative regulatory elements. These features are also present in the K. oxytoca nasF and P. aeruginosa amiE leader sequences. Therefore, these general criteria were used to generate a seed alignment for a broader search against bacterial genomes that contained eut pathways (Figure S2; Table S1). (DOCX) [file pgen.1002666.s001.docx]

**Figure S1. Seed alignment of ANTAR substrate hits**. Previously [17], we had noted the presence of a putative conserved element (denoted by asterisks) upstream of terminator elements in the intergenic regions of *eut* pathways from *Enterococcus*, *Listeria*, and *Clostridium* species. Our subsequent inspection of these regions (described in this manuscript) revealed the presence of two tandem stem-loops (red = helices; blue = terminal loop residues). Therefore, instead of one putative conserved region (asterisks), it appeared that two conserved hairpins were located upstream of each of the *eut* terminators from these organisms. We also noted that the first and fourth positions of both terminal loops were an A an G, respectively, for all of these putative regulatory elements. These features are also present in the *K. oxytoca nasF* and *P. aeruginosa* *amiE* leader sequences. Therefore, these general criteria were used to generate a seed alignment for a broader search against bacterial genomes that contained *eut* pathways (Figure S2; Table S1).

************

K oxytoca nasF ..--GGUUUUGGGCAGCGCGCCAAUGGCGGCGCG.........UAUGUCCAG..GGAUAAAGGCGUCC----AGCGGUGC

P aeruginosa ami ..------GUCGAUGUCGCGGGACCGAACCU........AACGCAUACGCAC.AGAGCAAAUGGGCUCU----CCCGGGG

E faecalis eutG ..-------GGUUUC-GUGUACAAUGGCGUAUAC.............AUAAG.GAAGCAAAGACGCUUC---AGACAGAU

E faecalis eutP ..---------TCAG-AAACACAAUGGCGUGUUU.........UAACAAAUC...GGCAAAGGAGCC----CAAGACUAA

E faecalis eutS ..----------------GCACAACGGCGUGC........UUCAAAAUUUAA..GAGCAAAGAAGCUC---CUUAGUAGA

E faecalis eutA ..—-UAUUUCGAACAGAACACCAGUGAUGUUGUUC............AUUGAUUAAGCAAAGGCGCUUAA---AGAAAAG

L monocytogenes eutG ..-UAAAAUAUUUUAACGGUACAAAGGCGUACUGU...........UUACUU...AGCAAAGAAGCU---UUGAGUUGGA

L monocytogenes eutA ..-AAUUCUAUCCAU--GCUACAAAGAAGUAGC............UAUGAAA..AAGCGAUGAAGCUU---AAAGCCAAG

L monocytogenes eutV ..------GAACCGA---GCACAAAGACGUGU..GGAAUUAUAAAUAUAGCA..GAGCAACGGGGCUC---CCUCAAAAA

************
