# Supplementary material for: The Mechanism for RNA Recognition by ANTAR Regulators of Gene Expression
Source: PLoS Genet. 2012 Jun 7;8(6):e1002666. doi: 10.1371/journal.pgen.1002666 (PMC3369931; doi:10.1371/journal.pgen.1002666)
Supplement: Figure S2 — Alignment of ANTAR substrate hits from eut-containing bacteria. The seed alignment of the ANTAR RNA substrates from the eut pathways of E faecalis and Listeria monocytogenes was used to perform an Infernal-based search [28] against 83 eut-containing bacterial genomes. The results of this search are summarized in Table S1. Each of the hits was assigned an arbitrary hit number, which just demarcates their initial ranking in the search results. These hit numbers are included in Table S1 and in Figure S2, so that the respective data can be easily cross-referenced. In general, most hits contained two stem loops (P1 and P2), each of which exhibited a similarly conserved terminal loop sequence. A consensus pattern is shown at the top and bottom of the comparative sequence alignment. The relevant IUPAC codes for this consensus pattern are as follows: R (A or G), Y (C or U), W (A or U), H (A or C or U), S (G or C), D (A or G or U), V (A or C or G), N (G or A or C or U). (DOCX) [file pgen.1002666.s002.docx]

**Figure S2. Alignment of ANTAR substrate hits from *eut-*containing bacteria**. The seed alignment of the ANTAR RNA substrates from the *eut* pathways of *E faecalis* and *Listeria monocytogenes* was used to perform an Infernal-based search [28] against 83 *eut*-containing bacterial genomes. The results of this search are summarized in Table S1. Each of the hits was assigned an arbitrary hit number, which just demarcates their initial ranking in the search results. These hit numbers are included in Table S1 and in Figure S2, so that the respective data can be easily cross-referenced. In general, most hits contained two stem loops (P1 and P2), each of which exhibited a similarly conserved terminal loop sequence. A consensus pattern is shown at the top and bottom of the comparative sequence alignment. The relevant IUPAC codes for this consensus pattern are as follows: R (A or G), Y (C or U), W (A or U), H (A or C or U), S (G or C), D (A or G or U), V (A or C or G), N (G or A or C or U).

:::::::::::::::::::<<<<::------:>>>>::::::::::::::::::::::::::::::::::<<<<.------..>>>>::::::::::::

Consensus aaWWWH.......SYAC..AAHGRH.GUDB................uYYHWW............RRGY.AAHGDV..GCYY.........

Hit# Hit Coordinates A/U cluster **P1 L1 P1** A/U cluster **P2 L2 P2**

001 NC_009089.1/2209707-2209768 ..---UAUAAUGAAA-GUAUCAC..AAAGGC.GUGAUAC............UUUAGUAGA..........AAGC.AAUGGA..GCUU---CUUAGGAAU

002 NC_004668.1/1589608-1589547 ..-UAUAAUGUGGGU---AGCAC..AACGGC.GUGCU............UCaaAAUUUAA..........GAGC.AAAGAA..GCUC---CUUAGUAGA

003 NC_009089.1/2212731-2212788 ..AAUAAUAACUCAU----CUAC..AAAGUA.GUAG..................UUUUAA........UUGAGC.AAAGAG..GCUUAA---GGUUAUU

004 NC_004668.1/1592374-1592320 ..--------AAUCA-GAAACAC..AAUGGC.GUGUUUU...............AACAAA.........UCGGC.AAAGGA..GCCCA---AGACUAAG

005 NC_004557.1/2307829-2307768 ..---AAUAUUAAUA-AUUAUAC..AAAGGC.GUAUAAU............uaUCGGUAA..........GAGC.AAUGGA..GCUC---CUUAAAAUA

006 NC_008555.1/1141572-1141629 ..--AAAAUAUUUUA--CAGUAC..AAAGGC.GUACUG...............UUUACUU...........AGC.AAAGAA..GCU---UUGAGUUAGA

007 NC_003210.1/1202056-1202113 ..--AAUUCUAUCCA--UGCUAC..AAAGAA.GUAGC................UAUGAAA..........AAGC.GAUGAA..GCUU---AAAGCCAAG

008 NC_004668.1/1590877-1590820 ..--GAUAAGGUUUC--GUGUAC..AAUGGC.GUAUAC.................AUAAG.........GAAGC.AAAGAC..GCUUC---AGACAGAU

009 NC_008261.1/1052251-1052307 ..----AAUAAAGAU---AUCAC..AAAGGU.GUGAUUA...............AGUUUU.........CGAGC.AAUGUA..GCUUC---UUAAAGGU

010 NC_003210.1/1198518-1198575 ..--AAAAUAUUUUA--CGGUAC..AAAGGC.GUACUG...............UUUACUU...........AGC.AAAGAA..GCU---UUGAGUUGGA

011 NC_003212.1/1151140-1151197 ..--AAAAUAUUUUA--CGGUAC..AAAGGC.GUACUG...............UUUACUU...........AGC.AAAGAA..GCU---UUGAGUUGGA

012 NC_003212.1/1154678-1154736 ..-AAUUCAAUCCAU---GCUAC..AAAGAA.GUAGC...............UAUGAAAA..........AAGC.GAUGAA..GCUU---AAAGCCAAG

013 NC_008555.1/1145109-1145166 ..--AUUUCAAUCCA--UGCUAC..AAAGAA.GUAGC................UAUGAAA..........AAGC.GAUGAA..GCUU---AAAGCCAAG

014 NC_009633.1/290271-290331 ..--AAUUUCAAACA-AUUGCAC..AAUGGC.GUGCAAU..............GAUUAAA..........AAGC.GAUGAA..GCUU---GCAGGAAGG

015 NC_004668.1/1587130-1587070 ..--UAUUUCGAACA-GAACACC..AGUGAU.GUUGUUC................AUUGA........UUAAGC.AAAGGC..GCUUAA---AGAAAAG

016 NC_009089.1/2208381-2208435 ..AUUUUUAAAUAUA----GCAC..AAUGAA.GUGU...................UAAAA..........GAGC.AAUGGA..GCUC---CAUAGAUAA

017 NC_009009.1/512876-512934 ..----AAUUCAAUA--UAGUAC..AAAGGG.GUACUG............AAGAUAAAUA...........AGC.AAUGAA..GCU---AUGUGAAAAU

018 NC_004668.1/126057-125997 ..-AUUACCUUAGCA---AGUAC..AAUGGU.GUACU.............GUGAACAGUC..........GAGC.AACGGC..GCUC---AAAAAGAUU

019 NC_003210.1/1199836-1199904 .-AUAAUAGAACCGA----GCAC..AAAGAC.GUGU......GGAAUUAUAAAUAUAGCA..........GAGC.AACGGG..GCUC---CCUCAAAAA

020 NC_004557.1/2309190-2309131 ..-UAUAAAAUGAAA--UUGCAC..AAUGAU.GUGCAA...............UAGUAGA..........GAGU.AAUGAA..GCUC---CUAAAGAUU

021 NC_003212.1/1152458-1152526 .uAUAAUAGAACCGA----GCAC..AAAGAC.GUGU......GGAAUAAUAAAUAUAGCA..........GAGC.AACGGG..GCUC---CCUCAAAAA

022 NC_008555.1/1142889-1142957 .uAUAAUAGAACCGA----GCAC..AAAGAC.GUGU......GGAAUAAUAAAUAUAGCA..........GAGC.AACGGG..GCUC---CCUCAAAAA

023 NC_009009.1/466893-466954 ..--------GAAAA-CGAACAC..AACGGA.GUGUUUG......AACUACAUAUAAGUG...........AGC.AACGGU..GCU---ACAAAAAAUG

024 NC_008261.1/1050699-1050753 ..UAUAUUAAAAAUA----ACAC..GAUGUG.GUGU...................UUAGA..........GAGC.AAAGAA..GCUC---CAUAGGAAA

025 NC_009633.1/293287-293341 ..---AAUAAAAUCA---AGCAC..AAUGGU.GUGUU................UGUUCAA...........GGC.AAUGGG..GCC---AAGGAUCUAU

026 NC_003454.1/716227-716290 ..---AAUUAAAUAA-GAUAUAC..AAUGGA.GUAUAUU.........AUAAAAAUAGAA...........AGC.AAUGAA..GCU---GAUGGACAGG

027 NC_009009.1/516636-516693 ..-AAUACUACCAGA---CUAAC..AAUGAU.GUCAG................UCGAAAA..........AAGC.AAUGAC..GCUU---GAAGAAUGU

028 NC_004557.1/2304834-2304775 ..----GAUUAGAGC-CAAAAAC..AAAGAA.GUUUUUG............GCUAGAAAA...........GGC.AACGAA..GCC---CAGAAAAUUU

029 NC_008261.1/1055229-1055285 ..---AAUAACUAUG--AACUGC..GAAGUA.GUGGUU..................UGAA........UUGAGC.AAUGAG..GCUUAA---GGUUAUA

030 NC_007498.2/2447483-2447533 ..--------CACAU---GACAC..AACGGC.GUGUC...............AGGCUUAC...........GGC.AACGAC..GCC---CCAUUCAGGA

031 NC_008261.1/2884578-2884526 ..------AAAAUAU----ACAU..AAAGGA.GUGU...............CGCUUGAGU..........GAGU.GAAGUA..ACUU---UUAGAAAAG

032 NC_009253.1/3046896-3046842 ..AACAACAUACUCU----GAAC..AACGAU.GUUC...................UAGUC..........AGGC.AAUGAU..GCCU---UUUGUAAGA

033 NC_002516.2/1855754-1855810 ..--------GGAAA-AGGCCAC..AGCGAU.GUGGCUU...........UUUUCUUAAA...........AGA.AAAGUC..UCU---CAGUGACAAA

034 NC_007498.2/2456540-2456594 ..---------AACG--GGGCAC..AAUGGC.GUGCCU...........GGCUGAGAAAC...........GGC.AACGAG..GCC---CGGUGGGAUU

036 NC_009654.1/3196287-3196231 ..---AUUAUUGAUU----GUCC..AAUGUA.GGAU.............UUUCUUUGAAU...........GGC.AAAGGC..GCC---UGCGAACACA

037 NC_009253.1/3045296-3045244 ..-----AAUUGAAA---AGCAC..GAUGAU.GUGCU................AACGAAU...........GGC.AAUGAA..GCC---UACAGCGGGU

039 NC_009253.1/3047580-3047527 ..----AACACUAAG---GAUAC..AAUGAU.GUAUC................CGAUUAU...........GGC.AACGAU..GCC---CAGGCAGGAG

040 NC_008726.1/6033961-6033904 ..---AAGAGAGAUC---ACCAC..AAAGGU.GUGGU.............UGACUACACG...........GGC.AAAGUG..GCC---GGUUUUCGGG

044 NC_009654.1/4586404-4586468 ..UUUUUAUCUAAUG----CAAC..AAAGGC.GUUG.......GAAGAGUUUAACCAAGU...........AGU.AAAG--..GCU---UUAUCAAAGU

045 NC_006350.1/1450675-1450740 ..-------ACUAUC---AAUAC..AAAGAA.GUAUU....CGAUUGACAUGAAUCAACGCAA........GGC.AACGGC..GCU---CAUGAGAUUU

049 NC_003212.1/1053865-1053810 ..--AGUACCAAUAU------AC..--AGGA.GU............UUUCCCGUUUUAAA..........AAGU.AAAGCG..ACUU---CUAAUUAAU

056 NC_009633.1/3519585-3519642 ..AUAUUUUUCCAUG-----UAU..AAUGAG.AUA...............UCGUUAGUCC..........UAGG.GAAGGC..CCUA----AAACAAAU

057 NC_007907.1/5011991-5011936 ..-------UGUUAC----UUUU..AAUGCA.GAAA...........UUUUAUAAAUGAA..........GAGC.AAAGGC..GCUC---AUAAGGAAU

059 NC_009085.1/1465272-1465218 ..-------UGCAUC---UGUAC..AAAGGC.GUAUA...............ACUCGGAU........GGAAGA.AAAGGAAAUUAUUU-----UAAAA

062 NC_009009.1/512478-512410 ..--------UGUCU--UUCUGC..AAAGGA.GCAGAG....CUAAUAAUUGGAAAGCUA.....GUGCUAAUC.AACGAA..GCUUAGCAU---AAAG

071 NC_007492.2/3807050-3807001 ..-------AUCGAU----GCGC..AAAGGC.GUGC..................UAUGGA.........AACUC.AAGGGA..GGGUU---AAAGCAUG

072 NC_008228.1/836271-836321 ..--------AAUUU-GGAGCAU..AAGGAU.AUGUUUC.................GUCU...........UGC.AAUGGA..GCA---UUCAGCCAUA

073 NC_009485.1/6902858-6902810 ..-------AUAUCG----UCGC..AAGGAU.GCGG....................AGUG........AAGGGC.AAUGAA..GCUCUU---AGAUAAU

074 NC_008726.1/4556568-4556520 ..--------UAUAG---UGCAC..AUGGAU.GUGCG..................AUAAA.........UGAGC.AAAGU-..GCUUA---CAAAAUAC

079 NC_007086.1/3508976-3509026 ..---------GGUC.----CAC..AAUGGC.GAG.............CCGCCCAUUAUU..........GUGC.AAUGCA..GCAU---CUGGCGAAA

084 NC_008261.1/2492531-2492479 ..--------------AUUUAAU..AAAGGC.CUUAAAU...........UAUUAUUUAA..........GAGC.UGAGGA..GCUU---CUAGAAGGA

086 NC_009972.1/5354031-5353982 ..--UAUAAUGCCCG----CAGC..AACGAC.GCUG.....................AUG...........AGC.AAUGGU..GCU---AAUUCACAUA

089 NC_009654.1/3266578-3266637 ..--AAUAGAACAAA---GCACU..AAUGGC.GUUGU........GGAUAUAUUAGAAGA.............C.AAUGAA..G-----CAAUACCAAU

090 NC_009648.1/4401158-4401213 ..-UAAAAAAUACCG---GCGCC..AACGGC.GUCGU...............ACGCAAUC............GA.AAUGAAGUUC------AGGUUAUU

095 NC_007907.1/4989851-4989798 ..--UAUUAUGUUAA----GUUA..--UGCU.UAAU................CAAAAAUG..........GAGC.AAAGGC..GCUC---AAAGAGACA

108 NC_008228.1/2366834-2366785 ..----UUUUUGGCU-----UAC..AAAGAU.GUA....................UAAAU.......UUGAAGC.AAA--C..GCUCCAA---CUAAAA

109 NC_007498.2/620603-620653 ..------------G---GUCAC..AAAGGU.GUGGC...........AGCCAUAGCAUC...........GGC.AACGAA..GCC---CGCAGGGAUG

110 NC_007907.1/5355923-5355872 ..------UAUAGAA---UCUGC..AGAGAU.GAGA.................UUUCUCU..........GAUC.AAUGAG..GAUU---CAGACAAAA

111 NC_009633.1/2545831-2545893 ..UACAAUAAUCAUU-----UAC..AGGGGG.GUA.........UAAUUUGUCCGAUAAA...........AGC.AACGGC..GCU---AAAAAAAGUA

120 NC_009633.1/914169-914220 ..---AAACCAACUC-------A..AUAGAA.U.................UGGUAAGGAA.........GAGGC.CAUGUA..GCCUU---AUACAAAA

121 NC_007651.1/3312922-3312859 ..---------UAUC---AACAC..AAAGAA.GUAUU....CGAUUGACAUGAAUCAACGCAA........AGC.AACGGU..GCU---CAUGAGAUUU

122 NC_008702.1/828628-828580 ..------UUCCGGA-----UAC..AAAGAC.GUA...................GAGAAG.........GGAGC.AAUGCG..GCAUCU---GAAAGGA

124 NC_009253.1/1438580-1438529 ..--AAUUAUGGAAA---ACCAU..AAAGUU.GUGG................GGCUACUC.............C.AAAGGG..G-----UCAAGCUAUU

125 NC_009253.1/3051528-3051586 ..AUUAUACAAUUAA--------..AAAGUC............AAUGUUUUUGACACAGG...........GGC.AAUGGC..GCC---ACCUGUUAAA

126 NC_009792.1/4243606-4243658 ..--------UAUAC--CUGAAG..AAAGGA.CUUUAG...............AUGCUUU..........UAGC.UACGGC..GCUG---UUAAUUAUU

129 NC_008261.1/782965-782918 ..----------------------------...........AAAUAGAUAUGUAGUUGUUAAA.....AUGAGC.AAUGAA..GCUUAU---ACUGAGU

131 NC_008228.1/2944629-2944570 ..---CAUACUGACA-GAAUUAC..CUCGUU.GUAAUUU.............CUAAGUAA...........GGC.AAUGUA..GCC---CGCAAUGACU

135 NC_007907.1/3304173-3304225 ..------AAUAUCC-----AAU..AAUGUG.GUU..............UUCCCCGAAAC..........CAGC.AAAGAA..GCUG---CCAAUAAAG

137 NC_009972.1/2941344-2941396 ..-----------AA-AUGCAAC..GGAGCC.GUUGUAU.............UUUGGAAU..........GCAC.AAAGGA..GUGC---CAUAAUGCU

141 NC_009972.1/1051162-1051113 ..---------AAUC--AACAAC..AAAGGC.GUUGUU.................................UGC.AAGCGUU.GCA-GCACUUCAUAAC

144 NC_009089.1/2212899-2212857 ..-----------------------------..........AAUUUAAGUAUGUCUUAAAC.........AAGC.AAAGAG..GCUU---AAAGUUAUA

155 NC_008261.1/1476504-1476551 ..---------------GACUAU..AAUGGA.AUAGUU...............UUAGAUA..........GAUC.AACAGA..GUUU---AUAGUUAAU

162 NC_008555.1/363779-363730 ..AAUAAAAAAAGCA----UCAC..AAUGAA.GUGA.....CGCUUUUUUAGAUUAUUUUAAGU...................................

165 NC_003030.1/837937-837877 ..AAAGCAUCAAGCA----GCAC..AAA--U.GUGU............CACUUAAUUGAU..........AAUU.AAAGGA..GGUU---UAUAUUAUG

168 NC_008555.1/1037360-1037305 ..----AGUACCAAU------AU..ACAGGA.GU...........UUUCUUAUUUUAAAA...........AGU.AAAGCG..ACU---CCUUACUACU

169 NC_006177.1/1341787-1341840 ..------UGAAAUU---ACCAG..AAAGAA.AUGGU..............CGCUGAUUU...........CGC.AAGAAA..GCG---CCGAAUUAAG

170 NC_009485.1/4915593-4915541 ..--GAUAUGGGCAA-------C..AAAGGC.G.................CACUAAUGAC.........GACGC.AAUGAA..GCGUU---UUCUUGUC

171 NC_007907.1/5231930-5231986 ..-----AAUCCGGA----AUAC..AAUGAA.GUAU.............GUCUCUUACAA.........AGAGC.AAA-AA..GCAUU--AAUGGCAGA

172 NC_009085.1/2195062-2195009 ..----UCAAUGAGC------AC..AAUGGA.GU..............UGUCUAAUGAUU..........GACA.AAAGUA..AGUC---CUCACUAAG

178 NC_009654.1/397442-397492 ..-------GAAUGG--AACCCC..AAUGAA.GUGGUU...............GUAGACG...........AAG.-AUGCG..CUU----AUUAUCAAA

181 NC_009089.1/3347122-3347062 ..UAUCUGGAGUAAA---AAAAC..AAGGUA.GUUUU.............AUAACUGAUG..........AAGG.AAAGAA..CUUU----AUAUCAUA

182 NC_008261.1/615455-615510 ..AAUGGGAUAUGUC----UUAC..AAUGAU.GUAA.............AAUUAAUAAUG............GU.AAUGGC..UC------AUGAAGAU

183 NC_009972.1/6013611-6013559 ..------AAUAAAG---GCAAU..AAAGCC.AUUGU............UAUACUCGCGC...........---.AACGAA..---ACAUCCUGCAAAU

186 NC_008555.1/1524944-1525001 ..----AGUAAUGGU---AGCAA..AAAAUC.UUGCU............UUGCUUUAUCU...........AAGCGAUGUA..CUU----CAUCCAAAA

188 NC_009972.1/6041559-6041616 ..UUUCGUAUAGAAU-----AUC.CACAAUCUGAU.....................CCAA........AUAAGC.AAUGGC..GCUUAU---CGCUAGG

190 NC_008555.1/2274563-2274505 ..-AAUAUUUUUCCA-----GUG..AAGACA.UAC.............GCAUGCUAUAAU..........GAGU.GAAGAA..ACUU---CUUACUACA

191 NC_009439.1/2217515-2217576 ..GAUAAAGUCACGA----AAUC..AGGAAU.GAUU...............GCUCCAUAA.......AUGGAGC.AAUGGA..GUUCCGU---AUGAAG

197 NC_010001.1/4467323-4467260 ..AUAUUGUAUAAGG------AU..AAUGGA.AU........GCCCUCAUUUUUAUUCAA...........UGC.AAGGAA..GCA---CUUUGCGUAA

202 NC_009633.1/747949-747895 ..--AAAAAGUACAA---UUCAC..AAAGGU.GUGGA.............AUUAUUAAAC...........---.AAAGGA..----AACCUUCCCUUU

205 NC_008555.1/2367111-2367166 ..------UGUAGCA-----GAU..AACGGG.GUU..........CCAUAUUAUGUCCUC...........AGC.AAAGAU..GCU---CAAGACAAAU

206 NC_003212.1/797624-797571 ..------AAUUAGA----CGAA..AAAGGA.AUCG............AACUAGAAAUCC............GU.AAAGUC..GC---UUCUGAUAAUA

212 NC_008752.1/2344874-2344933 ..--AAUAAAGACCA-CGGAUGC..AAAGAG.GCAUACG..............AUAAGGC...........GGC.AGAAGA..GCU---UAGAAAUAUG

213 NC_008261.1/1911525-1911475 ..----AGUAAGUUA----GCAC..AA----.GUGC.................AAACUGA........AAAAGU.UAUGGA..ACUUUU---AAAGGAA

215 NC_009654.1/4907313-4907378 ..--------UAUAU-GGAACAA..AAAGAC.CUGUUUC..UAGACAAUGGCAAAGCCUA...........CGC.AAUGAC..GCG---CCCAUUAAAC

226 NC_009654.1/3246922-3246868 ..-CAAAAUGUAAGA--GAGCAA..A-----.UUGCUU..............UUACUAAA...........AGC.AAAGGG..GCU---AUCGAAAAUG

227 NC_008555.1/1889095-1889040 ..------GAACUCG---AGAAC..AAAGAU.GUUCU.............UAUUCAAAAU...........GGU.AAAGUCA.ACU---UGAUUGCAGA

232 NC_004668.1/3055751-3055698 ..--UAUCUAUUGAC------AA..AAAGGG.UU.........................U.UCCUUGUAAAAGU.AAAGGU..ACUUUUACAAGGA---

235 NC_007907.1/4926294-4926262 ..-----------AU---AGAAC..AAAGGC.GUUCU........AAGGACCCUAAUAGG.......................................

245 NC_009253.1/1330424-1330483 ..AAUAAUAACUGGU----GUAC..CCAGGG.GUAC....................CUGC..........UCAU.AAAGCCA.AAGG-AUAUCAUUAUU

248 NC_003030.1/2844755-2844710 ..-----------------------------.......AAUUUUUUAAUAUUACAUUUAG.........UGAGC.AAAGGA..GCUCA---AACGAUUA

250 NC_008313.1/85753-85809 ..----AAUCUGUAA----UUAC..AACGGU.GUAA.............GUCAUUGAUUU...........UGC.GAUGUU..GCG---CAUGCACAAU

251 NC_003212.1/435555-435608 ..-----UUUUCCAA-----GGC..AAAGAAcGCC..............ACUUGGAUGAA...........AGC.AAUGAA..GCU---GAAACAAAAA

253 NC_008555.1/2791354-2791410 ..AAAAUAGACCACU----GCUC..AG----.GAGU............UACAUCAUAACU...........UGC.AAUGGA..GCG---UAGCAAAAAU

257 NC_009445.1/6579310-6579358 ..--------UAUCC----GCAC..UACGAC.GUGC.................UCGCCAA..........GCGC.AACGGA..GCGU---CGUGGAACG

260 NC_008261.1/2284873-2284817 ..--UGUUUAACCAC-------U..AAUGGG.A............UUUUACUAGGAGAUA..........AAGU.AAAGGA..ACUU---AGUGAAAAU

263 NC_009256.1/303565-303512 ..--------CAGAC----GCAC..AAUGGU.GUGC..........AGGUUGAAGUCUUU..........GAGC.--AGUU..GCUC---CUUGAUGAC

271 NC_009654.1/3519147-3519088 ..ACAUAGCCACAUC-------A..UGUGGC.U.............UAUUUUAUUUAAUA.........UGUGC.AAGGAA..GCAUA---CUAUGAAU

273 NC_009089.1/3612674-3612623 ..-----UUACUGGG----AUAU..----GA.GUAU.............AAGAAGGUUUU..........GAGC.AAAGAA..GCUC---UAAAGAAAA

274 NC_007907.1/1577383-1577430 ..-----------------------------.....AAAUUAUAUAGAAGCUCCAUGAGA..........AAGC.AAAGAA..GUUU---CAGUCAGCA

277 NC_009009.1/1229549-1229604 ..--AAUCAAGUUCU------AC..AUUGCC.GU.................UGUCGGUGA..........CAGC.AAUGAA..GAUG---CUCAAGCAA

284 NC_010001.1/3828409-3828358 ..---------UAUA------CU..AAAGUG.GG..........AGGCCAUUAUAUAUUC...........AGC.AAGA--..GCU--UUAGAGGAGUA

293 NC_010001.1/4756130-4756080 ..---AAUACAAACA-----UAG..AGUGUA.UUA..................UAUUUAU...........AGC.AAGGAA..GCU---CGAUAGAAUC

295 NC_009633.1/3445688-3445635 ..-----------------------------.AUUAUUGUAUACAAUAAUAUACUAAAUCAA.......UGGGC.AAUGGC..GUCCA---CGAUUAAA

297 NC_009654.1/4205235-4205175 ..---AAUUUCAUAA-GUUGGCC..AAUGUC.GGUUAAC............UUAGUCAAU...........GGC.ACCGUC..GCC---CGCUACUAAA

300 NC_009253.1/512043-511971 ..AUAUAGUGAAGAU----AAAC..AAUGGU.GCUUU..UCUCCCAUUGUUUUAAUUGCUCCA.........GC.AAAGAA..GC----CAAGGAUUUU

301 NC_003212.1/1436682-1436739 ..---AAUUGUGACA---GUUUCa.AAAGAA.GAAAU...............CAUGAAAA..........AAGC.AACGGA..ACUU---CGGGAUGCU

306 NC_007907.1/3208212-3208158 ..-----AAUUCAAA---GCGCC..AAAA--.GGCGU...........GGUUACGGAAGA..........GAUA.AAAGCU..UUUU---AAAAGAG

308 NC_007907.1/4506948-4506896 ..----AAUCAAAUG------AG..AAAGGU.UU..............AAUUUGUGUCAG.........CAGAA.AGAGGC..UUCUG-----AGAAUU

311 NC_009089.1/3523958-3524018 ..--AAUCAAAUUAA-UAACUAC..AUAG--.GUAGUUA...........CUAAUCUUUU...........AUC.AAAGGA..GUU---UCUAUAUGGA

314 NC_010001.1/9120-9167 ..-----AAUUUACU----CCAC..AAAGAC.GUGG...............UGGUAAAGG..........UGUG.AAAUGC..UACA---------AGA

318 NC_009085.1/2559198-2559143 ..-----AUACCUAU-------C..AUUGGU.G..............CAAUACAUUAAUC.......AGUGAGC.CAUGAA..GCUUAC---UAAAAUC

320 NC_007907.1/1638233-1638292 ..-------GAACUA-----UUAGgAAUGGC.UAA........GUCAACACUGUAUCGCA..........AAGC.AAAAGA..GCUU---AAUAUAGAU

321 NC_008261.1/298698-298750 ..------UGGCAAU----CUAC..ACA---.GUA.............GUUUCUAAGAAC.........UGAAC.CAUGAA..GUUCA---AAAACAUU

329 NC_007907.1/3759115-3759065 ..----UAACCCGUU----UGAC..------.GUCA............UUAAAUUUAGAA...........AGC.AAUGAC..ACU---CAGGAUUAUA

331 NC_009089.1/624254-624307 ..------UAUCCCA----UUUAaaAUGUUA.UAAA................GCAUUACA..........GGGG.AAAGAU..UCUC---CCUGCGAAU

334 NC_008555.1/808121-808180 ..---CAUAUUUUAC--GUGUCC..AAGCGA.GUAUGU.............CAUUGGUCA..........GAAC.AAAGAA..GUAC---AAGAAAAAC

339 NC_007907.1/4096117-4096177 ..AACAUUGGGAGGA---ACGCC..UAAGUU.GGUGU..............AAUAGGUUU..........UAGC.AAUGUU..GCUG---CCGAUAAUG

341 NC_009633.1/3606681-3606626 ..-AAUAGAUUGCAA---UAGAC..AAUGAC.GUCUA......................C.........AAUUA.AAAGGAG.UAAUU-UCCUGAUAUU

343 NC_008555.1/973654-973700 ..----GAAUUCUUG-------C..AAUGCU.-------.................UUUU......GAAACAGC.AAAGUG..GCUUUUUC---UGGUG

344 NC_003030.1/963306-963364 ..CAUAUGGUUACGA---UAUAC..AAAGGU.UUAUA...............GAAAUAGA...........UGG.AAUGGA..UCA---GUGGGCUAAG

350 NC_009972.1/5840194-5840237 ..-------AAUAUG----GUAC..AAAGGA.GUGU.....................ACA............GC.AAUGAA..GC---GUAUUUUGAGC

354 NC_008228.1/3623063-3623006 ..-------AAUAC-AGAUACAC.CCAGGUU.GAGUUUUU.............UGCUGUU..........GAGU.AAAGUC..ACUU---UUUGCAAAA

361 NC_004668.1/293606-293663 ..AAAUGAUUAUUUG----CUAC..AAAGGC.GAAG..................AUUUAA.........AAGUG.CAAGA-..CACUU--GUAGCACAU

370 NC_008261.1/1537572-1537621 ..------------U----AGGGAAAAGUGA.CCUU..............UCAGUAGAUC..........UAGC.AAAAA-..GCUA--AAUGGUGAAG

374 NC_009253.1/3494870-3494808 ..-AGUAUAAUCAAG-----AUA..AAUGAA.UAU.........GCACAUUAUGCAACUA..........AGGC.AAAGAA..GCCU---ACAACGGGU

376 NC_003212.1/1817567-1817502 ..AUAAUGUAUCUAU-----UAC..AGGGAG.GUA.............UUUAUAAGUGAA.........UAACA.CGCCUA..UGUUG-CCUCCAGAAA

378 NC_009654.1/5040125-5040074 ..----UAUUUUGCU----GUAC..AAAGUU.GCA..................CAGGAGU..........GAGC.AAAGUU..ACUC---CUUUAAACU

381 NC_008555.1/2054605-2054550 ..-AAAUCUGUUUAG----GAAC..AAUGGG.GUUU...............GGUGAUAAA...........GAC.AAAUGG..GUU----CAUAAAUGG

383 NC_007498.2/2520336-2520403 ..---GAUAAGUUCC-UUUUCAC..CGAGAC.GUAGAAGA.....AGGAACCCUCCCAUU...........AGC.AAAGCA..GCU---CCGACCAAAA

386 NC_009089.1/3448756-3448700 ..---GAUAAAAUAC----UAGC..AAAGAG.GCUG..................................UCUC.AAAAUA..GAGA-GAACCUCUUUUUCU

389 NC_008228.1/2592969-2592926 ..-----------------------------..........AUCCUGAUGGUAUUUCUUAG........UGCGC.AAAGAA..GCGUA---AGCAGAUU

390 NC_007953.1/677351-677304 ..-----------------------------........GAACGCGAGAGACUUAUUCUGG......AAGAAGC.AACGAA..GCUUUUU---GCCGAA

399 NC_009633.1/3210506-3210452 ..-----AAGAAUAA----UUCG..AUAGA-.CGAA..............GCCCAUUGAAC.........GAAC.AAUGAA..GUUU---ACGAUGGUU

:::::::::::::::::::<<<<::------:>>>>::::::::::::::::::::::::::::::::::<<<<.------..>>>>::::::::::::

aaWWWH.......SYAC..AAHGRH.GUDB................uYYHWW............RRGY.AAHGDV..GCYY.........

Relevant IUPAC Codes

R A or G

Y C or U

W A or U

H A or C or U

S G or C

D A or G or U

V A or C or G

N G or A or C or U
