# Supplementary material for: The Mechanism for RNA Recognition by ANTAR Regulators of Gene Expression
Source: PLoS Genet. 2012 Jun 7;8(6):e1002666. doi: 10.1371/journal.pgen.1002666 (PMC3369931; doi:10.1371/journal.pgen.1002666)
Supplement: Figure S3 — Example of an ANTAR target RNA predicted to influence translation rather than transcription attenuation. A subset of the putative ANTAR target RNAs identified in eut-containing organisms overlapped with an intrinsic transcription terminator, despite the fact that inclusion of an intrinsic terminator was not part of the original search criteria (highlighted in yellow in Table S1). These ANTAR target RNAs are therefore most likely to be responsible for controlling transcription attenuation. However, it is likely that many of the remaining putative ANTAR hits are still functional genetic elements, but that they employ a regulatory mechanism other than transcription attenuation, such as controlling the efficiency of translation initiation. To highlight this possibility, this supplementary figure includes an example of a putative ANTAR target RNA that overlaps a ribosome-binding site and the translation start of the downstream gene, which in this case is a methyl-accepting chemotaxis protein. Follow-up experimentation would be required to verify translational control by this particular putative ANTAR-responsive element. (DOCX) [file pgen.1002666.s003.docx]

**
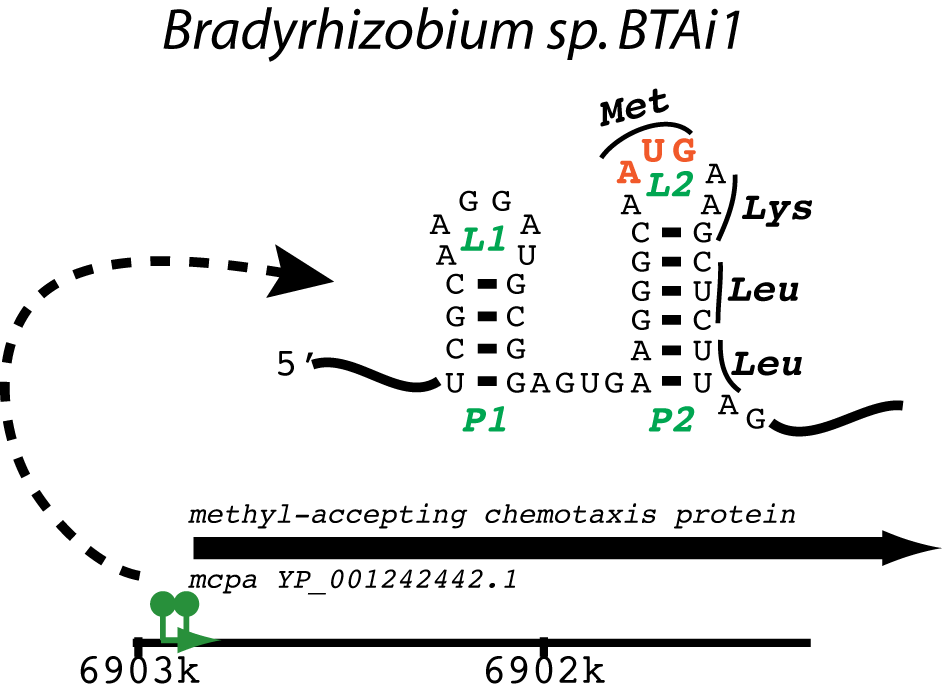
**

**Figure S3. Example of an ANTAR target RNA predicted to influence translation rather than transcription attenuation**. A subset of the putative ANTAR target RNAs identified in *eut*-containing organisms overlapped with an intrinsic transcription terminator, despite the fact that inclusion of an intrinsic terminator was not part of the original search criteria (highlighted in yellow in Table S1). These ANTAR target RNAs are therefore most likely to be responsible for controlling transcription attenuation. However, it is likely that many of the remaining putative ANTAR hits are still functional genetic elements, but that they employ a regulatory mechanism other than transcription attenuation, such as controlling the efficiency of translation initiation. To highlight this possibility, this supplementary figure includes an example of a putative ANTAR target RNA that overlaps a ribosome-binding site and the translation start of the downstream gene, which in this case is a methyl-accepting chemotaxis protein. Follow-up experimentation would be required to verify translational control by this particular putative ANTAR-responsive element.
