# Supplementary material for: The Mechanism for RNA Recognition by ANTAR Regulators of Gene Expression
Source: PLoS Genet. 2012 Jun 7;8(6):e1002666. doi: 10.1371/journal.pgen.1002666 (PMC3369931; doi:10.1371/journal.pgen.1002666)
Supplement: Figure S4 — Purification of EutV. Purification of EutV on a DEAE column following purification on TALON affinity resin. EutV elutes as two peaks (black), which differ in the ratio of absorbance 260 nm/280 nm (blue). The first peak is enriched in free EutV whereas the second peak contains EutV contaminated with nucleic acids. (DOCX) [file pgen.1002666.s004.docx]

**
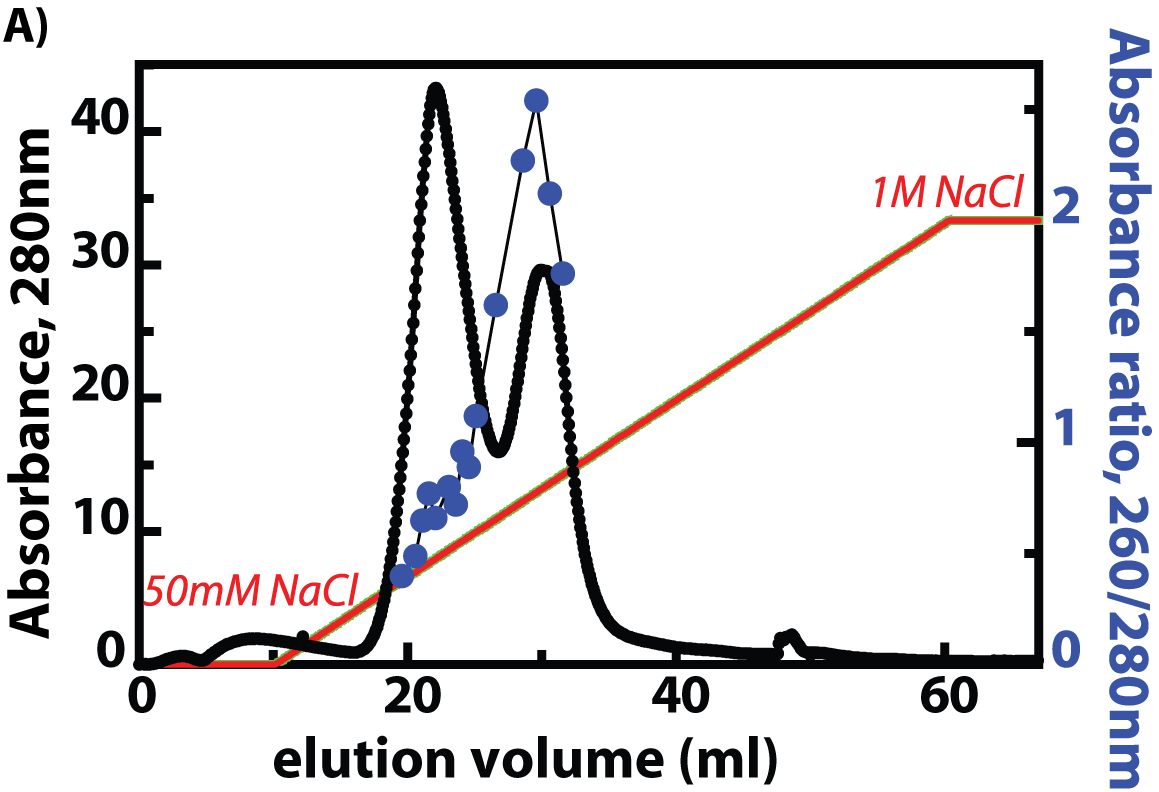
**

**Figure S4. Purification of EutV.** A) Purification of EutV on a DEAE column following purification on TALON affinity resin is shown. EutV elutes as two peaks (black), which differ in the ratio of absorbance 260nm/280nm (blue). The first peak is enriched in free EutV whereas the second peak contains EutV contaminated with nucleic acids.
