# Supplementary material for: The Mechanism for RNA Recognition by ANTAR Regulators of Gene Expression
Source: PLoS Genet. 2012 Jun 7;8(6):e1002666. doi: 10.1371/journal.pgen.1002666 (PMC3369931; doi:10.1371/journal.pgen.1002666)
Supplement: Table S1 — Covariance Search Results for Discovery of ANTAR Substrates in eut-Containing Bacteria. (DOCX) [file pgen.1002666.s005.docx]

**Table S1. Covariance Search Results for Discovery of ANTAR Substrates in *eut*-Containing Bacteria**

^a^As reported for Pfam family PF03861

^b^ANTAR architectures range from 1 to 17, as depicted in Figure 5A

^c^RNA Hit # is a reflection of the initial ranking of the hits that resulted from the Infernal covariance search. They are included herein simply as a means for cross-referencing data in Table S1 with the comparative sequence alignment shown in Figure S1.

^d^RNA Hit Score refers to the numerical score assigned to each hit by the Infernal search. A cut-off of 10 was employed in this study to reduce occurrence of false positives.

^e^RNA Hit Coordinates in this Table specifically refer to the coordinates for the putative ANTAR target RNA hit as recovered by the Infernal. Rows that are marked by yellow highlighting specifically denote those RNA hits that overlapped with a putative intrinsic transcription terminator, as predicted by TransTerm software. Since inclusion of an intrinsic transcription terminator was not part of the original search criteria, the observation that many of the RNA hits overlap with terminator hairpins provides a high degree of confidence in these particular hits, and suggests that they regulate downstream gene expression via transcription attenuation.

| **Genome accession #** | **ORGANISM NAME** | **# of ANTARS^a^** | **ANTAR protein accession #** | **ANTAR archi-tecture^b^** | **ANTAR protein Positions** | **RNA hit #^c^** | **RNA hit Score^d^** | **RNA hit Positions^e^** |
| --- | --- | --- | --- | --- | --- | --- | --- | --- |
|  |  |  |  |  |  |  |  |  |
| ***eut*-Containing Organisms with Putative ANTAR Domain Proteins** | | | | | | | | |
| NC_002516.2 | Pseudomonas aeruginosa PAO1 | 2 | NP_250476.1 | 1 | 1933054-1933632 | 33 | 20.98 | 1855754-1855810 |
|  |  |  | NP_252053.1 | 2 | 3773991-3774581 |  |  |  |
| NC_002947.3 | Pseudomonas putida KT2440 | 1 | NP_744243.1 | 1 | 2385299-2385874 |  |  |  |
| NC_003030.1 | Clostridium acetobutylicum ATCC 824 | 1 | NP_349327.1 | 1 | 2846301-2846855 | 165 | 12.34 | 837937-837877 |
|  |  |  |  |  |  | 248 | 11.33 | 2844755-2844710 |
|  |  |  |  |  |  | 344 | 10.45 | 963306-963364 |
| NC_003210.1 | Listeria monocytogenes EGD-e | 1 | NP_464697.1 | 1 | 1200051-1200632 | 7 | 44.42 | 1202056-1202113 |
|  |  |  |  |  |  | 10 | 41.67 | 1198518-1198575 |
|  |  |  |  |  |  | 19 | 31.52 | 1199836-1199904 |
| NC_003212.1 | Listeria innocua Clip11262 | 1 | NP_470473.1 | 1 | 1152673-1153254 | 11 | 41.67 | 1151140-1151197 |
|  |  |  |  |  |  | 12 | 40.52 | 1154678-1154736 |
|  |  |  |  |  |  | 21 | 30.48 | 1152458-1152526 |
|  |  |  |  |  |  | 49 | 16.58 | 1053865-1053810 |
|  |  |  |  |  |  | 206 | 11.86 | 797624-797571 |
|  |  |  |  |  |  | 251 | 11.3 | 435555-435608 |
|  |  |  |  |  |  | 301 | 10.84 | 1436682-1436739 |
|  |  |  |  |  |  | 376 | 10.19 | 1817567-1817502 |
| NC_003454.1 | Fusobacterium nucleatum ATCC 25586 | 1 | NP_602990.1 | 1 | 714266-714844 | 26 | 27.73 | 716227-716290 |
| NC_004129.6 | Pseudomonas fluorescens Pf-5 | 0 |  |  |  |  |  |  |
| NC_004463.1 | Bradyrhizobium japonicum USDA 110 | 1 | NP_771213.1 | 1 | 5065518-5066108 | 43 | 17.18 | 32196-32256 |
| NC_004557.1 | Clostridium tetani E88 | 1 | NP_782732.1 | 1 | 2306270-2306848 | 5 | 45.12 | 2307829-2307768 |
|  |  |  |  |  |  | 20 | 31.38 | 2309190-2309131 |
|  |  |  |  |  |  | 28 | 23.12 | 2304834-2304775 |
| NC_004578.1 | Pseudomonas syringae pv. tomato str. DC3000 | 2 | NP_792125.1 | 1 | 2554122-2554697 |  |  |  |
|  |  |  | NP_792234.1 | 2 | 2669003-2669635 |  |  |  |
| NC_004668.1 | Enterococcus faecalis V583 | 1 | NP_815343.1 | 1 | 1588529-1589101 | 2 | 46.98 | 1589608-1589547 |
|  |  |  |  |  |  | 4 | 45.69 | 1592374-1592320 |
|  |  |  |  |  |  | 8 | 43.84 | 1590877-1590820 |
|  |  |  |  |  |  | 15 | 39.23 | 1587130-1587070 |
|  |  |  |  |  |  | 18 | 31.97 | 126057-125997 |
|  |  |  |  |  |  | 232 | 11.55 | 3055751-3055698 |
|  |  |  |  |  |  | 361 | 10.3 | 293606-293663 |
| NC_005085.1 | Chromobacterium violaceum ATCC 12472 | 1 | NP_901896.1 | 1 | 2401488-2402078 |  |  |  |
| NC_005296.1 | Rhodopseudomonas palustris CGA009 | 2 | NP_949044.1 | 1 | 4185092-4185676 |  |  |  |
|  |  |  | NP_947752.1 | 2 | 2734103..2734768 |  |  |  |
| NC_006177.1 | Symbiobacterium thermophilum IAM 14863 | 1 | YP_075875.1 | 1 | 2184245-2184892 | 169 | 12.28 | 1341787-1341840 |
| NC_006351.1 | Burkholderia pseudomallei K96243 1 | 1 | YP_111254.1 | 1 | 1687254..1687850 | 45 | 17.06 | 1450675-1450740 |
| NC_007086.1 | Xanthomonas campestris pv. campestris str. 8004 | 1 | YP_243256.1 | 1 | 2628454-2629026 | 79 | 14.45 | 3508976-3509026 |
| NC_007492.2 | Pseudomonas fluorescens Pf0-1 | 1 | YP_347516.1 | 1 | 1985593-1986168 | 71 | 14.79 | 3807050-3807001 |
| NC_007498.2 | Pelobacter carbinolicus DSM 2380 | 1 | YP_357501.1 | 1 | 2439119-2439682 | 30 | 21.73 | 2447483-2447533 |
|  |  |  |  |  |  | 34 | 20.74 | 2456540-2456594 |
|  |  |  |  |  |  | 109 | 13.65 | 620603-620653 |
|  |  |  |  |  |  | 383 | 10.13 | 2520336-2520403 |
| NC_007651.1 | Burkholderia thailandensis E264 I | 1 | YP_439364.1 | 1 | 1359484-1360080 | 121 | 13.33 | 3312922-3312859 |
| NC_007907.1 | Desulfitobacterium hafniense Y51 | 1 | YP_520560.1 | 1 | 4924247-4924819 | 57 | 16 | 5011991-5011936 |
|  |  |  |  |  |  | 95 | 14.03 | 4989851-4989798 |
|  |  |  |  |  |  | 110 | 13.63 | 5355923-5355872 |
|  |  |  |  |  |  | 135 | 13 | 3304173-3304225 |
|  |  |  |  |  |  | 171 | 12.27 | 5231930-5231986 |
|  |  |  |  |  |  | 235 | 11.48 | 4926294-4926262 |
|  |  |  |  |  |  | 274 | 11.03 | 1577383-1577430 |
|  |  |  |  |  |  | 306 | 11.78 | 3208212-3208158 |
|  |  |  |  |  |  | 308 | 11.76 | 4506948-4506896 |
|  |  |  |  |  |  | 320 | 10.66 | 1638233-1638292 |
|  |  |  |  |  |  | 329 | 10.57 | 3759115-3759065 |
|  |  |  |  |  |  | 339 | 10.47 | 4096117-4096177 |
| NC_007908.1 | Rhodoferax ferrireducens T118 | 1 | YP_523801.1 | 1 | 2811256-2811903 |  |  |  |
| NC_007953.1 | Burkholderia xenovorans LB400 3 | 2 | YP_554256.1 | 1 | 2207166-2207741 | 390 | 10.09 | 677351-677304 |
|  |  |  | YP_556511.1 | 2 | 1373702-1374322 |  |  |  |
| NC_008228.1 | Pseudoalteromonas atlantica T6c | 1 | YP_662099.1 | 1 | 3064870-3065508 | 72 | 14.78 | 836271-836321 |
|  |  |  |  |  |  | 108 | 13.66 | 2366834-2366785 |
|  |  |  |  |  |  | 131 | 13.09 | 2944629-2944570 |
|  |  |  |  |  |  | 354 | 10.38 | 3623063-3623006 |
|  |  |  |  |  |  | 389 | 10.1 | 2592969-2592926 |
| NC_008261.1 | Clostridium perfringens ATCC 13124 | 1 | YP_695337.1 | 1 | 1053227-1053802 | 9 | 42.19 | 1052251-1052307 |
|  |  |  |  |  |  | 24 | 29.37 | 1050699-1050753 |
|  |  |  |  |  |  | 29 | 22.45 | 1055229-1055285 |
|  |  |  |  |  |  | 31 | 21.68 | 2884578-2884526 |
|  |  |  |  |  |  | 84 | 14.3 | 2492531-2492479 |
|  |  |  |  |  |  | 129 | 13.15 | 782965-782918 |
|  |  |  |  |  |  | 155 | 12.59 | 1476504-1476551 |
|  |  |  |  |  |  | 182 | 12.13 | 615455-615510 |
|  |  |  |  |  |  | 213 | 11.75 | 1911525-1911475 |
|  |  |  |  |  |  | 260 | 11.23 | 2284873-2284817 |
|  |  |  |  |  |  | 321 | 10.66 | 298698-298750 |
|  |  |  |  |  |  | 370 | 10.33 | 1537572-1537621 |
| NC_008313.1 | Ralstonia eutropha H16 1 | 1 | H16_A0360 | 2 | 378277-378855 | 250 | 11.32 | 85753-85809 |
| NC_008435.1 | Rhodopseudomonas palustris BisA53 | 1 | YP_780199.1 | 1 | 1432548-1433132 |  |  |  |
| NC_008555.1 | Listeria welshimeri serovar 6b str. SLCC5334 | 1 | YP_849327.1 | 1 | 1143113-1143685 | 6 | 44.46 | 1141572-1141629 |
|  |  |  |  |  |  | 13 | 40.11 | 1145109-1145166 |
|  |  |  |  |  |  | 22 | 30.48 | 1142889-1142957 |
|  |  |  |  |  |  | 162 | 12.38 | 363779-363730 |
|  |  |  |  |  |  | 168 | 12.3 | 1037360-1037305 |
|  |  |  |  |  |  | 186 | 12.09 | 1524944-1525001 |
|  |  |  |  |  |  | 190 | 12.06 | 2274563-2274505 |
|  |  |  |  |  |  | 205 | 11.87 | 2367111-2367166 |
|  |  |  |  |  |  | 227 | 11.65 | 1889095-1889040 |
|  |  |  |  |  |  | 253 | 11.29 | 2791354-2791410 |
|  |  |  |  |  |  | 334 | 10.49 | 808121-808180 |
|  |  |  |  |  |  | 343 | 10.46 | 973654-973700 |
|  |  |  |  |  |  | 381 | 10.17 | 2054605-2054550 |
| NC_008609.1 | Pelobacter propionicus DSM 2379 | 1 | YP_903109.1 | 1 | 3818888-3819505 | 0 |  |  |
| NC_008702.1 | Azoarcus sp. BH72 | 1 | YP_932445.1 | 4 | 1009579-1010880 | 122 | 13.33 | 828628-828580 |
| NC_008726.1 | Mycobacterium vanbaalenii PYR-1 | 8 | YP_953653.1 | 1 | 3013229-3013870 | 40 | 18.75 | 6033961-6033904 |
|  |  |  | YP_951892.1 | 2 | 1100257-1100880 | 74 | 14.76 | 4556568-4556520 |
|  |  |  | YP_953369.1 | 2 | 2695467-2696174 |  |  |  |
|  |  |  | YP_955716.1 | 2 | 5282892-5283578 |  |  |  |
|  |  |  | YP_956111.1 | 2 | 5711761-5712021 |  |  |  |
|  |  |  | YP_951893.1 | 3 | 1100918-1101676 |  |  |  |
|  |  |  | YP_951894.1 | 3 | 1101673-1102473 |  |  |  |
|  |  |  | YP_956498.1 | 5 | 6116777-6117454 |  |  |  |
| NC_008752.1 | Acidovorax citrulli AAC00-1 | 1 | YP_970502.1 | 1 | 2360151-2360777 | 212 | 11.76 | 2344874-2344933 |
| NC_009009.1 | Streptococcus sanguinis SK36 | 1 | YP_001034513.1 | 1 | 514625-515200 | 17 | 32.53 | 512876-512934 |
|  |  |  |  |  |  | 23 | 29.83 | 466893-466954 |
|  |  |  |  |  |  | 27 | 24.35 | 516636-516693 |
|  |  |  |  |  |  | 62 | 15.56 | 512478-512410 |
|  |  |  |  |  |  | 277 | 11.03 | 1229549-1229604 |
| NC_009085.1 | Acinetobacter baumannii ATCC 17978 | 1 | YP_001085035.1 | 1 | 2322099-2322524 | 59 | 15.69 | 1465272-1465218 |
|  |  |  |  |  |  | 172 | 12.23 | 2195062-2195009 |
|  |  |  |  |  |  | 318 | 10.68 | 2559198-2559143 |
| NC_009089.1 | Clostridium difficile 630 | 1 | YP_001088417.1 | 1 | 2210754-2211329 | 1 | 48.84 | 2209707-2209768 |
|  |  |  |  |  |  | 3 | 46.94 | 2212731-2212788 |
|  |  |  |  |  |  | 16 | 34.25 | 2208381-2208435 |
|  |  |  |  |  |  | 144 | 12.76 | 2212899-2212857 |
|  |  |  |  |  |  | 181 | 12.15 | 3347122-3347062 |
|  |  |  |  |  |  | 273 | 11.09 | 3612674-3612623 |
|  |  |  |  |  |  | 311 | 10.73 | 3523958-3524018 |
|  |  |  |  |  |  | 331 | 10.55 | 624254-624307 |
|  |  |  |  |  |  | 386 | 10.13 | 3448756-3448700 |
| NC_009253.1 | Desulfotomaculum reducens MI-1 | 4 | YP_001112648 | 1 | 1381401-1382066 | 32 | 21.25 | 3046896-3046842 |
|  |  |  | YP_001114137.1 | 1 | 3046903-3047565 | 37 | 19.21 | 3045296-3045244 |
|  |  |  | YP_001114140.1 | 1 | 3050797-3051372 | 39 | 18.76 | 3047580-3047527 |
|  |  |  | YP_001114193 | 1 | 3109367-3109942 | 82 | 14.39 | 3108831-3108783 |
|  |  |  |  |  |  | 124 | 13.28 | 1438580-1438529 |
|  |  |  |  |  |  | 125 | 13.2 | 3051528-3051586 |
|  |  |  |  |  |  | 245 | 11.36 | 1330424-1330483 |
|  |  |  |  |  |  | 300 | 10.85 | 512043-511971 |
|  |  |  |  |  |  | 374 | 10.2 | 3494870-3494808 |
| NC_009256.1 | Burkholderia vietnamiensis G4 1 | 1 | YP_001117465.1 | 1 | 1945252-1945845 | 263 | 11.22 | 303565-303512 |
| NC_009439.1 | Pseudomonas mendocina ymp | 2 | YP_001188069.1 | 1 | 2846442-2847020 | 191 | 12.05 | 2217515-2217576 |
|  |  |  | YP_001190076.1 | 2 | 5045922-5046521 |  |  |  |
| NC_009445.1 | Bradyrhizobium sp. ORS278 | 4 | YP_001205785 | 1 | 3962200-3962790 | 257 | 11.25 | 6579310-6579358 |
|  |  |  | YP_001204712.1 | 2 | 2859670-2860293 |  |  |  |
|  |  |  | YP_001206792.1 | 2 | 5063404-5064069 |  |  |  |
|  |  |  | YP_001207756.1 | 2 | 6094322-6094930 |  |  |  |
| NC_009485.1 | Bradyrhizobium sp. BTAi1 | 4 | YP_001240107.1 | 1 | 4346181-4346771 | 73 | 14.77 | 6902858-6902810 |
|  |  |  | YP_001238051.1 | 2 | 2017601-2018047 | 170 | 12.28 | 4915593-4915541 |
|  |  |  | YP_001239025.1 | 2 | 3130103-3130744 |  |  |  |
|  |  |  | YP_001239219.1 | 2 | 3351729-3352394 |  |  |  |
| NC_009633.1 | Alkaliphilus metalliredigens QYMF | 2 |  | 1 | 291290-291859 | 14 | 39.84 | 290271-290331 |
|  |  |  |  | 1 | 3443516-3444088 | 25 | 28.82 | 293287-293341 |
|  |  |  |  |  |  | 56 | 16.04 | 3519585-3519642 |
|  |  |  |  |  |  | 111 | 13.6 | 2545831-2545893 |
|  |  |  |  |  |  | 120 | 13.33 | 914169-914220 |
|  |  |  |  |  |  | 202 | 11.93 | 747949-747895 |
|  |  |  |  |  |  | 295 | 10.89 | 3445688-3445635 |
|  |  |  |  |  |  | 341 | 10.47 | 3606681-3606626 |
|  |  |  |  |  |  | 399 | 10.02 | 3210506-3210452 |
| NC_009648.1 | Klebsiella pneumoniae subsp. pneumoniae MGH 78578 | 1 | YP_001335880.1 | 4 | 2442223-2443404 | 90 | 14.14 | 4401158-4401213 |
| NC_009654.1 | Marinomonas sp. MWYL1 | 1 | YP_001341668.1 | 1 | 3197701-3198375 | 36 | 19.29 | 3196287-3196231 |
|  |  |  |  |  |  | 44 | 17.1 | 4586404-4586468 |
|  |  |  |  |  |  | 89 | 14.16 | 3266578-3266637 |
|  |  |  |  |  |  | 178 | 12.19 | 397442-397492 |
|  |  |  |  |  |  | 215 | 11.74 | 4907313-4907378 |
|  |  |  |  |  |  | 226 | 11.66 | 3246922-3246868 |
|  |  |  |  |  |  | 271 | 11.12 | 3519147-3519088 |
|  |  |  |  |  |  | 297 | 10.87 | 4205235-4205175 |
|  |  |  |  |  |  | 378 | 10.19 | 5040125-5040074 |
| NC_009720.1 | Xanthobacter autotrophicus Py2 | 2 | YP_001415993 | 1 | 1250110-1250718 |  |  |  |
|  |  |  | YP_001417405.1 | 1 | 2801223-2801888 |  |  |  |
| NC_009792.1 | Citrobacter koseri ATCC BAA-895 | 1 | YP_001452865.1 | 4 | 1253581-1254777 | 126 | 13.19 | 4243606-4243658 |
| NC_009832.1 | Serratia proteamaculans 568 | 1 | YP_001479139.1 | 4 | 3202640-3203920 |  |  |  |
| NC_009972.1 | Herpetosiphon aurantiacus ATCC 23779 | 2 | YP_001546963.1 | 1 | 5357769-5358356 | 86 | 14.27 | 5354031-5353982 |
|  |  |  | YP_001547117.1 | 1 | 5565358-5565954 | 137 | 12.97 | 2941344-2941396 |
|  |  |  |  |  |  | 141 | 12.83 | 1051162-1051113 |
|  |  |  |  |  |  | 183 | 12.13 | 6013611-6013559 |
|  |  |  |  |  |  | 188 | 12.08 | 6041559-6041616 |
|  |  |  |  |  |  | 350 | 10.41 | 5840194-5840237 |
| NC_010001.1 | Clostridium phytofermentans ISDg | 1 | YP_001557755.1 | 2 | 817143-817685 | 197 | 12.03 | 4467323-4467260 |
|  |  |  |  |  |  | 284 | 10.96 | 3828409-3828358 |
|  |  |  |  |  |  | 293 | 10.91 | 4756130-4756080 |
|  |  |  |  |  |  | 314 | 10.72 | 9120-9167 |
| NC_007958.1 | Rhodopseudomonas palustris BisB5 | 1 | YP_571414.1 | 1 | 4758443-4759027 |  |  |  |
| NC_007925.1 | Rhodopseudomonas palustris BisB18 | 2 | YP_531093.1 | 1 | 1326091-1326675 |  |  |  |
|  |  |  | YP_532885.1 | 2 | 3317711-3318376 |  |  |  |
| NC_007778.1. | Rhodopseudomonas palustris HaA2 | 1 | YP_485381.1 | 1 | 2016085-2016669 |  |  |  |
| NC_009937.1. | Azorhizobium caulinodans ORS 571 | 2 | YP_001523608.1 | 1 | 766094-766738 |  |  |  |
|  |  |  | YP_001526806 | 2 | 4441784-4442449 |  |  |  |
| NC_007802.1. | Jannaschia sp. CCS1 | 1 | YP_510980.1 | 1 | 3064221-3064841 |  |  |  |
| NC_008343.1. | Granulibacter bethesdensis CGDNIH1 | 1 | YP_745551.1 | 1 | 1947477-1948115 |  |  |  |
| NC_007973.1. | Ralstonia metallidurans CH34 | 1 | YP_582433 | 1 | 294684-295343 |  |  |  |
| NC_003295.1 | Ralstonia solanacearum GMI1000 | 1 | NP_518503.1 | 1 | 411163-411750 |  |  |  |
| NC_008061.1. | Burkholderia cenocepacia AU 1054 | 2 | YP_623723.1 | 1 | 1015190-1015849 |  |  |  |
| NC_008062.1. |  |  | YP_625597.1 | 4 | 298388-299743 |  |  |  |
| NC_008391.1. | Burkholderia cepacia AMMD (ambiferia) | 1 | YP_775828.1 | 1 | 835321-835917 |  |  |  |
| NC_007511.1. | Burkholderia sp. 383 | 2 | YP_372098.1 | 1 | 1523077-1523673 |  |  |  |
|  |  |  | YP_372605.1 | 2 | 2113715-2114326 |  |  |  |
| NC_008825.1. | Methylibium petroleiphilum PM1 | 3 | NC_008825.1. | 1 | 1503429-1504061 |  |  |  |
|  |  |  | YP_001022049.1 | 2 | 3047913-3048509 |  |  |  |
|  |  |  | YP_001021505.1 | 2 | 2478938-2480218 |  |  |  |
| NC_008781.1. | Polaromonas naphthalenivorans CJ2 | 1 | YP_981650.1 | 1 | 1502889-1503521 |  |  |  |
| NC_008740.1. | Marinobacter aquaeolei VT8 | 1 | YP_957468.1 | 4 | 209370-210668 |  |  |  |
| NC_003919.1. | Xanthomonas axonopodis pv. citri str. 306 | 1 | NP_642474.1 | 11 | 2527505-2529043 |  |  |  |
| NC_009434.1. | Pseudomonas stutzeri A1501 | 2 | YP_001172895.1 | 1 | 2624343-2624969 |  |  |  |
|  |  |  | YP_001174556.1 | 4 | 4444490-4445791 |  |  |  |
| NC_008278.1. | Frankia alni ACN14a | 7 | YP_714804.1 | 1 | 5016807-5017406 |  |  |  |
|  |  |  | YP_716178.1 | 2 | 6549000-6550751 |  |  |  |
|  |  |  | YP_714088.1 | 2 | 4175339-4176058 |  |  |  |
|  |  |  | YP_713521.1 | 2 | 3596297-3597049 |  |  |  |
|  |  |  | YP_716747.1 | 3 | 7204178-7204882 |  |  |  |
|  |  |  | YP_714087.1 | 3 | 4174572-4175342 |  |  |  |
|  |  |  | YP_716589.1 | 6 | 7023663-7026245 |  |  |  |
| NC_003155.4. | Streptomyces avermitilis MA-4680 | 9 | NP_827395.1 | 1 | 7471444-7472100 |  |  |  |
|  |  |  | NP_822709 | 2 | 1877473-1877823 |  |  |  |
|  |  |  | NP_823234.1 | 2 | 2507392-2508177 |  |  |  |
|  |  |  | NP_825806 | 2 | 5653610-5654485 |  |  |  |
|  |  |  | NP_825808.1 | 2 | 5655894-5656178 |  |  |  |
|  |  |  | NP_828577.1 | 2 | 8830427-8830816 |  |  |  |
|  |  |  | NP_822230.1 | 3 | 1332766-1333503 |  |  |  |
|  |  |  | NP_825805.1 | 3 | 5652810-5653526 |  |  |  |
|  |  |  | NP_822261.1 | 6 | 1372393-1374891 |  |  |  |
| NC_008596.1. | Mycobacterium smegmatis str. MC2 155 | 4 | YP_887557.1 | 1 | 3328852-3329478 |  |  |  |
|  |  |  | YP_889790.1 | 2 | 5643478-5644194 |  |  |  |
|  |  |  | YP_890949.1 | 2 | 6787549-6788229 |  |  |  |
|  |  |  | YP_884953.1 | 5 | 620034-620738 |  |  |  |
| NC_006361.1. | Nocardia farcinica IFM 10152 | 8 | YP_118080.1 | 1 | 2040643-2041257 |  |  |  |
|  |  |  | YP_118238.1 | 5 | 2209775-2210437 |  |  |  |
|  |  |  | YP_122133.1 | 2 | 76111-76500 |  |  |  |
|  |  |  | YP_120347.1 | 3 | 4281361-4282083 |  |  |  |
|  |  |  | YP_120596.1 | 5 | 4543613-4544401 |  |  |  |
|  |  |  | YP_116504.1 | 3 | 312539-313309 |  |  |  |
|  |  |  | YP_118920.1 | 5 | 2874607-2875287 |  |  |  |
|  |  |  | YP_116365.1 | 5 | 147579-148289 |  |  |  |
| NC_008268.1. | Rhodococcus sp. RHA1 | 19 | YP_700984.1 | 1 | 1062747-1063367 |  |  |  |
|  |  |  | YP_702470.1 | 2 | 2660126-2660410 |  |  |  |
|  |  |  | YP_707557.1 | 2 | 334607-335323 |  |  |  |
|  |  |  | YP_700104.1 | 2 | 11753-112478 |  |  |  |
|  |  |  | YP_700429.1 | 2 | 516273-516647 |  |  |  |
|  |  |  | YP_707600.1 | 2 | 372118-372693 |  |  |  |
|  |  |  | YP_703376.1 | 2 | 3606238-3606975 |  |  |  |
|  |  |  | YP_701271.1 | 2 | 1359454-1360311 |  |  |  |
|  |  |  | YP_707630.1 | 2 | 401094-401408 |  |  |  |
|  |  |  | YP_700159.1 | 2 | 224570-224917 |  |  |  |
|  |  |  | YP_700089.1 | 2 | 101287-102057 |  |  |  |
|  |  |  | YP_700325.1 | 2 | 400312-400638 |  |  |  |
|  |  |  | YP_702081.1 | 3 | 2223532-2224296 |  |  |  |
|  |  |  | YP_707543.1 | 3 | 322533-323312 |  |  |  |
|  |  |  | YP_707969.1 | 3 | 740226-740873 |  |  |  |
|  |  |  | YP_703524.1 | 3 | 3768889-3769494 |  |  |  |
|  |  |  | YP_703365.1 | 3 | 3597873-3598556 |  |  |  |
|  |  |  | YP_700679.1 | 3 | 743900-744553 |  |  |  |
|  |  |  | YP_700147.1 | 3 | 211285-211956 |  |  |  |
| NC_008699.1 | Nocardioides sp. JS614 | 2 | YP_924207.1 | 1 | 3215009-3215632 |  |  |  |
|  |  |  | YP_923121.1 | 3 | 2051292-2052038 |  |  |  |
| **Genome accession #** | **ORGANISM NAME** | **# of ANTARS^a^** | **ANTAR protein accession #** | **ANTAR archi-tecture^b^** | **ANTAR protein positions** | **RNA hit #^c^** | **RNA hit Score^d^** | **RNA hit positions** |
|  |  |  |  |  |  |  |  |  |
| ***eut*-Containing Organisms without Putative ANTAR Domain Proteins** | | | | | | | | |
| NC_000913.2 | Escherichia coli str. K-12 substr. MG1655 | 0 |  |  |  | 233 | 11.53 | 1550757-1550698 |
|  |  |  |  |  |  | 291 | 10.91 | 1424328-1424385 |
| NC_003197.1 | Salmonella enterica subsp. enterica serovar Typhimurium str. LT2 | 0 |  |  |  | 52 | 16.13 | 3479948-3480000 |
|  |  |  |  |  |  | 380 | 10.17 | 3773383-3773333 |
| NC_003198.1 | Salmonella enterica subsp. enterica serovar Typhi str. CT18 | 0 |  |  |  | 53 | 16.13 | 3334474-3334526 |
|  |  |  |  |  |  | 163 | 12.37 | 2954650-2954705 |
| NC_005126.1 | Photorhabdus luminescens subsp. laumondii TTO1 | 0 |  |  |  | 138 | 12.94 | 3645746-3645807 |
|  |  |  |  |  |  | 143 | 12.78 | 1835848-1835785 |
|  |  |  |  |  |  | 388 | 10.1 | 1867505-1867444 |
| NC_007384.1 | Shigella sonnei Ss046 | 0 |  |  |  | 217 | 11.72 | 1739012-1739071 |
| NC_007606.1 | Shigella dysenteriae Sd197 | 0 |  |  |  | 151 | 12.65 | 3595644-3595698 |
|  |  |  |  |  |  | 272 | 11.13 | 1470062-1470003 |
| NC_007613.1 | Shigella boydii Sb227 | 0 |  |  |  | 218 | 11.72 | 1574176-1574235 |
| NC_007643.1 | Rhodospirillum rubrum ATCC 11170 | 0 |  |  |  | 224 | 11.67 | 2914531-2914583 |
| NC_008255.1 | Cytophaga hutchinsonii ATCC 33406 | 0 |  |  |  | 78 | 14.49 | 3523741-3523812 |
|  |  |  |  |  |  | 127 | 13.17 | 1241209-1241266 |
|  |  |  |  |  |  | 237 | 11.47 | 677200-677255 |
|  |  |  |  |  |  | 340 | 10.47 | 1123672-1123615 |
| NC_008536.1 | Candidatus Solibacter usitatus Ellin6076 | 0 |  |  |  | 0 |  |  |
| NC_008709.1 | Psychromonas ingrahamii 37 | 0 |  |  |  | 41 | 18.7 | 3591697-3591757 |
|  |  |  |  |  |  | 225 | 11.67 | 1588101-1588058 |
|  |  |  |  |  |  | 265 | 11.2 | 4244868-4244934 |
|  |  |  |  |  |  | 329 | 10.57 | 2987220-2987279 |
| NC_009922.1 | Alkaliphilus oremlandii OhILAs | 0 |  |  |  | 87 | 14.25 | 35107-35156 |
|  |  |  |  |  |  | 266 | 11.19 | 513543-513596 |
|  |  |  |  |  |  | 276 | 11.03 | 2670896-2670848 |
|  |  |  |  |  |  | 282 | 10.96 | 1098793-1098855 |
|  |  |  |  |  |  | 352 | 10.4 | 233808-233864 |
|  |  |  |  |  |  | 368 | 10.24 | 1618710-1618763 |
|  |  |  |  |  |  | 394 | 10.06 | 1019131-1019178 |
| not searched | Myxococcus xanthus DK 1622 | 0 |  |  |  |  |  |  |
| NC_009348.1 | Aeromonas salmonicida subsp. salmonicida A449 | 0 |  |  |  |  |  |  |
