# Supplementary material for: The Mechanism for RNA Recognition by ANTAR Regulators of Gene Expression
Source: PLoS Genet. 2012 Jun 7;8(6):e1002666. doi: 10.1371/journal.pgen.1002666 (PMC3369931; doi:10.1371/journal.pgen.1002666)
Supplement: Table S2 — Covariance Search Results for Discovery of ANTAR Substrates that Overlap Terminator Hairpins in Diverse Bacteria. (DOCX) [file pgen.1002666.s006.docx]

**Table S2. Covariance Search Results for Discovery of ANTAR Substrates that Overlap Terminator Hairpins in Diverse Bacteria**

^a^RNA Hit Coordinates in this Table specifically refer to the coordinates for the putative ANTAR target RNA hit as recovered by the Infernal, and that were then predicted to overlap with a putative intrinsic transcription terminator, as predicted by TransTermHP software. Since inclusion of an intrinsic transcription terminator was not part of the original search criteria, the observation that all of the RNA hits in this table overlap with terminator hairpins confers to them a high overall degree of confidence. In general, these RNA hits represent the best possible candidates for ANTAR-based regulation of downstream gene expression via transcription attenuation.

| **Genome accession #** | **ORGANISM NAME** | **ANTAR RNA hit coordinates^a^** | **predicted functions of downstream genes** |
| --- | --- | --- | --- |
| **NC_015278.1** | **Aerococcus urinae** | 1548052-1547999 | **Signal recognition particle components; YlxM family protein** |
|  | | | |
| **NC_015183.1** | **Agrobacterium sp.** | 1712102-1712155 | **AGROH133_06740 AraC family regulatory protein** |
|  | | | |
| **NC_008260.1** | **Alcanivorax borkumensis** | 947706-947765 | **Nitrate transporter (nrtC-nrtB-nasD); hypothetical proteins; nitrite reductase (nirB)** |
|  | | | |
| **NC_009633.1** | **Alkaliphilus metalliredigens** | 290277-290331 | **Propanediol/ethanolamine utilization pathway, including ANTAR regulatory protein** |
|  |  | 293288-293342 | **Propanediol/ethanolamine utilization pathway, including ANTAR regulatory protein** |
|  |  | 3445690-3445635 | **Glutamine synthetase (Amet_3386); ANTAR regulatory protein (Amet_3385); Note: the RNA hit is just downstream of gatB (aspartyl/glutamyl-tRNA amidotransferase)** |
|  | | | |
| **NC_011138.2** | **Alteromonas macleodii** | 3604944-3604889 | **None – the RNA hit is convergently arranged with the downstream gene, which encodes for a GntR-family transcriptional regulator** |
|  |  | 1124985-1125061 | **Uncharacterized GTP-binding protein** |
|  | | | |
| **NC_014817.1** | **Asticcacaulis excentricus** | 3445690-3445635 | **Hypothetical protein; Note: immediately downstream of methionyl-tRNA synthetase** |
|  | | | |
| **NC_009937.1** | **Azorhizobium caulinodans** | 759053-759007 | **Nitrate transporter; hemoprotein; nitrite reductase; ferredoxin-nitrite reductase** |
|  | | | |
| **NC_011835.1** | **Bifidobacterium animalis subsp. lactis** | 240097-240028 | **Hypothetical protein; polyphosphate kinase; MutT1 protein homologue** |
|  | | | |
| **NC_012491.1** | **Brevibacillus brevis** | 995676-995620 | **Uncharacterized transporter (BBR47_09480)** |
|  |  | 5933516-5933465 | **Ethanolamine ammonia lyase genes; Note: the RNA hit is just downstream of the ANTAR-containing response regulator, AmiR** |
|  | | | |
| **NC_014387.1** | **Butyrivibrio proteoclasticus** | 2692602-2692550 | **Hypothetical proteins** |
|  | | | |
| **NC_014720.1** | **Caldicellulosiruptor kronotskyensis** | 440298-440363 | **Methyl-accepting chemotaxis sensory transducer protein; hypothetical proteins** |
|  | | | |
| **NC_014721.1** | **Caldicellulosiruptor kristjanssonii** | 1841014-1840962 | **Unknown ligand-binding receptor; aconitate hydratase; isocitrate dehydrogenase; GntR family regulator; Note: RNA hit located just downstream of pyruvate carboxyltransferase** |
|  | | | |
| **NC_015949.1** | **Caldicellulosiruptor lactoaceticus** | 1243166-1243115 | **Unknown ligand-binding receptor; aconitate hydratase; isocitrate dehydrogenase; GntR family regulator; Note: RNA hit located just downstream of pyruvate carboxyltransferase** |
|  | | | |
| **NC_014657.1** | **Caldicellulosiruptor owensensis** | 603347-603391 | **Glutamine synthetase** |
|  | | | |
| **NC_009437.1** | **Caldicellulosiruptor saccharolyticus** | 2878839-2878888 | **The RNA hit is located just downstream of GCN5-like N-acetyltransferase, but is arranged in antisense direction with respect to convergently facing downstream genes** |
|  | | | |
| **NC_010424.1** | **Candidatus Desulforudis** | 2134699-2134645 | **Glutamine synthetase; glutamine amidotransferase; glutamate synthetase; Note: the RNA hit is located downstream of an ANTAR-containing response regulator gene (Daud_2028)** |
|  |  | 2135521-2135468 | **ANTAR-containing response regulator gene (Daud_2028); Glutamine synthetase; glutamine amidotransferase; glutamate synthetase** |
|  | | | |
| **NC_007503.1** | **Carboxydothermus hydrogenoformans** | 617722-617782 | **ANTAR-containing response regulator protein; glutamine synthetase; diguanylate cyclase; glutamate synthase-like protein** |
|  |  | 621874-621927 | **Hypothetical protein; glutamate synthase-like protein** |
|  | | | |
| **NC_003030.1** | **Clostridium acetobutylicum** | 2844754-2844711 | **Ethanolamine permease; ethanolamine ammonia-lyase** |
|  | | | |
| **NC_009617.1** | **Clostridium beijerinckii** | 2338246-2338298 | **Homocitrate synthase; isopropylmalate-homocitrate-citramalate synthase-like protein; Note: RNA hit is just downstream of two AsnC-family regulatory genes (Cbei_2009, Cbei_2010)** |
|  |  | 3219737-3219686 | **Extracellular ligand-binding receptor; Hypothetical proteins; Unknown transporter; AsnC-family regulator gene (Cbei_2763)** |
|  | | | |
| **NC_009699.1** | **Clostridium botulinum** | 1992628-1992561 | **Uncharacterized transport gene** |
|  |  | 1080527-1080572 | **Hypothetical protein** |
|  | | | |
| **NC_014393.1** | **Clostridium cellulovorans** | 3732553-3732514 | **Hypothetical protein; phosphoribosyltransferase** |
|  | | | |
| **NC_009089.1** | **Clostridium difficile** | 2209708-2209768 | **Ethanolamine/propanediol utilization genes; Two-component sensor kinase and ANTAR-containing response regulator gene** |
|  |  | 2208388-2208436 | **Ethanolamine/propanediol utilization genes; Two-component sensor kinase and ANTAR-containing response regulator gene** |
|  | | | |
| **NC_013315.1** | **Clostridium difficile** | 2062509-2062569 | **Ethanolamine/propanediol utilization genes; Two-component sensor kinase and ANTAR-containing response regulator gene** |
|  |  | 2061189-2061237 | **Ethanolamine/propanediol utilization genes; Two-component sensor kinase and ANTAR-containing response regulator gene** |
|  | | | |
| **NC_013316.1** | **Clostridium difficile** | 2143061-2143121 | **Ethanolamine/propanediol utilization genes; Two-component sensor kinase and ANTAR-containing response regulator gene** |
|  |  | 2141741-2141789 | **Ethanolamine/propanediol utilization genes; Two-component sensor kinase and ANTAR-containing response regulator gene** |
|  | | | |
| **NC_015275.1** | **Clostridium lentocellum** | 1418174-1418226 | **Glutamine permease (Clole_1236); Uncharacterized transporter** |
|  |  | 4679823-4679776 | **Aspartyl/glutamyl-tRNA amidotransferase; aspartyl-tRNA synthetase; glutamine synthetase; Note: RNA hit is just downstream of glutamate synthase subunit** |
|  | | | |
| **NC_003366.1** | **Clostridium perfringens** | 1095500-1095554 | **Ethanolamine/propanediol utilization genes; Two-component sensor kinase and ANTAR-containing response regulator gene** |
|  |  | 1093954-1094002 | **Ethanolamine/propanediol utilization genes; Two-component sensor kinase and ANTAR-containing response regulator gene** |
|  | | | |
| **NC_008261.1** | **Clostridium perfringens** | 1052252-1052306 | **Ethanolamine/propanediol utilization genes; Two-component sensor kinase and ANTAR-containing response regulator gene** |
|  |  | 1050706-1050754 | **Ethanolamine/propanediol utilization genes; Two-component sensor kinase and ANTAR-containing response regulator gene** |
|  | | | |
| **NC_010001.1** | **Clostridium phytofermentans** | 4756129-4756080 | **Uncharacterized genes** |
|  | | | |
| **NC_014376.1** | **Clostridium saccharolyticum** | 758961-759011 | **Orn/DAP/Arg decarboxylase (Closa_0671) [LysA homologue]** |
|  | | | |
| **NC_015737.1** | **Clostridium sp.** | 396480-396526 | **Asparagine synthase** |
|  | | | |
| **NC_014614.1** | **Clostridium sticklandii** | 289243-289314 | **Ethanolamine/propanediol utilization genes; Two-component sensor kinase and ANTAR-containing response regulator gene** |
|  | | | |
| **NC_004557.1** | **Clostridium tetani** | 2307822-2307769 | **Ethanolamine/propanediol utilization genes; Two-component sensor kinase and ANTAR-containing response regulator gene** |
|  |  | 2309186-2309132 | **Ethanolamine/propanediol utilization genes; Two-component sensor kinase and ANTAR-containing response regulator gene** |
|  | | | |
| **NC_009012.1** | **Clostridium thermocellum** | 1820473-1820517 | **Ammonium transporter (Cthe_1499)** |
|  | | | |
| **NC_003910.7** | **Colwellia psychrerythraea** | 3440717-3440671 | **Nitrate transporter genes; Note: RNA hit located downstream of an ANTAR-containing NasT homologue (CPS_3317)** |
|  | | | |
| **NC_015914.1** | **Cyclobacterium marinum** | 2628246-2628203 | **None – The RNA hit is arranged in an antisense direction to a convergently facing gene, Cycma_2259)** |
|  | | | |
| **NC_007907.1** | **Desulfitobacterium hafniense** | 4989850-4989799 | **Uncharacterized gene with homology to glutamate synthase** |
|  |  | 5011988-5011936 | **Glutamine synthetase; Note: RNA hit is located downstream of a putative reductase for nitro-containing compounds (DSY4406)** |
|  |  | 4845217-4845167 | **Hypothetical proteins** |
|  | | | |
| **NC_011830.1** | **Desulfitobacterium hafniense** | 1013599-1013654 | **Glutamine synthetase; Note: RNA hits is located downstream of a nitroreductase** |
|  |  | 1037358-1037409 | **Glutamate synthase** |
|  |  | 1158795-1158845 | **Methyl-accepting chemotaxis sensory transducer; nitrogen regulatory protein P-II; C4-dicarboxylate ABC transporter** |
|  | | | |
| **NC_013216.1** | **Desulfotomaculum acetoxidans** | 1038893-1038939 | **Ammonium transporter; nitrogen regulatory protein P-II** |
|  |  | 1656943-1656990 | **XRE-family transcriptional regulator** |
|  |  | 34596-34642 | **Ammonium transporter; nitrogen regulatory protein P-II** |
|  |  | 1043969-1044020 | **Pyruvate carboxyltransferase; nitrogenase subunits; nitrogen regulatory protein P-II** |
|  |  | 24144-24194 | **Porphyrin biosynthesis genes; methyltransferase cognate corrinoid protein (Dtox_0024); monomethylamine methyltransferase (Dtox_0025)** |
|  |  | 1040334-1040409 | **Ammonium transporter; nitrogen regulatory protein P-II** |
|  |  | 1042306-1042369 | **Hypothetical protein** |
|  |  | 1043787-1043837 | **Pyruvate carboxyltransferase; nitrogenase subunits; nitrogen regulatory protein P-II** |
|  |  | 1656692-1656742 | **XRE-family transcriptional regulator** |
|  |  | 4184535-4184578 | **None – The RNA hit is arranged in an antisense direction to a convergently facing gene encoding for a Glu/Leu/Phe/Val dehydrogenase (Dtox_3997); however, the RNA hit is located downstream of a glutamine synthetase** |
|  |  | 34402-34448 | **Ammonium transporter; nitrogen regulatory protein P-II** |
|  |  | 3931476-3931418 | **ANTAR-containing response regulator; Note: the RNA hit is located downstream of a glutamate synthase-like protein** |
|  |  | 3930782-3930730 | **glutamine synthetase** |
|  | | | |
| **NC_015565.1** | **Desulfotomaculum carboxydivorans** | 555381-555432 | **Glutamine synthetase; Note: the RNA hit is downstream of an ANTAR-containing response regulator** |
|  |  | 564955-565000 | **Ammonium transporter; nitrogen regulatory protein P-II** |
|  |  | 554696-554748 | **ANTAR-containing response regulator** |
|  |  | 813992-814050 | **Urea transporter genes; urease subunits** |
|  |  | 556968-557026 | **Glutamine synthetase; glutamate synthase** |
|  |  | 2564555-2564604 | **Amino acid carrier protein (Desca_2441)** |
|  |  | 1250720-1250771 | **Nitrogenase subunits; two copies of nitrogen regulatory protein P-II** |
|  |  | 813893-813941 | **Urea transporter genes; urease subunits** |
|  |  | 569777-569844 | **Uncharacterized NAD+ synthetase** |
|  | | | |
| **NC_015573.1** | **Desulfotomaculum kuznetsovii** | 546955-547011 | **Glutamine synthetase** |
|  |  | 546214-546271 | **ANTAR-containing response regulator** |
|  |  | 548562-548614 | **Glutamine synthetase; nitrite reductase** |
|  |  | 3376145-3376088 | **Ammonium transporter; nitrogen regulatory P-Ii; nitrogenase subunits** |
|  | | | |
| **NC_009253.1** | **Desulfotomaculum reducens** | 45161-45211 | **None – the RNA hit is arranged downstream of a DEAD/DEAH box helicase and in an antisense arrangement with convergently facing genes** |
|  |  | 3046890-3046842 | **Glutamine synthetase** |
|  |  | 3047579-3047528 | **ANTAR-containing response regulator** |
|  |  | 3045304-3045244 | **Glutamine synthetase; glutamate synthase** |
|  |  | 3108830-3108783 | **Ammonium transporter; nitrogen regulatory protein P-II** |
|  | | | |
| **NC_012880.1** | **Dickeya dadantii** | 917834-917753 | **Subunits for uncharacterized transporter, which might transport urea; acetamidase/formamidase; ANTAR-containing regulatory protein** |
|  | | | |
| **NC_012912.1** | **Dickeya zaee** | 1639521-1639613 | **Nitrate transport proteins; nitrite reductase; Note: the RNA hit is located downstream of an ANTAR-containing regulatory protein** |
|  | | | |
| **NC_004668.1** | **Enterococcus faecalis** | 1590876-1590821 | **Ethanolamine utilization pathway** |
|  |  | 1592373-1592320 | **Ethanolamine utilization pathway** |
|  |  | 1587123-1587070 | **Ethanolamine utilization pathway** |
|  |  | 3055748-3055699 | **Hypothetical protein** |
|  | | | |
| **NC_014828.1** | **Ethanoligenens harbinense** | 2593351-2593414 | **Nitrogen regulatory protein P-II** |
|  | | | |
| **NC_012780.1** | **Eubacterium eligens** | 528763-528710 | **Hypothetical proteins; Note: RNA hit is located just downstream of a putative spermidine/putrescine transporter** |
|  | | | |
| **NC_003454.1** | **Fusobacterium nucleatum ATCC 25586** | 716227-716317 | **Ethanolamine utilization pathway; Note: the RNA hit is located just downstream of an ANTAR-containing response regulator** |
|  | | | |
| **NC_011146.1** | **Geobacter bemidjiensis** | 1326892-1326945 | **Hypothetical protein** |
|  |  | 2408198-2408262 | **Radical SAM protein (Gbem2083); Uncharacterized GNAT family acetyltransferase (Gbem_2084)** |
|  | | | |
| **NC_007517.1** | **Geobacter metallireducens** | 1099587-1099627 | **Fe-S cluster assembly protein NifU-like protein; cysteine desulfurase** |
|  | | | |
| **NC_011979.1** | **Geobacter sp.** | 2844177-2844233 | **Radical SAM protein (Geob_2591); N-acetyltransferase GCN5 (Geob_2592); methyl-accepting chemotaxis sensory transducer** |
|  |  | 3100909-3100964 | **None – the RNA hit is downstream of a hypothetical protein and arranged in an antisense direction with tRNA-Ala** |
|  |  | 2007634-2007688 | **Hypothetical protein; nitrogen regulatory protein P-II; ammonium transporter** |
|  | | | |
| **NC_012918.1** | **Geobacter sp.** | 2493611-2493547 | **Radical SAM protein; N-acetyltransferase GCN5** |
|  |  | 3643098-3643047 | **Hypothetical protein** |
|  |  | 2504735-2504674 | **Nitrogenase subunits** |
|  | | | |
| **NC_014973.1** | **Geobacter sp.** | 2254932-2254986 | **Radical SAM protein; N-acetyltransferase GCN5** |
|  |  | 2244144-2244203 | **Nitrogenase subunits** |
|  |  | 1208178-1208238 | **Hypothetical protein** |
|  | | | |
| **NC_002939.4** | **Geobacter sulfurreducens** | 1008412-1008483 | **Hypothetical protein; nitrogen regulatory protein P-II; ammonium transporter** |
|  | | | |
| **NC_009483.1** | **Geobacter uraniireducens** | 3952988-3952924 | **Hypothetical protein; nitrogen regulatory protein P-II; ammonium transporter** |
|  | | | |
| **NC_015497.1** | **Glaciecola sp.** | 2275751-2275809 | **Nitrate transport proteins** |
|  | | | |
| **NC_010337.2** | **Heliobacterium modesticaldum** | 2326506-2326551 | **Glutamine synthetase** |
|  | | | |
| **NC_009659.1** | **Janthinobacterium sp. Marseille** | 530479-530575 | **Nitrate transport proteins; nitrite reductase; Note: the RNA hit is located downstream of an ANTAR-containing response regulator** |
|  | | | |
| **NC_013192.1** | **Leptotrichia buccalis** | 84179-84132 | **Ethanolamine utilization pathway** |
|  | | | |
| **NC_016011.1** | **Listeria ivanovii** | 1157850-1157906 | **Ethanolamine utilization pathway** |
|  |  | 1154318-1154371 | **Ethanolamine utilization pathway** |
|  |  | 1155635-1155701 | **Ethanolamine utilization pathway** |
|  |  | 1192873-1192927 | **Putative N-acetylmuramoyl-L-alanine amidase (LIV_1151)** |
|  | | | |
| **NC_002973.6** | **Listeria monocytogenes** | 1177301-1177355 | **Ethanolamine utilization pathway** |
|  |  | 1180837-1180893 | **Ethanolamine utilization pathway** |
|  |  | 1178617-1178684 | **Ethanolamine utilization pathway** |
|  | | | |
| **NC_003210.1** | **Listeria monocytogenes** | 1198521-1198575 | **Ethanolamine utilization pathway** |
|  |  | 1202057-1202113 | **Ethanolamine utilization pathway** |
|  |  | 1199837-1199904 | **Ethanolamine utilization pathway** |
|  | | | |
| **NC_011660.1** | **Listeria monocytogenes** | 1496892-1496838 | **Ethanolamine utilization pathway** |
|  |  | 1493355-1493299 | **Ethanolamine utilization pathway** |
|  |  | 1495575-1495508 | **Ethanolamine utilization pathway** |
|  | | | |
| **NC_012488.1/** | **Listeria monocytogenes** | 1180861-1180915 | **Ethanolamine utilization pathway** |
|  |  | 1184397-1184453 | **Ethanolamine utilization pathway** |
|  |  | 1182177-1182244 | **Ethanolamine utilization pathway** |
|  | | | |
| **NC_013766.1** | **Listeria monocytogenes** | 1222813-1222867 | **Ethanolamine utilization pathway** |
|  |  | 1226349-1226405 | **Ethanolamine utilization pathway** |
|  |  | 1224129-1224196 | **Ethanolamine utilization pathway** |
|  | | | |
| **NC_013768.1** | **Listeria monocytogenes** | 1189517-1189571 | **Ethanolamine utilization pathway** |
|  |  | 1193053-1193109 | **Ethanolamine utilization pathway** |
|  |  | 1190833-1190900 | **Ethanolamine utilization pathway** |
|  | | | |
| **NC_013891.1** | **Listeria seeligeri** | 1082149-1082202 | **Ethanolamine utilization pathway** |
|  |  | 1085680-1085737 | **Ethanolamine utilization pathway** |
|  |  | 1083465-1083531 | **Ethanolamine utilization pathway** |
|  | | | |
| **NC_008555.1** | **Listeria welshimeri** | 1141577-1141629 | **Ethanolamine utilization pathway** |
|  |  | 1145115-1145166 | **Ethanolamine utilization pathway** |
|  |  | 1142890-1142957 | **Ethanolamine utilization pathway** |
|  | | | |
| **NC_010382.1** | **Lysinibacillus sphaericus** | 2163156-2163203 | **Ethanolamine utilization pathway** |
|  | | | |
| **NC_015520.1** | **Mahella australiensis** | 1564270-1564315 | **Ammonium transporter; nitrogen regulatory protein P-II** |
|  |  | 1561027-1561072 | **None** |
|  |  | 98136-98188 | **Ammonium transporter** |
|  |  | 2058149-2058089 | **Asparagine synthase** |
|  | | | |
| **NC_015559.1** | **Marinomonas posidonica** | 1345874-1345927 | **Nitrate/sulfonate/bicarbonate transporter subunits; nitrite reductase; Note: the RNA hit is downstream of a nitrate transporter gene as well** |
|  | | | |
| **NC_009654.1** | **Marinomonas sp.** | 3196287-3196231 | **Nitrate/sulfonate/bicarbonate transporter subunits; nitrite reductase; Note: the RNA hit is downstream of a nitrate transporter gene as well** |
|  | | | |
| **NC_007947.1** | **Methylobacillus flagellatus** | 478723-478663 | **Amidase operon; ANTAR-containing AmiR homologue** |
|  | | | |
| **NC_012968.1** | **Methylotenera mobilis** | 1808692-1808635 | **Nitrate transporter subunits; hypothetical protein; possibly upstream of nitrite reductase; Note: RNA hit is downstream of an ANTAR-containing response regulator** |
|  | | | |
| **NC_014207.1** | **Methylotenera versatilis** | 2935476-2935415 | **Nitrate transporter subunits; hypothetical protein; possibly upstream of nitrite reductase; Note: RNA hit is downstream of an ANTAR-containing response regulator** |
|  | | | |
| **NC_007644.1** | **Moorella thermoacetica** | 572089-572144 | **Nitrogen regulatory protein P-II; nitrogenase subunits** |
|  |  | 2415032-2414947 | **Glutamine synthetase** |
|  |  | 189904-189844 | **Ammonium transporter** |
|  |  | 1339331-1339279 | **Glutamine synthetase; Note: further downstream is an ANTAR-containing response regulator** |
|  | | | |
| **NC_010718.1** | **Natranaerobius thermophilus** | 1021194-1021246 | **Glutamine synthetase; ANTAR-containing response regulator; Note: RNA hit is downstream of an asparagine synthase** |
|  | | | |
| **NC_014483.1** | **Paenibacillus polymyxa** | 3250610-3250667 | **Nitrate/nitrite transporter** |
|  |  | 3243761-3243702 | **Nitrite reductase; assimilatory nitrite reductase subunit** |
|  |  | 3250452-3250401 | **Nitrite reductase; assimilatory nitrite reductase subunit; Note: the RNA hits at 3250610 and 3250452, while near to one another by proximity, are oriented in divergent directions.** |
|  | | | |
| **NC_014622.1** | **Paenibacillus polymyxa** | 3541006-3541063 | **Major facilitator superfamily mfs_1 (PPSC2_c3364)** |
|  |  | 3534277-3534221 | **Nitrite reductase** |
|  |  | 3540846-3540795 | **Nitrite reductase; Note: the RNA hits at 3541006 and 3540846 are close together but face in divergent directions.** |
|  | | | |
| **NC_012914.1** | **Paenibacillus sp.** | 2556611-2556666 | **Molybdenum cofactor synthesis protein; nitrite reductase; molybdopterin oxidoreductase family enzyme** |
|  |  | 6787424-6787363 | **Nitrite reductase** |
|  |  | 2556444-2556394 | **None; This RNA hit is arranged in a divergent direction with the hit at 2556611, and is within a 1.5 kb intergenic region.** |
|  | | | |
| **NC_013406.1** | **Paenibacillus sp.** | 2861369-2861425 | **Nitrite reductase; molybdopterin oxidoreductase family enzyme** |
|  |  | 2968741-2968795 | **Nitrite reductase genes; formate/nitrite transporter; uroporphyrin-III C-methyltransferase** |
|  |  | 2861209-2861146 | **MFS family transporter** |
|  | | | |
| **NC_016078.1** | **Pelagibacterium halotolerans** | 2325389-2325334 | **Nitrate/nitrite transporter; nitrite reductase subunits** |
|  | | | |
| **NC_007498.2** | **Pelobacter carbinolicus** | 2456544-2456594 | **MoaA/NifB/PqqE family nitrogen fixation protein; molybdenum transport system** |
|  |  | 2447465-2447532 | **Nitrogenase subunits** |
|  |  | 620590-620653 | **Nitrogenase-associated protein** |
|  | | | |
| **NC_009454.1** | **Pelotomaculum thermopropionicum** | 738375-738434 | **Glutamate synthase** |
|  |  | 651231-651284 | **Ammonia permease; nitrogen regulatory protein P-II** |
|  |  | 736857-736912 | **Glutamine synthetase; glutamate synthase** |
|  |  | 651312-651359 | **Ammonia permease; nitrogen regulatory protein P-II** |
|  | | | |
| **NC_008228.1** | **Pseudoalteromonas atlantica** | 2704551-2704607 | **Aldo/keto reductase family enzyme; tRNA/rRNA methyltransferase SpoU homologue** |
|  | | | |
| **NC_015977.1** | **Roseburia hominis** | 3208489-3208550 | **Hypothetical protein** |
|  | | | |
| **NC_009832.1** | **Serratia proteamaculans** | 2371242-2371181 | **Hypothetical protein** |
|  | | | |
| **NC_004347.1** | **Shewanella oneidensis** | 1647691-1647642 | **None – the RNA hit is oriented in a convergently facing antisense direction with an uncharacterized diguanylate syclase** |
|  | | | |
| **NC_009438.1** | **Shewanella putrefaciens** | 2598968-2598919 | **None – the RNA hit is oriented in a convergently facing direction with an uncharacterized sodiumdicarboxylate symporter** |
|  | | | |
| **NC_008750.1** | **Shewanella sp.** | 2017889-2017938 | **None – the RNA hit is oriented in a convergently facing direction with an uncharacterized sodiumdicarboxylate symporter** |
|  | | | |
| **NC_010506.1** | **Shewanella woodyi** | 2733738-2733794 | **Hypothetical proteins** |
|  | | | |
| **NC_012121.1** | **Staphylococcus carnosus** | 2432350-2432389 | **Succinate-semialdehyde dehrogenase; glutamine synthetase** |
|  | | | |
| **NC_009009.1** | **Streptococcus sanguinis** | 516637-516692 | **Ethanolamine utilization pathway** |
|  |  | 512877-512934 | **Ethanolamine utilization pathway** |
|  |  | 466895-466953 | **Cobalamin biosynthesis genes** |
|  | | | |
| **NC_015172.1** | **Syntrophobotulus glycolicus** | 769809-769864 | **Nitrogen regulatory protein P-II; transporter** |
|  |  | 2012120-2012069 | **Diguanylate cyclase/phosphodiesterase** |
|  | | | |
| **NC_014152.1** | **Thermincola potens** | 701428-701489 | **Uncharacterized transporter and hypothetical proteins** |
|  |  | 693969-694018 | **Nitrate transporter subunits** |
|  |  | 680425-680479 | **Nitrogenase subunits; nitrogen regulatory P-II** |
|  |  | 699073-699130 | **ANTAR-containing response regulator; glutamine synthetase** |
|  |  | 696066-696113 | **Hypothetical protein; Note: RNA hit is downstream of nitrogen regulatory protein and ammonium transporter** |
|  |  | 663177-663118 | **Asparagine synthase** |
|  |  | 2949025-2948971 | **Uncharacterized transporter subunits** |
|  | | | |
| **NC_014964.1** | **Thermoanaerobacter brockii** | 1052153-1052098 | **Orn/lys/arg decarboxylse** |
|  | | | |
| **NC_014831.1** | **Thermaerobacter marianensis** | 747617-747692 | **MerR family transcriptional regulator; glutamine synthetase; ANTAR-containing protein** |
|  |  | 685654-685702 | **Uncharacterized ABC transporter subunits; Note: the RNA hit is located downstream of an asparagine synthase** |
|  | | | |
| **NC_010320.1** | **Thermoanaerobacter sp** | 1471748-1471693 | **Orn/lys/arg decarboxylse** |
|  | | | |
| **NC_014538.1** | **Thermoanaerobacter sp.** | 1442635-1442690 | **Orn/lys/arg decarboxylse** |
|  | | | |
| **NC_010321.1** | **Thermoanaerobacter pseudethanolicus** | 1059960-1059905 | **Orn/lys/arg decarboxylse** |
|  | | | |
| **NC_014410.1** | **Thermoanaerobacter thermosaccharolyticum** | 1859180-1859073 | **Nitrogenase subunits; nitrogen regulatory protein P-II** |
|  |  | 1859388-1859491 | **Nitrogenase subunits; molybdenum transport proteins** |
|  |  | 1817738-1817642 | **Ammonium transport proteins; nitrogen regulatory protein P-II; asparagine synthase** |
|  | | | |
| **NC_015958.1** | **Thermoanaerobacter wiegelii** | 745558-745613 | **Ammonium transporter; nitrogen regulatory protein P-II; glutamine amidotransferase; glutamate synthase; 4Fe-4S ferredoxin** |
|  | | | |
| **NC_015555.1** | **Thermoanaerobacterium xylanolyticum** | 1081096-1081139 | **Ammonium transporter; nitrogen regulatory protein P-II; flagellar hook-associated protein; asparagine synthase** |
|  |  | 1699993-1699943 | **Glutamine synthetase; ANTAR-containing response regulator** |
|  | | | |
| **NC_015681.1** | **Thermodesulfatator indicus** | 542742-542694 | **Hypothetical protein; type IV pilus secretin** |
|  | | | |
| **NC_015499.1** | **Thermodesulfobium narugense** | 1728511-1728465 | **Nitrogen regulatory protein P-II; ammonium transporter** |
|  | | | |
| **NC_015581.1** | **Thioalkalimicrobium cyclicum** | 924026-924075 | **Nitrate/sulfonate/bicarbonate ABC transporter subunits; nitrite reductase** |
|  | | | |
| **NC_012691.1** | **Tolumonas auensis** | 1942176-1942104 | **Nitrate/sulfonate/bicarbonate ABC transporter subunits; Note: RNA hit is downstream of an ANTAR-containing protein gene** |
|  | | | |
| **NC_009784.1** | **Vibrio harveyi** | 791558-791636 | **Nitrate transport proteins; nitrate reductase** |
|  |  | 2430502-2430402 | **None – the RNA hit is located just downstream of a hypothetical protein and is arranged in an antisense orientation with a convergently facing hypothetical protein gene** |
|  | | | |
| **NC_004605.1** | **Vibrio parahaemolyticus** | 1117890-1117837 | **Nitrate transport genes; nitrite reductase; Note: RNA hit is downstream of an ANTAR-containing response regulator** |
|  | | | |
| **NC_013457.1** | **Vibrio sp.** | 1824865-1824918 | **Nitrate transport genes; nitrite reductase; Note: RNA hit is downstream of an ANTAR-containing response regulator** |
|  | | | |
| **NC_011753.2** | **Vibrio splendidus** | 2570476-2570407 | **Uncharacterized DNA-binding transcriptional activator; hypothetical proteins; RNA 2’-O-ribose methyltransferase enzyme (VS_2396)** |
|  | | | |
| **NC_005140.1** | **Vibrio vulnificus** | 1051524-1051447 | **Nitrate transport genes; nitrite reductase** |
|  | | | |
| **NC_008800.1** | **Yersinia enterocolitica** | 4157487-4157538 | **3-dehydroquinate dehydratase; acetyl-CoA carboxylase subunits** |
|  | | | |
| **NC_009720.1** | **Xanthobacter autotrophicus** | 2799847-2799798 | **Nitrate transport genes; nitrite reductase** |
|  |  |  |  |
