# Supplementary material for: The Mechanism for RNA Recognition by ANTAR Regulators of Gene Expression
Source: PLoS Genet. 2012 Jun 7;8(6):e1002666. doi: 10.1371/journal.pgen.1002666 (PMC3369931; doi:10.1371/journal.pgen.1002666)
Supplement: Table S3 — Strains, plasmids and primers used in this study. (DOCX) [file pgen.1002666.s007.docx]

**Table S3. Strains, plasmids and primers used in this study**

|  | **Description** | **Source** |
| --- | --- | --- |
| **Strains** |  |  |
| *E. faecalis* |  |  |
| OG1RF | Wild type strain, Fa^R^, Rf^R^ |  |
| AR2 | ∆*eutVW* , Fa^R^, Rf^R^ | Fox et.al. |
| SD46 | OG1RF pSD2, Fa^R^, Rf^R^, Em^R^ | This work |
| SD47 | ∆*eutVW* pSD2, Fa^R^, Rf^R^, Em^R^ | This work |
| SD48 | OG1RF pSD3, Fa^R^, Rf^R^, Em^R^ | This work |
| SD49 | ∆*eutVW*  pSD3, Fa^R^, Rf^R^, Em^R^ | This work |
| SD50 | OG1RF pSD4, Fa^R^, Rf^R^, Em^R^ | This work |
| SD51 | ∆*eutVW*  pSD4, Fa^R^, Rf^R^, Em^R^ | This work |
| SD52 | OG1RF pSD5, Fa^R^, Rf^R^, Em^R^ | This work |
| SD53 | ∆*eutVW* pSD5, Fa^R^, Rf^R^, Em^R^ | This work |
| SD92 | OG1RF pHFZ1, Fa^R^, Rf^R^, Em^R^ | This work |
| SD93 | ∆*eutVW* pHFZ1, Fa^R^, Rf^R^, Em^R^ | This work |
| SD97 | OG1RF pSD6, Fa^R^, Rf^R^, Em^R^ | This work |
| SD98 | ∆*eutVW*  pSD6, Fa^R^, Rf^R^, Em^R^ | This work |
| SD99 | OG1RF pSD7, Fa^R^, Rf^R^, Em^R^ | This work |
| SD100 | ∆*eutVW* pSD7, Fa^R^, Rf^R^, Em^R^ | This work |
| SD122 | OG1RF pSD8, Fa^R^, Rf^R^, Em^R^ | This work |
| SD123 | ∆*eutVW* pSD8, Fa^R^, Rf^R^, Em^R^ | This work |
| SD132 | OG1RF pHFZ2, Fa^R^, Rf^R^, Em^R^ | This work |
| SD133 | ∆*eutVW* pHFZ2, Fa^R^, Rf^R^, Em^R^ | This work |
| SD140 | OG1RF pSD9, Fa^R^, Rf^R^, Em^R^ | This work |
| SD141 | ∆*eutVW* pSD9, Fa^R^, Rf^R^, Em^R^ | This work |
| SD142 | OG1RF pSD10, Fa^R^, Rf^R^, Em^R^ | This work |
| SD143 | ∆*eutVW* pSD10, Fa^R^, Rf^R^, Em^R^ | This work |
| SD166 | OG1RF pSD11, Fa^R^, Rf^R^, Em^R^ | This work |
| SD167 | ∆*eutVW* pSD11, Fa^R^, Rf^R^, Em^R^ | This work |
| SD178 | OG1RF pSD12, Fa^R^, Rf^R^, Em^R^ | This work |
| SD179 | ∆*eutVW* pSD12, Fa^R^, Rf^R^, Em^R^ | This work |
| SD194 | OG1RF pSD13, Fa^R^, Rf^R^, Em^R^ | This work |
| SD195 | ∆*eutVW* pSD13, Fa^R^, Rf^R^, Em^R^ | This work |
| SD198 | OG1RF pSD14, Fa^R^, Rf^R^, Em^R^ | This work |
| SD199 | ∆*eutVW* pSD14, Fa^R^, Rf^R^, Em^R^ | This work |
| SD226 | OG1RF pSD15, Fa^R^, Rf^R^, Em^R^ | This work |
| SD227 | ∆*eutVW* pSD15, Fa^R^, Rf^R^, Em^R^ | This work |
| *E. coli* |  |  |
| TOP10 | Strain used for construction of pSD2-based plasmids |  |
| XL-1 Blue | Strain used for maintenance of pTOPO based site-directed mutagenesis clones |  |
|  |  |  |
| **Plasmids** |  |  |
| pUC18 | Source for amplicillin marker |  |
| pCJK47 | Source for *lacZ* |  |
| pCJK96 | Parent plasmid for construction of pCJK96-2 |  |
| pCJK96-2 | pCJK96 with rhamnose promoter deleted |  |
| pSD1 | pCJK96-2 with amplicillin marker | This work |
| pSD2 | pSD1 with *lacZ* from pCJK47 | This work |
| pKAF12 | pKAF6 *eutP* 5’ UTR |  |
| pSD3 | pSD2 *eutP* 5’ UTR | This work |
| pSD4 | pSD2 *eutP*∆T | This work |
| pSD5 | pSD2 *eutP* ∆P1 | This work |
| pSD6 | pSD2 *eutP* A29U | This work |
| pSD7 | pSD2 *eutP* G32A | This work |
| pSD8 | pSD2 *eutP* G51C | This work |
| pSD9 | pSD2 *eutP* A53U | This work |
| pSD10 | pSD2 *eutP* G56A | This work |
| pSD11 | pSD2 *eutP* A27U+C28G+G35C+U36A | This work |
| pSD12 | pSD2 *eutP* A25U+C26G+G37C+U38A | This work |
| pSD13 | pSD2 *eutP* A25U+C26G | This work |
| pSD14 | pSD2 *eutP* short linker |  |
| pSD15 | pSD2 *eutP* long linker |  |
| pHFZ1 | pSD2 *eutS* 5’ UTR | This work |
| pHFZ2 | pSD2 *eutS*∆T | This work |
|  |  |  |
| **Oligos** |  |  |
| SD1 | 5’ gcgtcgacGATATCCGTTTAGCGTTAACGCTAAG 3’ |  |
| SD2 | 5’ggatccTAGCTCCCATTAAAATGATTCGTTTCATCGCTACCTCCATCCTTTGCCGATTTGTTAAAAC 3’ |  |
| SD3 | 5’ggatccTAGCTCCCATTAAAATGATTCGTTTCATCGCTACCTCCATAAAAAAGGAGCCCAAAGCAAGTACCATCACGTACTTAGTCTTGGGCTCCTTTGCCGATTTGTTATCTGATTCTTTGCCTTTATCTTAC 3’ |  |
| SD6 | 5’gcggatccCACCGATAGCTCCCATTAAAATGATTCGTTTCATCGCTACCTCCAT 3’ |  |
| SD7 | 5’ GGCAAAGAATCAGAAACACTATGGCGTGTTTTAACAAATCGGC 3’ |  |
| SD8 | 5’ GCCGATTTGTTAAAACACGCCATAGTGTTTCTGATTCTTTGCC 3’ |  |
| SD9 | 5’ GGCAAAGAATCAGAAACACAATAGCGTGTTTTAACAAATCGGC 3’ |  |
| SD10 | 5’ GCCGATTTGTTAAAACACGCTATTGTGTTTCTGATTCTTTGCC 3’ |  |
| SD11 | 5’ CGTGTTTTAACAAATCGCCAAAGGAGCCCAAGACTAAG |  |
| SD12 | 5’ CTTAGTCTTGGGCTCCTTTGGCGATTTGTTAAAACACG 3’ |  |
| SD25 | 5 CGTGTTTTAACAAATCGGCTAAGGAGCCCAAGACTAAG 3’ |  |
| SD26 | 5’ CTTAGTCTTGGGCTCCTTAGCCGATTTGTTAAAACACG 3’ |  |
| SD27 | 5 GTGTTTTAACAAATCGGCAAAAGAGCCCAAGACTAAGTACG |  |
| SD28 | 5’ CGTACTTAGTCTTGGGCTCTTTTGCCGATTTGTTAAAACAC 3’ |  |
| SD29 | 5’ GGCAAAGAATCAGAAACTGAATGGCCAGTTTTAACAAATCGGCAAAGG 3’ |  |
| SD30 | 5’ CCTTTGCCGATTTGTTAAAACTGGCCATTCAGTTTCTGATTCTTTGCC 3’ |  |
| SD33 | 5’ GGCAAAGAATCAGAATGACAATGGCGTCATTTAACAAATCGGCAAAGG 3’ |  |
| SD34 | 5’ CCTTTGCCGATTTGTTAAATGACGCCATTGTCATTCTGATTCTTTGCC 3’ |  |
| SD35 | 5’ GGCAAAGAATCAGAATGACAATGGCGTGTTTTAACAAATCGGCAAAGG 3’ |  |
| SD36 | 5’ CCTTTGCCGATTTGTTAAAACACGCCATTGTCATTCTGATTCTTTGCC 3’ |  |
| SD 37 | 5’ CACAATGGCGTGTTTTATCGGCAAAGGAGCCCAAGAC 3’ |  |
| SD38 | 5’ GTCTTGGGCTCCTTTGCCGATAAAACACGCCATTGTG 3’ |  |
| SD41 | 5’ GCGGATCCGTTTCATCGCTACCTCCATAAAAAAGGAGCCCAAAGCAAGTACCATCACGTACTTAGTCTTGGGCTCCTTTGCCGGTATTCTTGCGTTAAAACACGC 3’ |  |
| SD42 | 5’ gcgtcgacCGACAAATCAAGCAACTCCGTCAA 3’ |  |
| SD43 | 5’ gcggatccTCATTCGTTGTTTTTCTTCCAACCAGACT 3’ |  |
| SD45 | 5’gcggatccTCATTCGTTGTTTTTCTTCCAACCAGACTCGCTCCTTTAATTTAGCAAATGCTTCTTTGCTCTTAAATTTTG 3’ |  |
| SD49 | 5’ GcggtaccgACGAAAGGGCCTCGTGATAC 3’ |  |
| SD50 | 5’ GcggtaccgCTCACGTTAAGGGATTTTGGTCATG 3’ |  |
| SD51 | 5’ gctctagaagatcttggccgtcgttttacaacgtc 3’ |  |
| SD52 | 5’ gcctcgagCCTCAACTCCAAATATCGTAG 3’ |  |
| AR064 | 5’TAATAcgactcactatagggcaaagaatcagaaacacaatgg 3’ |  |
| AR065 | 5’ AAAAAAGGAGCCCAAAGCAAGTACC 3’ |  |
| AR066 | 5’ cacgtacttagtcttgggctcc 3’ |  |
| AR067 | 5’ gccgatttgttaaaacacgc 3’ |  |
| AR151 | 5’ CACGTACTTAGTCTTGGG 3’ |  |
| AR150 | 5’CACGTACTTAGTCTTGGGCAAAAAAGCCGATTTGTTAAAACACAAAAAAGTGTTTCTGATTCTTTGCCCTATAGTGAGTCGTATTA 3’ |  |
| AR152 | 5’CACGTACTTAGTCTTGGGCTCCTAAGCCGATTTGTTAAAACACGCCAAAGTGTTTCTGATTCTTTGCCCTATAGTGAGTCGTATTA 3’ |  |
| AR153 | 5’CACGTACTTAGTCTTGGGCTAATTTGCCGATTTGTTAAAACACGAAATTGTGTTTCTGATTCTTTGCCCTATAGTGAGTCGTATTA 3’ |  |
| AR118 | 5’ TAATACGACTCACTATAGGG 3’ |  |
