# Supplementary material for: The Mechanism for RNA Recognition by ANTAR Regulators of Gene Expression
Source: PLoS Genet. 2012 Jun 7;8(6):e1002666. doi: 10.1371/journal.pgen.1002666 (PMC3369931; doi:10.1371/journal.pgen.1002666)
Supplement: Text S1 — Supplementary methods. (DOC) [file pgen.1002666.s008.doc]

**Supplementary Methods**

**The mechanism for RNA recognition by ANTAR regulators of gene expression**

Arati Ramesh^1*^, Sruti DebRoy^2*^, Jonathan R. Goodson^3^, Kristina A. Fox^2^, Herbey Faz ^2^, Danielle A. Garsin^2+^, Wade C. Winkler^3+^

^1^Department of Biochemistry, The University of Texas Southwestern Medical Center, Dallas, Texas 75390. ^2^Department of Microbiology and Molecular Genetics, The University of Texas Health Science Center at Houston, Texas 77030. ^3^Department of Cell Biology and Molecular Genetics, The University of Maryland, College Park, Maryland 20742.

*A.R. and S.D. contributed equally to this work.

+Corresponding Authors:

Wade C. Winkler

The University of Maryland

Department of Cell Biology and Molecular Genetics

3112 Biosciences Research Building

College Park, MD 20742

Tel. 301-405-7780

e-mail: [wwinkler@umd.edu](mailto:Wade.Winkler@utsouthwestern.edu)

Danielle A. Garsin

The University of Texas Health Science Center at Houston

Department of Microbiology and Molecular Genetics

6431 Fannin St. MSB 1.168

Houston, TX 77030

Tel. 713-500-5454

Fax: 713-500-5499

e-mail: Danielle.A.Garsin@uth.tmc.edu

**TABLE OF CONTENTS**

Page

3 Supplementary Experimental Procedures: Purification of protein

4 Supplementary Experimental Procedures: Construction of plasmids

**Protein expression and purification**

Hexahistidine-tagged EutV and EutW were expressed as described previously [17]. Coding regions corresponding to ANTAR (residues 132 to 190) and ANTARcc (residues 97 to 190) were cloned in the pHisII vector, transformed in to *E. coli* BL21 (DE3) cells and grown following the same method as for EutV and EutW [17]. Cells were lysed in lysis buffer (25 mM sodium phosphate pH 7.0, 500 mM sodium chloride, 2 mM β-mercaptoethanol, 10% (v/v) glycerol and 1 mM PMSF). Following centrifugation at 12,000 x g for 20 mins, the supernatant was loaded on TALON affinity resin (Clontech) in buffer A (25 mM sodium phosphate pH 7.0, 500 mM sodium chloride). Following washes with 10 column volumes each, of buffer A containing 10 mM imidazole, 20 mM imidazole and 50 mM imidazole, hexahistidine-tagged protein (EutV or EutW) eluted in buffer A with 400 mM imidazole. Purified protein was dialyzed against buffer containing 25 mM sodium phosphate pH 7.0, 150 mM sodium chloride, 2 mM β-mercaptoethanol and 10% (v/v) glycerol and simultaneously digested with Thrombin protease. Digestion for 16 hours at 4°C, at a ratio of 20 mg of EutV or EutW to 1 mg of protease yielded near complete protease digestion. The sample was then subjected to affinity purification over Ni-NTA agarose resin (Qiagen), where purified EutV or EutW were obtained in the flow through and subsequently dialysed against 25 mM Hepes pH 7.0, 150 mM sodium chloride, 2 mM β-mercaptoethanol and 10% (v/v) glycerol. At this stage, measurement of the 260/280nm absorbance ratio revealed a nucleic-acid contamination of EutV but not EutW (Figure S3). To identify the nature of the nucleic-acid contaminant isolated along with EutV, the sample was subjected to DNAse or RNAse A treatment for 20 mins and loaded along with un-treated control sample on a 5% polyacrylamide gel. Following electrophoresis, staining with ethidium bromide revealed that the nucleic acid contaminant was susceptible to DNAse but not RNAse digestion (data not shown). We then used ion-exchange chromatography on a DEAE Sepharose column (GE Health-sciences), to separate free EutV from nucleic-acid bound EutV. Unbound EutV eluted with ~ 250 mM sodium chloride as opposed to nucleic-acid bound EutV which was retained on the column until it was eluted with 350 mM sodium chloride. Fractions containing unbound EutV were dialysed against 25 mM Hepes pH 7.0, 150 mM sodium chloride, 2 mM dithiothreitol and 10% (v/v) glycerol, concentrated using Amicon ultra-filtration devices to ~ 30 µM and stored at -80°C. ANTAR and ANTARcc were purified similarly. Cleavage of the histidine tag was performed using TEV protease. Both ANTAR and ANTARcc did not require additional purification on a DEAE column since the 260/280 nm absorbance ratio was ~0.6 suggesting absence of nucleic acid contaminants.

**Plasmid Construction**

The plasmid pSD2 was used to construct translational *lacZ* fusions that were used in the β-galactosidase assays. An ampicillin resistance marker amplified from pUC18 with SD49 and SD50 was introduced into the KpnI site of pCJK96-2, to generate pSD1. The *lacZ* gene from pCJK47 was amplified using primers SD51 and SD52. The amplified *lacZ* gene and pSD1 was digested with XbaI and XhoI, and ligated together to generate pSD2. The 5' leader region of *eutP* was amplified from pKAF12 using SD1 and SD6 and digested with BamHI and SalI. This was inserted into pSD2 digested with BglII and SalI, to create pSD3. pSD4 and pSD5 was constructed similarly using SD1 and SD2 for pSD4 and SD1 and SD3 for pSD5. The wild-type *eutS* 5' leader region and the *eutS*∆T fragments were amplified from OG1RF genomic DNA with SD42/43 and SD42/45 and cloned into pSD2 as described above to generate pHFZ1 and pHFZ2 respectively. The remaining constructs, pSD6 to pSD13, were constructed using site-directed mutagenesis. The *eutP* 5' leader region was cloned into the pTOPO GW8 vector (Invitrogen). Site-directed mutagenesis was used to alter the cloned *eutP* 5’ UTR. The different mutated versions of *eutP* were amplified using SD1 and SD6, and cloned into pSD2 using the same restriction sites as described above. The constructs were introduced into *E. faecalis* OG1RF and AR2 (∆*eutVW*) mutant by electroporation.
